# Altered expression of ACOX2 in non-small cell lung cancer

**DOI:** 10.1186/s12890-022-02115-7

**Published:** 2022-08-23

**Authors:** Jane S. Y. Sui, Petra Martin, Anna Keogh, Pierre Murchan, Lisa Ryan, Siobhan Nicholson, Sinead Cuffe, Pilib Ó Broin, Stephen P. Finn, Gerard J. Fitzmaurice, Ronan Ryan, Vincent Young, Steven G. Gray

**Affiliations:** 1 Thoracic Oncology Research Group, Laboratory Medicine and Molecular Pathology, Central Pathology Laboratory, St. James’s Hospital, Dublin, D08RX0X Ireland; 2 Department of Medicine, Thoracic Oncology Service, Memorial Sloan Kettering Cancer Center, New York, USA; 3 Midland Regional Hospital Tullamore, Tullamore, Ireland; 4 Department of Histopathology and Morbid Anatomy, Trinity College Dublin, Dublin, Ireland; 5 Department of Histopathology, Labmed Directorate, St. James’s Hospital, Dublin, Ireland; 6 HOPE Directorate, St James’s Hospital, Dublin, Ireland; 7 School of Mathematics, Statistics, and Applied Mathematics, National University of Ireland Galway, Galway, Ireland; 8 Cancer Molecular Diagnostics, Labmed Directorate, St. James’s Hospital, Dublin, Ireland; 9 Surgery, Anaesthesia and Critical Care Directorate, St James’s Hospital, Dublin, Ireland; 10 Department of Clinical Medicine, Trinity College Dublin, Dublin, Ireland; 11 School of Biological Sciences, Technological University Dublin, Dublin, Ireland

**Keywords:** Peroxisome, ACOX2, Acyl-CoA oxidase, Non-small cell lung cancer, Overall survival

## Abstract

Peroxisomes are organelles that play essential roles in many metabolic processes, but also play roles in innate immunity, signal transduction, aging and cancer. One of the main functions of peroxisomes is the processing of very-long chain fatty acids into metabolites that can be directed to the mitochondria. One key family of enzymes in this process are the peroxisomal acyl-CoA oxidases (ACOX1, ACOX2 and ACOX3), the expression of which has been shown to be dysregulated in some cancers. Very little is however known about the expression of this family of oxidases in non-small cell lung cancer (NSCLC). ACOX2 has however been suggested to be elevated at the mRNA level in over 10% of NSCLC, and in the present study using both standard and bioinformatics approaches we show that expression of ACOX2 is significantly altered in NSCLC. ACOX2 mRNA expression is linked to a number of mutated genes, and associations between ACOX2 expression and tumour mutational burden and immune cell infiltration were explored. Links between ACOX2 expression and candidate therapies for oncogenic driver mutations such as KRAS were also identified. Furthermore, levels of acyl-CoA oxidases and other associated peroxisomal genes were explored to identify further links between the peroxisomal pathway and NSCLC. The results of this biomarker driven study suggest that ACOX2 may have potential clinical utility in the diagnosis, prognosis and stratification of patients into various therapeutically targetable options.

**Supplementary Information:**

The online version contains supplementary material available at 10.1186/s12890-022-02115-7.

## Introduction

Lung cancer remains the most common diagnosed malignancy in the world and the most recent Globocan analysis indicates that there will be an estimated 19.3 million new cancer cases worldwide, of which 11.4% will be lung cancer and at 18% the leading cause of cancer death (with an estimated 1.8 million deaths) [1, 3]. Lung cancer itself can be separated into two subtypes small cell lung cancer (SCLC) and Non-Small Cell Lung Cancer (NSCLC), with the vast majority (approximately 85%) falling into the latter subtype. Treatment options for NSCLC are still limited, but recent advances in immunotherapy [4], targeted therapy [5] and the discovery of new actionable mutations [6, 7] have greatly increased the treatment options available for this cancer.

Since their discovery in 1954 [8], peroxisomes have emerged as key metabolic organelles with many diverse functions ranging from cellular lipid metabolism and reactive oxygen species, to non-metabolic roles such as cellular stress responses and synthesis of cellular signalling molecules [9, 10]. These are achieved through interactions with other cellular compartments such as the endoplasmic reticulum and mitochondria [10].

Evidence linking peroxisomes to the biology of NSCLC would stem strongly from the earlier studies demonstrating the roles of peroxisome proliferator-activated receptors (PPARs) in lung cancer tumorigenesis [11]. Moreover, peroxisomes have been linked to cancer via their roles in aberrant metabolism and crosstalk with mitochondria (and associated mitochondrial dysfunction) in cancer [12]. In lung cancer aberrant metabolism is well documented [13, 14], as is mitochondrial dysfunction [15, 16].

One of the main functions of peroxisomes is the processing of very-long chain fatty acids (VLCFAs) via either α- or β- oxidation into metabolites that can be directed to the mitochondria [12]. Peroxisomal acyl-CoA oxidases (ACOs) have been described as the peroxisomal equivalent of the mitochondrial acyl-CoA dehydrogenases (ACADs) [17, 18]. Belonging to the same superfamily, ACOs contain one non-covalently bound Flavin adenine dinucleotide (FAD) per subunit [17, 18]. Similar to mitochondrial ACADs, ACOs catalyse the formation of α-, β- dehydrogenation of acyl-CoA, the initial and rate-determining step of the peroxisomal fatty acid β-oxidation pathway [17, 18]. Depending on the type of very long chain fatty acid (VLCFA) and/or its linear or branched status, this step is carried out by either ACOX1, ACOX2, or ACOX3 [12]. Oxidation of straight-chain fatty acids with different chain lengths is conducted by ACOX1 [12, 19]. ACOX2 and ACOX3 have known roles in the degradation of branched-chain fatty acids [19]. ACOX2 however is the only known ACO associated with bile acid biosynthesis in humans [19].

As such these diverse roles indicate that peroxisomes by their nature play important roles in a large number of globally important human disease including obesity, cancer and age-related disorders [9]. In the disease setting peroxisomes were originally associated with various metabolic disorders [20, 21], and more recently various cancers have been identified as having dysregulation of peroxisomal genes/proteins including prostate, breast and lung cancer [12, 22–25]. In particular alterations to the β-oxidation of peroxisomal fatty acids have been extensively studied in breast cancer [12], and whilst many of the results come from individual stand-alone studies, the upregulation of ACOX1 and other members of this process in cancer cells suggests that the entire β-oxidation metabolic pathway may be affected and linking the entire β-oxidation process to breast cancer tumorigenesis [12].

This pathway has not been studied in such detail in NSCLC, although evidence is beginning to emerge linking the β-oxidation of peroxisomal fatty acids to NSCLC. In the analysis of the peroxisomal pathway in NSCLC by Zhang et al. [25], 38 differentially expressed genes were identified from analyses of The Cancer Genome Atlas (TCGA) NSCLC datasets, but none of the identified genes belonged to the ACOX family. Surprisingly ACOX2 was described as a gene commonly upregulated across several TCGA cancer datasets including NSCLC [12].

As ACOX2 was suggested to be elevated at the mRNA level in over 10% of NSCLC [12], we undertook an analysis of this gene in NSCLC to determine if it had any potential utility as a biomarker in lung cancer at both mRNA and protein level. At the same time using in silico analyses we explored all of the peroxisomal ACOX family members in NSCLC. We show that in contrast to the initial data [12] ACOX2 is predominantly downregulated in NSCLC at both the mRNA and protein level. Decreased ACOX2 expression may be associated with hyper-methylation of individual CpG residues in the ACOX2 promoter. Expression of ACOX2 is associated with prognosis (OS) predominantly in the lung adenocarcinoma (LUAD) subset. We further link altered expression of the other members of both the ACOX family and other peroxisomal proteins that associate with these enzymes to both aberrant expression and prognostic value in NSCLC, and altered ACOX2 expression may be associated sensitivity to certain drugs [12].

## Materials and methods

### Primary tumour samples

Twenty-two surgically resected chemotherapy naïve fresh-frozen tumour specimens were used in this study, taken from surgical resections of at St. James’s Hospital, Dublin during the period 2011–2016. Immediately following resection all samples were evaluated by a pathologist and tumour tissue along with matched normal tissue were dissected for downstream analysis. In total, 11 adenocarcinomas and 11 squamous cell carcinomas were utilized in this study and a summary of the histopathological and clinicopathological data is presented in Table 1. Informed consent was obtained from each patient, and the study was conducted after formal approval from the SJH/AMNCH Hospital Ethics Committee—Ethics REC (No.: 041018/8804).

**Table 1:** Details of surgically resected fresh frozen patient samples used in this study

Sample	Histology	Sex	Age	Stage (7th edition)	TNM
1	Adenocarcinoma	Female	75	IV	pT4 N2 M1a
2	Adenocarcinoma	Male	71	IA	pT1a N0
3	Adenocarcinoma	Female	75	IIA	pT1a N1
4	Adenocarcinoma	Male	71	IB	pT2a
5	Adenocarcinoma	Female	78	IB	pT2a
6	Adenocarcinoma	Female	67	IIIB	pT4 N2
7	Adenocarcinoma	Female	66	IB	pT2a N0
8	Adenocarcinoma	Female	69	IB	pT2a N0
9	Adenocarcinoma	Male	66	IIIA	pT2a N0
10	Adenocarcinoma	Male	86	IIIA	pT3 N1
11	Adenocarcinoma	Male	69	IIIA	pT3 N1
12	Squamous Cell Carcinoma	Female	67	IB	pT2a N0 IB
13	Squamous Cell Carcinoma	Male	71	IB	pT2a N0
14	Squamous Cell Carcinoma	Female	59	IIA	pT2a N1
15	Squamous Cell Carcinoma	Female	66	IIA	pT2a N1
16	Squamous Cell Carcinoma	Male	78	IIA	pT1b N1
17	Squamous Cell Carcinoma	Male	79	IIIA	pT3 N2
18	Squamous Cell Carcinoma	Male	70	IB	T2 N0
19	Squamous Cell Carcinoma	Female	80	IIA	pT2a N1
20	Squamous Cell Carcinoma	Male	72	IIB	pT2b N1
21	Squamous Cell Carcinoma	Male	66	IIIA	pT1b N2
22	Squamous Cell Carcinoma	Female	76	IA	pT1b N0

### Fixed formalin paraffin embedded samples

A total of 204 surgically resected NSCLC tumour specimens from the period 1999–2007 were included in this study. All surgically resected tumour specimens and control specimens were fixed with 10% formalin and embedded in paraffin (FFPE). The Union for International Cancer Control Tumour-Node-Metastasis (TNM) Classification of Malignant Tumours 8th edition was used to stage the tumours [26, 27] and histologically subtyped using the World Health Organization guidelines [28, 29]. A summary of the histopathological and clinicopathological data (including age, sex, smoking status, histology, TNM stage, surgical procedure, tumour grade, and primary site) for the cohort of patients utilized are presented in Table 2.

**Table 2:** Patient characteristics in the SJH NSCLC TMA

	(n =)
LUSC	108
LUAD	82
Pleomorphic carcinoma	7
Large Cell	3
Adenosquamous	4
Female	79
Male	125
Age < 65	92
Age ≥ 65	112
Node Positive	89
Node Negative	115
Tumor Size ≥ 5 cm	82
Tumor Size < 5 cm	122
Grade 1	16
Grade 2	110
Grade 3	78
Stage I	100
Stage II	49
Stage III	54
Stage IV	1
Smoker	100
Ex-Smoker	78
Never Smoker	26

A Beecher Manual Tissue Arrayer (Model MTA-1) was used to generate of a tissue microarray (TMA) containing quadruplicate cores (0.6 mm) of the FFPE embedded samples and a 4 µm sections were subsequently used for immunohistochemistry (IHC) of ACOX2.

### Immunohistochemistry

IHC was performed on TMA sections by utilizing a standard protocol to deparaffinise, rehydrated and wash the slides. Subsequently, ULTRA cell conditioning (ULTRA CC1), pH9.1, was used for heat induced epitope retrieval (HIER). For ACOX2 antibody staining, slides were incubated with rabbit polyclonal primary antibody HPA038280 (Atlas Antibodies, Merck) diluted in ventana antibody diluent (Ref: 251–018) (1:50) for 32 min at ambient temperature and stained using the OptiView™ DAB IHC detection kit on a Ventana BenchMark XT processor.

Following IHC, staining was independently assessed by two pathologists blinded to the clinical, pathological and follow-up data. Staining intensity was designated as either 0, 1 + , 2 + or 3 + and each tumour section was given a H-score between 0 and 300 = 3(% at 3 +) + 2(% at 2 +) + 1(% at 1 +). No samples were observed with a H-Score of 300.

Tumors with high ACOX2 expression were designated as those with an average H score above the median value and low expression below the median. Kaplan–Meier analyses were constructed using Prism 5.01 (GraphPad).

### RNA isolation and qPCR amplification

Total RNA was isolated and converted to cDNA using our previously described methodology [30–32]. Briefly, total RNA was extracted using TRI reagent (Molecular Research Center, Montgomery Road, OH, USA) according to the manufacturer’s instructions. 250 ng of this total RNA was then pre-treated to remove contaminating genomic DNA with amplification grade DNase I (Sigma-Aldrich, St. Louis, MO, USA) according to the manufacturer’s instructions [30–32]. All-in-One cDNA Synthesis SuperMix (Bimake, W Sylvanfield Drive, Houston, TX, USA) was then used to generate first strand cDNA according to the manufacturer’s instructions [30–32].

qPCRs for ACOX2 (relative quantification method) were subsequently conducted on these samples using an Illumina Eco qPCR and 2 × SYBR Green qPCR Master Mix (Bimake, W Sylvanfield Drive, Houston, TX, USA) using the manufacturers protocol in a 2-step qPCR program with histone H3 as the internal control using the following primers:

ACOX2 FWD: 5’– CCAAGTGGACATGGCAAGAA – 3’

ACOX2 REV: 5’ – GTGACTTCTGAGCCCACTGGA – 3’

Histone H3 FWD: 5’ – GGTAAAGCACCCAGGAAGCA – 3’.

Histone H3 REV: 5’ – CCTCCAGTAGAGGGCGCAC– 3’.

The following cycling parameters were used:

An initial Polymerase activation of 95 °C for 2 min followed by 35 cycles of 95 °C 15 s and annealing/amplification 61 °C for 1 min. A melting curve analysis was conducted at the end of each PCR using 95 °C 15 s, 55 °C 15 s and a final 95 °C for 15 s. The final concentration of primer in each reaction was 200 nM, and the.

Data was analysed using either the default in-built Eco software or imported into EcoStudy (Illumina) for analysis using the ΔΔCq method [33].

### Identification of differential expressed genes for acyl-CoA oxidases in NSCLC

To determine if acyl-CoA oxidases show significantly altered expression in NSCLC a systematic analysis was carried out using Lung Cancer Explorer (LCE) [34] to conduct a meta-analysis of standardized mean difference (tumour vs normal) in gene expression, and subsequently a meta-analysis of survival and gene expression association based on univariate Cox Proportional-Hazards Model.

### Establishment of protein–protein interaction networks associated with acyl-CoA oxidases

The STRING database [35] was interrogated to conduct a functional enrichment analysis of acyl-CoA oxidases to identify Protein–Protein Interaction Networks, exported to Cytoscape [36] and first neighbours highlighted. All identified candidates were subsequently interrogated for both differential gene expression and survival associations using LCE [34].

### Validations of the expression differences of the differentially expressed genes associated with acyl-CoA oxidases

Validations of altered ACOX2 expression were examined in the TCGA NSCLC cohorts using LCE. Meta—analyses of additional gene expression datasets were also conducted using LCE [34]. Tumour—Normal Gene expression differences were calculated by LCE by meta-analysis of standardized mean difference (tumour—normal) using Hedges’ G as an effect size metric. For this all studies included for meta-analysis must have at least 10 samples in each group and meta-analysis was only performed for genes with data available from at least three qualifying studies. The analysis was performed separately for lung adenocarcinoma and squamous cell carcinoma [34]. Survival association with gene expression involved meta-analysis of survival and gene expression associations based on univariate Cox Proportional-Hazards modelling. For each included study, gene expression was normalized to zero mean and unit variance for each gene prior to model fitting. Only studies that had survival data for at least 10 samples were included in the meta-analysis which was only performed for genes if data was available from at least three qualifying studies [34].

Altered protein expression in the TCGA-LUAD was validated using UALCAN [37] to interrogate the CPTAC discovery dataset. This dataset comprises 111 tumors, (with 102 tumors paired with normal adjacent tissue samples) which have global proteome and phospho-proteome data available for interrogation [38].

### Prognostic value for acyl-CoA oxidases and other associated differentially expressed genes

To examine for prognostic value, five separate analyses were conducted using either KM-Plot (Microarray and RNA-Seq) [39], LCE [34], UALCAN [37] and OncoLnc [40]. LCE, UALCAN and OncoLnc all utilise TCGA-LUAD and TCGA-LUSC datasets. All analyses used the default settings to assess prognostic survival as follows: KM-Plot (median); LCE (median); UALCAN (High vs Medium/Low); OncoLnc (50:50 percentiles).

All analyses use the Cox proportional hazards model.

### Effects of copy number variations (CNV) on acyl-CoA oxidases

To examine whether the altered expressions of acyl-CoA oxidases (ACOX1-3) were correlated with CNVs, the datasets for TCGA-LUAD and TCGA-LUSC were analysed through cBioPortal [41, 42].

### Associations with mutated genes

To identify mutated genes from the associated TCGA datasets that result in changes of expression of USO1 in NSCLC an analysis was conducted using the MuTarget platform with ACOX2 set as the target gene, and with mutation prevalence set at 2% [43]. All targets identified were subsequently retested using correlation analysis on TIMER2.0 [44].

### Methylation analysis on acyl-CoA oxidases

Correlations between DNA methylation and altered ACOX2 expression were examined initially using UALCAN [37], and subsequently with MEXPRESS [45] and WANDERER [46] using default settings.

### Correlations between ACOX2 expression and immune infiltrations in NSCLC

To interrogate how altered expression of ACOX2 expression was associated with tumour infiltrating immune cells (TIICs) in NCSLC we used TIMER [47] or TIMER2 [44]. The gene expression levels against tumour purity were calculated, and assessments were calculated based on the purity-corrected partial Spearman’s rho value with associated statistical significance. For analysis in GEPIA2 [48], a similar strategy was employed correlating ACOX2 expression with a six gene T-Cell exhaustion signature selecting for Spearman’s correlation coefficient as the default setting. GEPIA2 utilizes the non-log scale for calculation of significance. Finally, the ESTIMATE score [49] was utilized to show the immune score and tumour purity.

### Correlations between ACOX2 expression and anti-cancer drug sensitivity in NSCLC cell lines

The relative expressions of ACOX2 in NSCLC cell lines and associated pharmacological profiles for 24 anti-cancer drugs were downloaded from the Cancer Cell Line Encyclopaedia (CCLE) database [50]. The half maximal inhibitory concentrations (IC50) values of 24 anticancer drugs in n = 89 NSCLC cell lines and a non-parametric correlation (Spearman) between ACOX2 gene expression and IC50 was conducted. For any drugs for which correlations were observed, the cell lines were subsequently divided into “sensitive” and “resistant” groups using a drug IC50 of 8 µM as the threshold according to Xiang et al. [51]. The expression differences for ACOX2 in these two groups were evaluated using a non-parametric Mann–Whitney test.

A further analysis for drugs potentially capable of targeting NSCLC based on ACOX2 expression was conducted using the DepMap PRISM repurposing Primary Screen [52].

### Statistical analysis

All data are expressed as mean ± SEM unless stated otherwise. Statistical analysis was performed with Prism 5.01 (GraphPad, Ca, USA) using either paired two-tailed Student’s t-test or the Mann–Whitney two-tailed t-test. Correlations were conducted using the Spearman non-parametric correlation coefficient. Differences were considered significant when p < 0.05. All other analyses through web based servers used the default settings in each instance.

## Results

### Identification of ACOX2 as differentially expressed genes in NSCLC.

**Fig. 1:**
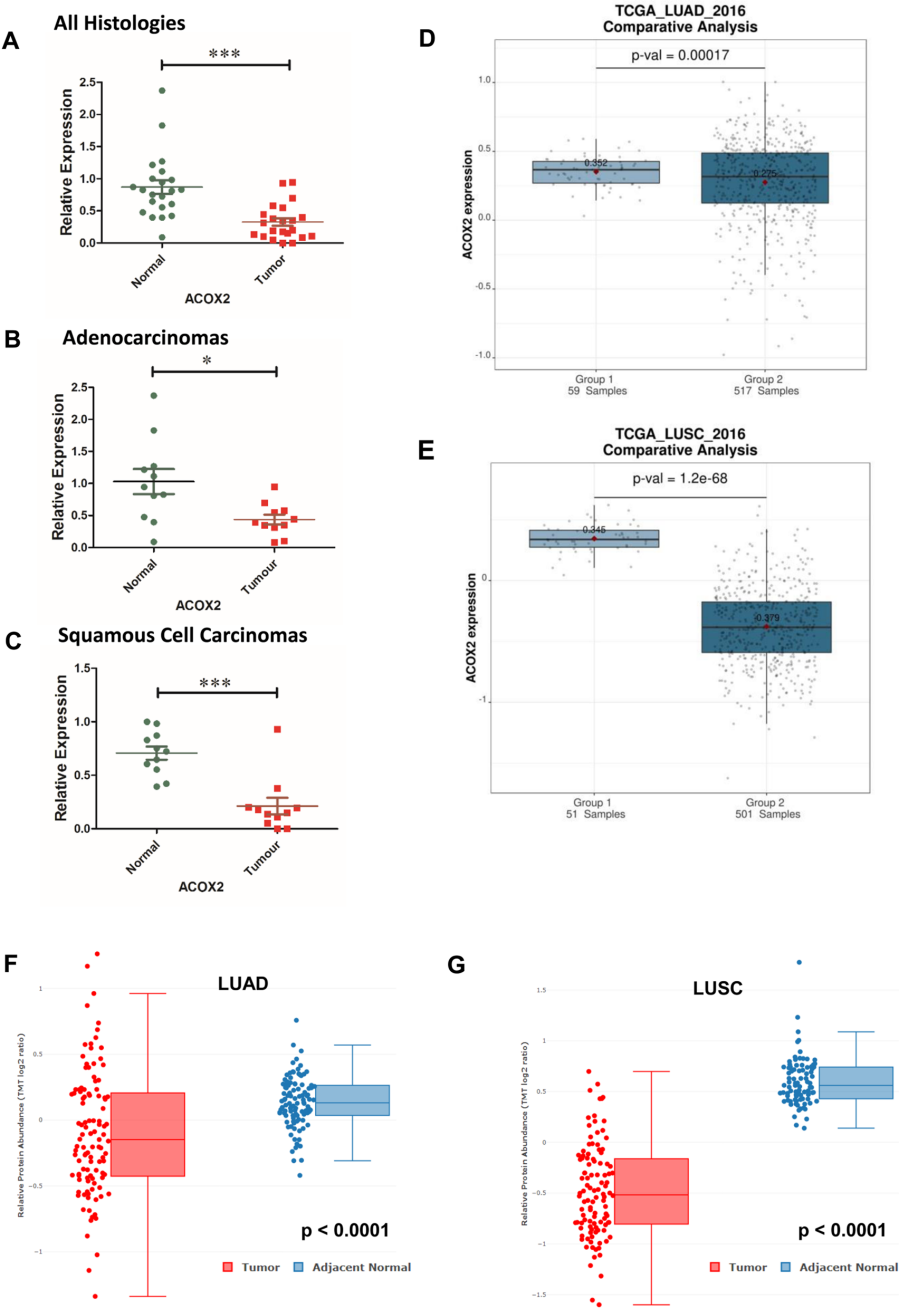
Altered expression of ACOX2 in NSCLC. Identification of decreased expression of ACOX2 in NSCLC. Examination of changes to ACOX2 mRNA levels in fresh-frozen surgically resected patient samples comprising **A** All Histologies; **B** Adenocarcinomas alone and **C** Squamous Cell Carcinomas. **D** Confirmatory comparative analysis of ACOX2 mRNA levels in the Cancer Genome Atlas (TCGA) Lung Adenocarcinoma (LUAD) and **E** the Lung Squamous Cell Carcinoma (LUSC) datasets using Lung Cancer Explorer (LCE) (19). **F** Altered expression of ACOX2 total protein levels in LUAD as assessed using cProSite. ***p < 0.001. **G** Altered expression of ACOX2 total protein levels in LUSC as assessed using cProSite. ***p < 0.001

We assessed the expression ACOX2 mRNA in a panel of surgically resected fresh-frozen normal/tumour matched patient samples from Stage I and II patients by qPCR (Fig. 1A). Overall, levels of ACOX2 were shown to be significantly decreased across all samples (p < 0.0001). When stratified according to histology, downregulation of ACOX2 was found to be significant in both Adenocarcinomas (LUAD) (p = 0.0151) and Squamous Cell Carcinomas (LUSC) (p = 0.0008) respectively (Fig. 1A–C). We subsequently interrogated the TCGA LUAD and LUSC datasets for altered expression of ACOX2. In agreement with our data, significantly decreased expression of ACOX2 mRNA was observed for both LUAD (Fig. 1D; p = 1.7 × 10^–4^) and LUSC (Fig. 1E; p = 1.2 × 10^–68^) respectively. The other acyl-CoA oxidases were found to have significantly decreased expression of ACOX1 mRNA and ACOX3 mRNA in the LUSC samples only (Additional file 1: Table S1).

A further meta-analysis of a larger number of gene expression datasets was conducted, using a systematic analysis (Additional file 1: Table S2—Tumour vs Normal). From this analysis decreased expression of ACOX2 mRNA was found to be only significantly altered at the mRNA level in LUSC (Additional file 1: Table S2—p = 0.0031). A meta-analysis of the same datasets for altered ACOX2 mRNA expression between Tumour/Normal sample was conducted, and in this analysis ACOX2 mRNA is not associated with any mRNA differences overall—Additional file 6: Figure S5A. However, when separated according to histology LUAD mRNA remains non-significant (Additional file 7: Figure S6, p = 0.6), whilst LUSC remained significantly differentially expressed (Additional file 7: Figure S6, p = 0.0014).

We subsequently interrogated the CPTAC dataset for LUAD (23) and the results show that ACOX2 protein levels are significantly reduced in tumour samples compared to normal (Additional file 1: Table S1). At the time of writing the CPTAC data for LUSC is unavailable for analysis in UALCAN. However, access to the proteomic data for both LUAD and LUSC can now be found at cProSite. Using this we assessed the expression of ACOX1-3 in both LUAD and LUSC and the data is presented in Additional file 1: Table S1. From this analysis, levels of ACOX1 were not significantly altered in LUAD and LUSC. ACOX2 however was significantly downregulated at the protein level in both LUAD (p < 0.0001, Fig. 1F) and LUSC (p < 0.0001, Fig. 1G). Finally, ACOX3 protein was found to be significantly downregulated in LUAD for paired samples, whereas non-paired samples had no significant change to their overall protein levels, while ACOX3 protein was only significantly downregulated in LUSC (Additional file 1: Table S1).

### Altered expression of acyl-CoA oxidases and other peroxisomal genes in NSCLC

Using STRING we identified a series of protein–protein interactions for acyl-CoA oxidases (ACOX1-3). The resulting network of first neighbours is presented in Additional file 2: Figure S1. Using systematic analysis in LCE we examined these genes for significant alterations in expression between tumour and normal lung, and the results are presented in Additional file 1: Table S2. This meta-analysis is presented as the standardized mean difference of Tumour-Normal gene expression using Hedges’ G as an effect size metric [34]. From this analysis significant alterations were observed for ACOX1 (p.adj—0.0011; LUAD); ACOX2 (p.adj—0.0031; LUSC); ACOX3 (p.adj—0.0011; LUSC). Other significant associations were observed for ACOXL, ACAA1, ACADM, ACOT4, ACSL1, ACSL5, AMACR, CAT, CRAT, DECR2, EHHADH, GNPAT, HSD17B4, PEX5, PEX14 and SCP2 (Additional file 1: Table S2). Of these, CAT, HSD17B4 and ACAA1 have recently been identified as showing either altered expression, or prognostic significance, or both in NSCLC [25].

### Potential prognostic value of acyl-CoA oxidases and other peroxisomal genes in NSCLC

**Fig. 2:**
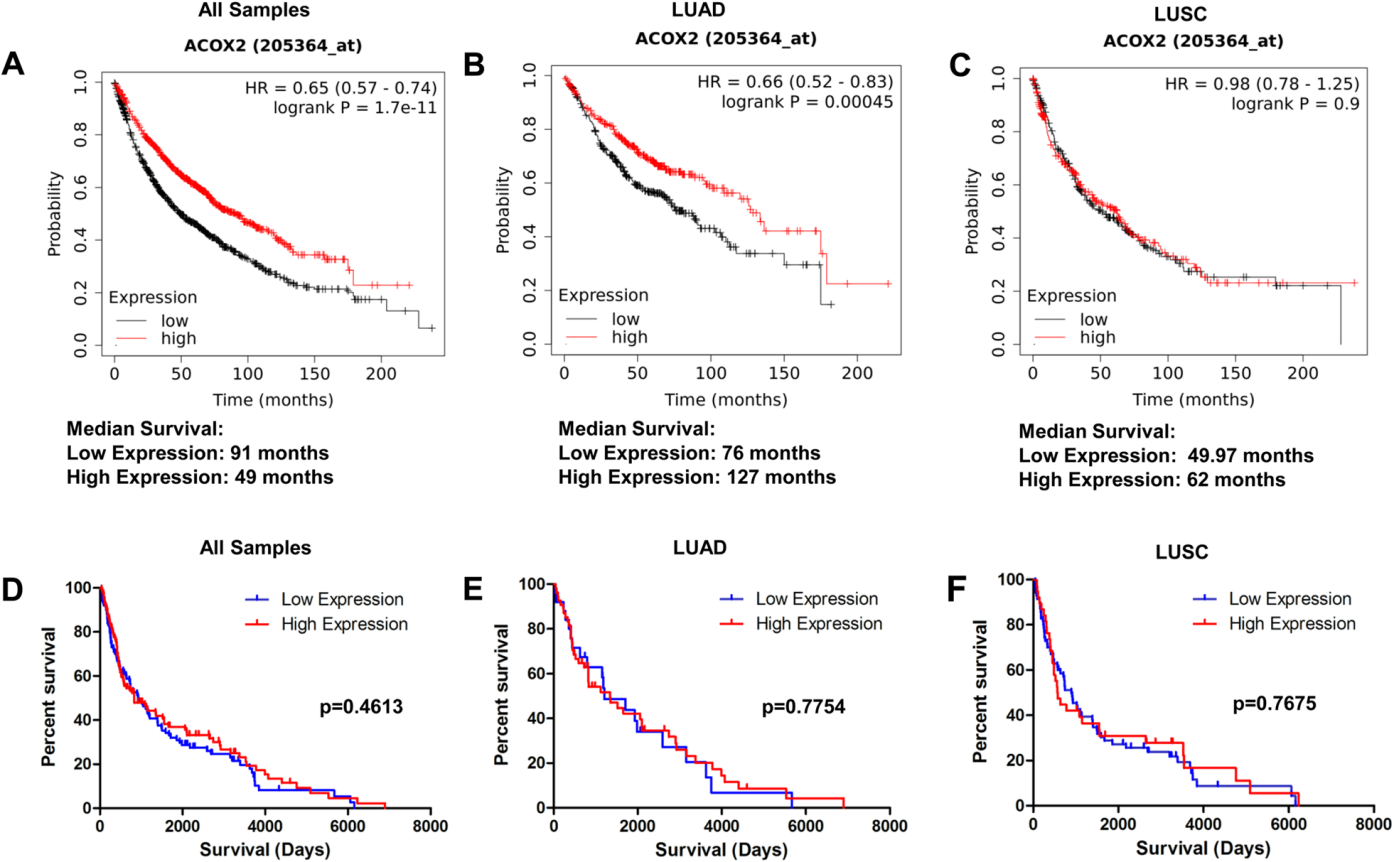
Prognostic value of ACOX2 in NSCLC. The prognostic value of ACOX2 expression was assessed for overall survival (OS) using KM-Plotter [39] and by IHC on a patient TMA. Higher expression of the mRNA for ACOX2 was associated with better OS overall (**A**); which when stratified by tumour histology was limited to the LUAD subtype (**B**); whilst no difference in OS was observed for LUSC (**C**). In contrast no significant OS benefit was observed by IHC for high ACOX2 protein expression overall (**D**); or in LUAD (**E**); or LUSC (**F**). p < 0.05 was considered to be significant

An initial analysis of the prognostic value of ACOX2 was conducted using KM-Plot [39]. Overall high expression of ACOX2 mRNA was associated with better overall survival (OS) in NSCLC (Fig. 2A). When the analysis was stratified according to histological subtype, this OS benefit was restricted solely to LUAD (Fig. 2B) and was not seen in LUSC (Fig. 2C). We subsequently examined the expression of ACOX2 in patient samples by IHC. In this regard no OS benefit was observed for ACOX2 overall or in any histological subtype (Fig. 2D–F; Additional file 3: Figure S2), suggesting that the observed OS benefit is restricted to mRNA expression (Additional file 4: Figure S3).

Using LCE, we conducted a systematic analysis of ACOX2 mRNA for OS benefit. Overall, ACOX2 mRNA was found to have OS benefit in LUAD (p = 0.00049) but not in LUSC (Additional file 1: Table S3), while ACOX1 or ACOX2 mRNA had no survival benefit. However, when progression free survival (PFS) was assessed, ACOX2 mRNA was not found to show any significant PFS benefit (Additional file 5: Figure S4), whereas both ACOX1 and ACOX3 mRNA had significant associations with PFS, indicating that ACOX2 mRNA is solely linked with OS benefit. A meta-analysis of the same datasets for ACOX2 mRNA shown for all histologies for expression differences (Additional file 6: Figure S5A), and in this analysis ACOX2 mRNA is associated with OS (p < 0.01—Additional file 6: Figure S5B).

To evaluate the potential prognostic value of the acyl-CoA oxidases and their identified first neighbours, a systematic analysis was initially conducted using LCE and the results are provided in Additional file 1: Table S3. In agreement with Zhang et al., we confirm that altered expression of ACAA1 and CAT were associated with prognostic value in LUAD [25]. However, systematic analysis in LCE did not demonstrate any prognostic value for HSD17B4 in contrast to that previously observed [25]. Along with these genes, other genes which appear to show prognostic value in our analysis included ACOX2, ACOXL, ACOT4, ACSL5, AGPS, HADH and SCP2 (Additional file 1: Table S3). We subsequently compared OS values across five different database analyses (Additional file 1: Table S4). Those genes showing significant prognostic OS value were predominantly linked to the LUAD subtype (Additional file 1: Table S4), but some analyses suggest that altered expression of certain peroxisomal genes are associated with OS prognostic value in LUSC (for example ACOXL, ACSL5 and others in Additional file 1: Table S4). ACOXL and AGPS were significant for LUAD across all datasets/analyses, whilst CAT was significant across four of five (Additional file 1: Table S4). From this analysis we chose ACOX1, ACOX2, ACOX3, ACOXL, AGPS and CAT for further evaluation in LUAD using KM-Plot, and the results are shown in Additional file 4: Figure S3. Surprisingly, ACOX2 mRNA was not found to have any association with PFS (Additional file 5: Figure S4), whereas both ACOX1 and ACOX3 mRNA had significant associations with PFS alongside ACOXL, AGPS and CAT (Additional file 1: Table S5). From these analyses, high expression (stratified based on the median) of all 6 mRNAs is associated with significantly better OS in LUAD, and warrant further assessment.

### Influence of Copy Number Variations on the expression of acyl-CoA oxidases in NSCLC

**Fig. 3:**
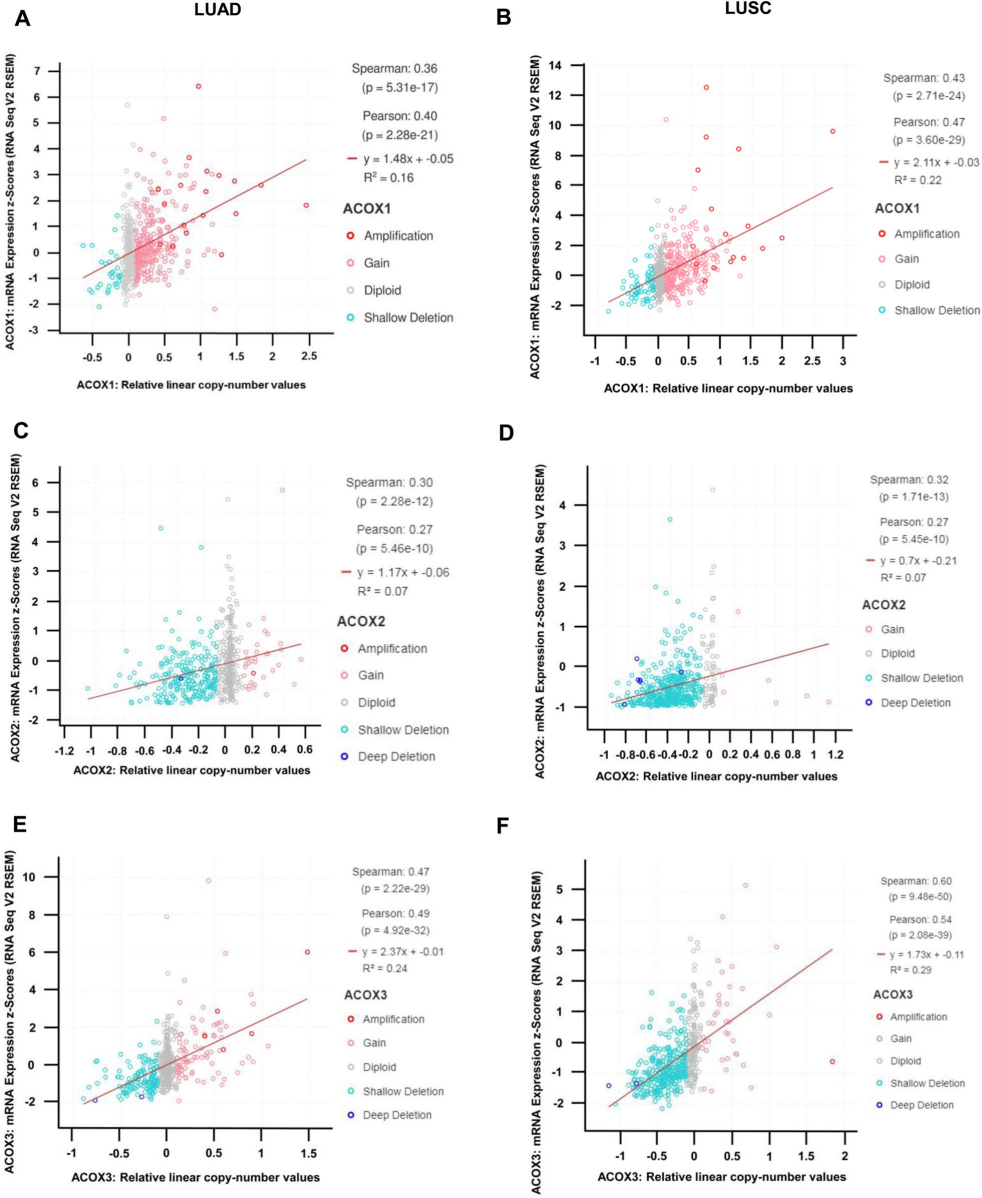
Correlations between acyl-CoA oxidase expression and copy number value in NSCLC. Correlations between acyl-CoA oxidase expression and copy number value (CNV) were examined using cBioPortal [41, 42]. Significant positive correlations between expression and CNV were observed in LUAD and LUSC for ACOX1 (**A**, **B**), ACOX2 (**C**, **D**) and ACOX3 (**E**, **F**). Spearman and Pearson correlation analyses were used and p < 10^–5^ was considered significant

As acyl-CoA oxidases are significantly dysregulated in NSCLC (Additional file 1: Table S1), to further study the potential effects of this dysregulation we used cBioPortal [41] to assess for any correlations between copy number variations (CNVs) and gene expression changes in the TCGA –LUAD and –LUSC datasets. As shown in Fig. 3, positive correlations between CNV and gene expression were observed for all genes in both LUAD (Fig. 3A, C, and E) and LUSC (Fig. 3B, D, and F).

### Correlations between ACOX2 expression and mutations in NSCLC

We subsequently assessed if the significant dysregulation of ACOX2 could be linked to the mutational status of the tumours, using TIMER2.0 we examined a number of genes commonly mutated in lung cancer (e.g. TP53) to determine whether mutations within these key genes were correlated with altered ACOX2 expression levels and the results are presented in Table 3. Of the genes examined, mutations in TP53, KRAS and CDKN2A were significantly correlated with altered ACOX2 expression for both LUAD and LUSC. Mutated ROS1 was significantly associated with altered ACOX2 expression only in LUAD, while mutated ERBB2 and ALK were associated with altered ACOX2 in LUSC.

**Table 3:** Correlations between ACOX2 mRNA expression and mutation of key genes in NSCLC. Gene Expression Correlations

	Mutated Gene	LUAD		LUSC	
		Partial cor	*p-value*	Partial cor	*p-value*
ACOX2	TP53	− 0.272	**1.8 × 10**^**–10**^	− 0.321	**0.0073**
	KRAS	0.168	**0.00035**	0.929	**0.0042**
	EGFR	0.168	**0.012**	0.929	0.66
	ERBB2	0.022	0.93	− 1.112	**0.0091**
	PIK3CA	− 0.158	0.13	− 0.137	0.3
	ALK	− 0.126	0.068	− 0.513	**0.035**
	ROS1	− 0.358	**0.0014**	0.165	0.067
	CDKN2A	− 0.247	**0.044**	− 0.251	**0.049**
	PTEN	− 0.192	0.83	− 0.014	0.61
	BRAF	− 0.07	0.37	− 0.378	0.25
	MET	− 0.303	0.06	0.109	0.56
	NF1	− 0.017	0.49	0.094	0.5

Analysis was conducted using TIMER 2.0 [44]

Any correlates/analyses meeting the threshold for significance (*p* < 0.05) have been highlighted in bold text

**Fig. 4:**
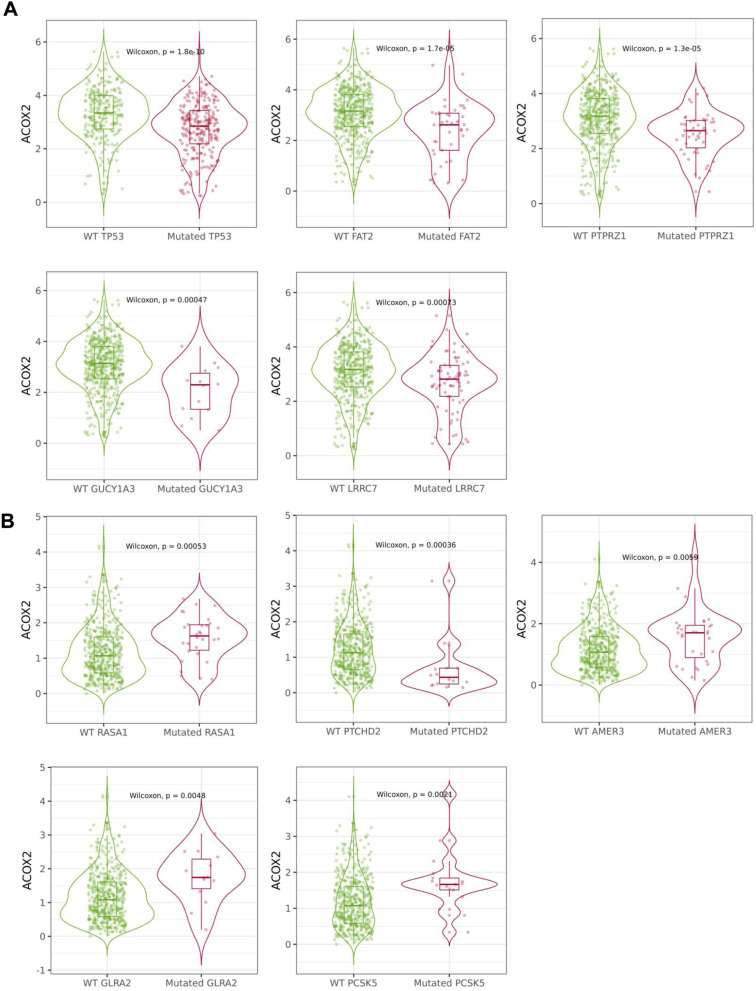
Identification of mutated genes which affect ACOX2 expression in NSCLC. ACOX2 gene expression changes and mutation status in NSCLC were examined using muTarget [43]. The resulting analysis identified several genes which if were mutated resulted in significantly altered ACOX2 expression as follows: **A** TP53, FAT2, PTPRZ, GUCY1A3 and LRRC7 in LUAD and **B** RASA1, PTCHD2, AMER3, GLRA2 and PCSK5 in LUSC. All results were reassessed and plotted as shown using TIMER2 [44]

Finally, using muTarget [43], we then analysed whether mutations in any other genes may affect ACOX2 expression in LUAD and LUSC. From this analysis 244 genes were identified in LUAD which if mutated resulted in a significant alteration in ACOX2 expression, while 35 genes were identified in LUSC (Additional file 1: Tables S6 and S7) and the results of the top five mutated genes as defined by muTarget that affect ACOX2 expression were validated using TIMER2.0 and are presented in Fig. 4 for both LUAD (TP53; FAT2; PTPRZ1; GUCY1A3 and LRRC7—Fig. 4A) and LUSC (RASA1; PTCHD2; AMER3; GLRA2 and PCSK5—Fig. 4B).

### Methylation analysis of ACOX2

**Fig. 5:**
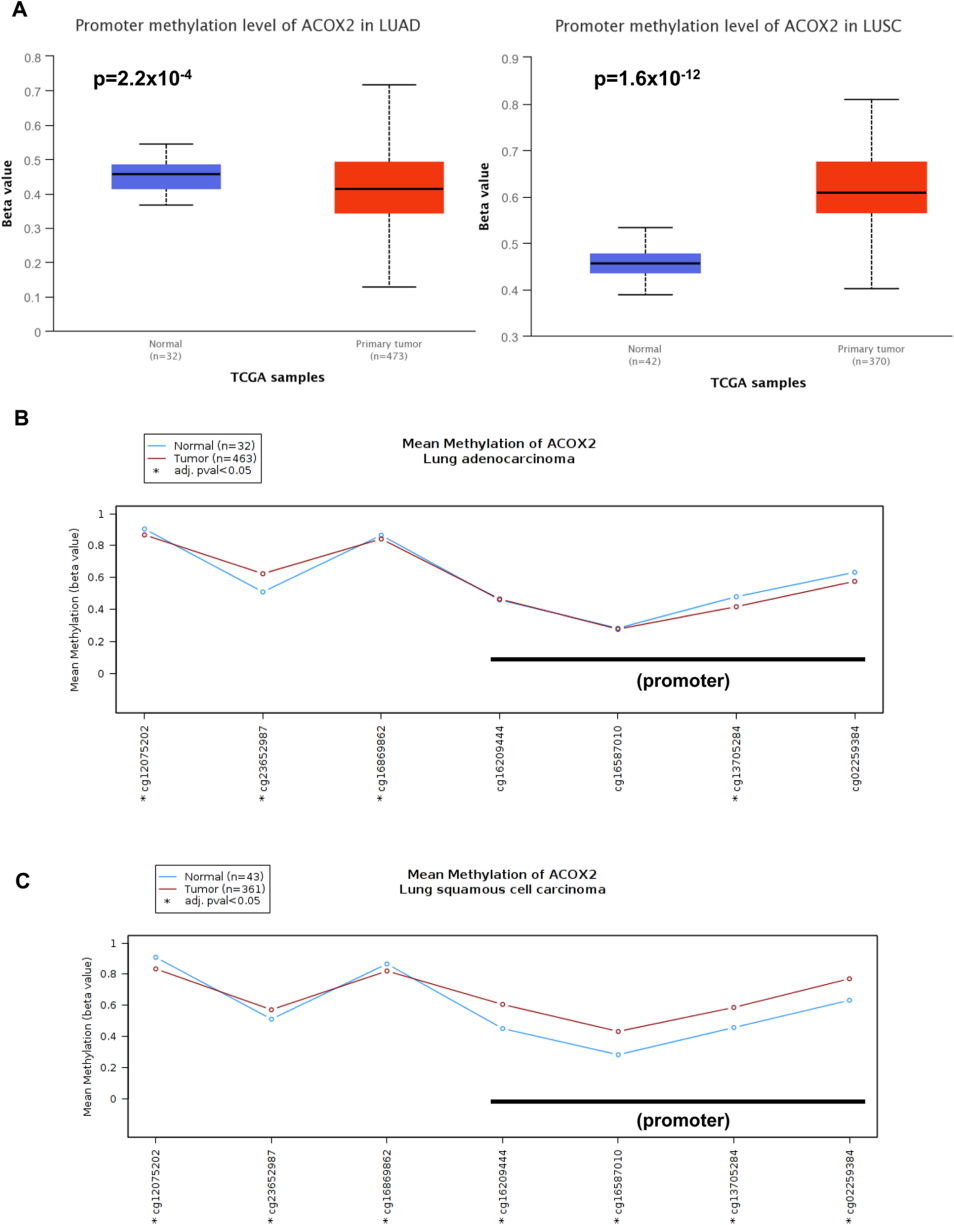
Methylation analysis of the ACOX2 promoter in NSCLC. The potential role of DNA CpG methylation was examined in LUAD and LUSC. **A** Analysis of methylation changes using UALCAN [37] and using stratification according to the default Beta-value cut-off for hypermethylation (0.7–0.5) the results suggest that hypermethylation is a significant element in LUSC, but not LUAD. Re-evaluation of methylation at the ACOX2 promoter in LUAD (**B**) and LUSC (**C**) using TCGA-Wanderer [46], showing clear differences in methylation at the promoter in LUSC. (*p < 0.05 is considered significant)

As ACOX2 is significantly downregulated in both LUAD and LUSC using UALCAN we next examined whether methylation may be an important element in the downregulation of this gene. While significant methylation is presented for both LUAD (Fig. 5A) and LUSC (Fig. 5B), the required Beta-value cut-off for hypermethylation is (0.7–0.5) [53, 54] is only present for LUSC suggesting that DNA hypermethylation is a significant element in LUSC, but not LUAD (Fig. 5). UALCAN methylation analysis involves the Illumina 450 k Infinium chip and the associated CG probes showing significance were (CG16587010, CG13705284 and CG02259384). We then re-assessed methylation at this gene using MEXPRESS [45]. Analysis found significant negative correlations between methylation and gene expression particularly within CpG residues of the ACOX2 promoter (Additional file 1: Table S8). To assess this in more detail we re-analysed methylation using TCGA-Wanderer [46]. Selecting the Illumina 450 K settings the analysis found that the majority of probes in the LUAD dataset did not show significance with the exceptions of cg13705284, cg12075202, and cg23652987 (Fig. 5B and Additional file 1: Table S8) whereas all probes remained significant in the LUSC dataset (Fig. 5C and Additional file 1: Table S8). These results suggest that the downregulation of ACOX2 particularly in LUSC may be due in part to increased DNA CpG methylation within the ACOX2 promoter.

### Effects of decreased ACOX2 expression on immune infiltration

To assess the potential impact of decreased ACOX2 expression on tumour immunity, an analysis of tumour-infiltrating immune cells in NSCLC was conducted using TIMER [47]. Following purity-adjustment, spearman's rho and significance for the six immune cell types were generated and the results are presented in Table 4a. The results suggest that decreased expression of ACOX2 was negatively correlated with CD8 + T cell and Neutrophil infiltration in LUAD (Table 4a), whereas in contrast, decreased ACOX2 expression in LUSC was significantly positively associated with immune cell infiltration for all six immune cell types examined (Table 4a). Subsequently, when USO1 expression and immune cell infiltrates were examined for correlations with survival, only B-cell and dendritic cell immune infiltrates had survival benefit and this was further limited to LUAD only (Table 4b).

**Table 4:** Correlations between ACOX2 and immune infiltrations in NSCLC

(a) Gene Correlations					
	Variable	LUAD		LUSC	
		Partial cor	*p-value*	Partial cor	*p-value*
ACOX2	Purity	0.060729633	0.17778443	− 0.387756565	**1.34 × 10**^**–18**^ ***
	B Cell	0.061510052	0.176694922	0.144611112	0.001632704**
	CD8 + T cell	− 0.123441444	0.006380587**	0.237579471	1.61 × 10–7***
	CD4 + T cell	0.064108492	0.159075162	0.351217599	3.09E − 15***
	Macrophage	0.001779846	0.968813906	0.458092406	4.03E − 26***
	Neutrophil	− 0.092574269	0.04198932*	0.361723863	3.68E − 16***
	Dendritic cell	0.017626312	0.69771296	0.423935124	4.25E − 22***

(B) Survival			
	Variable	LUAD	LUSC
		*p-value*	*p-value*
ACOX2	B Cell	**0.000268** **	0.778204
	CD8 + T Cell	0.345905	0.370702
	CD4 + T Cell	0.507773	0.142871
	Macrophage	0.110109	0.651048
	Neutrophil	0.081069	0.126999
	Dendritic Cell	**0.047524** *	0.324067
	USO1	0.091429	0.240999

(Analysis was conducted using TIMER [47]). Results are presented as purity-corrected partial Spearman’s rho value and statistical significance

Any correlates/analyses meeting the threshold for significance (*p* < 0.05) have been highlighted in bold text

*p < 0.05; **p < 0.01; ***p < 0.001

Partial Cor.—partial correlation (partial Spearman’s rho value) (Analysis was conducted using TIMER). Results are presented as purity-corrected partial Spearman’s rho value and statistical significance

*p < 0.05; **p < 0.01; ***p < 0.001. Partial Cor. partial correlation (partial Spearman’s rho value)

We re-assessed the effects of ACOX2 on immune infiltrates using TIMER2 [44], which provides a provides a more robust estimation of immune infiltration levels for The Cancer Genome Atlas (TCGA) datasets by using six state-of-the-art algorithms (TIMER, xCell, MCP-counter, CIBERSORT, EPIC and quanTIseq), and the results are presented in Additional file 1: Tables S9 and S10.

**Fig. 6:**
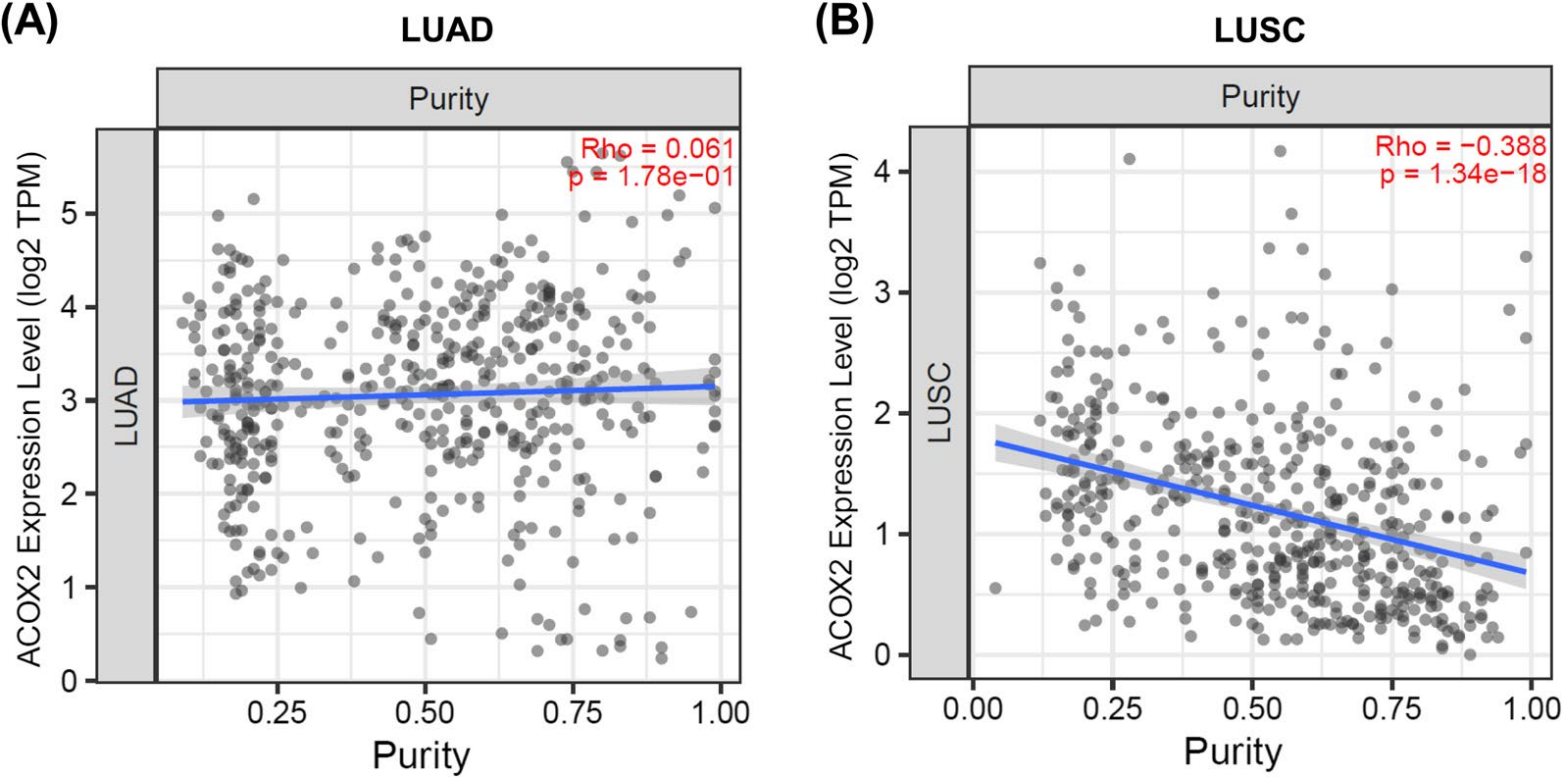
Associations between ACOX2 expression and tumour purity. Associations between Tumour Purity and ACOX2 expression were generated using Timer2 [44] for **A** LUAD and **B** LUSC

Finally, the ESTIMATE package [49] was used to generate an Estimate score (inferring tumour purity linked to gene expression). The Estimate scores (StromalScore, ImmuneScore, ESTIMATEScore, TumorPurity) are provided in Additional file 1: Table S11), and the graphed using TIMER2 [44, 55] as shown in Fig. 6.

### Correlation analysis between ACOX2 expression and immune cell exhaustion

To investigate the relationship between ACOX2 we used TIMER2.0 to assess correlations between its expression and the expression of important markers of T cell exhaustion [44, 56]. The markers chosen were PD-1 (PDCD1), CTLA4, LAG3, TIM-3 (HAVCR2), and GZMB and the results of this analysis are presented in Additional file 1: Table S12. After correlation adjustment by purity, ACOX2 expression was negatively correlated with the expression levels of PD-1, LAG3 and GZMB in LUAD (Additional file 1: Table S12), while its expression was positively correlated with PD-1, CTLA4 and TIM-3 in LUSC (Additional file 1: Table S12). A second assessment of T cell exhaustion was carried out using GEPIA2 [48] and a pre-defined set of T cell exhaustion markers (PDCD1, HAVCR2, TIGIT, LAG3, CXCL13 and LAYN). A similar pattern to that observed in TIMER was observed where a negative correlation between this 6 gene signature and ACOX2 expression with T cell exhaustion occurs in LUAD (r =  − 0.15, p = 0.001) with a positive correlation observed in LUSC (r = 0.23, p = 4.4 × 10^−07^).

### Correlation analysis between ACOX2 expression and tumour mutational burden

Tumour mutational burden (TMB) is widely considered to be a biomarker for predicting potential patient response to immune checkpoint inhibitor therapy [57, 58]. Using the methodology described by Feng and Shen [59], we analysed the correlation between ACOX2 expression and biomarkers of tumour burden mutation, genes associated with either the DNA Damage Response (DDR) pathway or the Mismatch Excision Repair (MMR) pathway as a proxy for TMB (Table 5). The combined signatures for DDR pathway found a small significant negative correlation in both LUAD (p = 0.02), a very significant negative correlation was observed for LUSC (3.2 × 10^–21^). The signature for genes associated with MMR showed no correlations in LUAD, but again had a very significant negative correlation in LUSC (3.2 × 10^–19^).

**Table 5:** Correlation between ACOX2 expression and markers of Tumour Mutational Burden

	Variable	LUAD		LUSC	
		R	*p-value*	R	*p-value*
DNA Damage Response (DDR) Pathway	BRCA1	− 0.15	**3 × 10**^**–4**^ ***	− 0.42	**3.5 × 10**^**–24**^ ***
	ATM	0.03	0.49	0.12	**0.0068** **
	ATR	0.16	**0.00023** ***	− 0.25	**4.3 × 10**^**–9**^ ***
	CDK1	− 0.24	**2.2 × 10**^**–08**^ ***	− 0.38	**5.7 × 10**^**–20**^ ***
	CHEK1	− 0.2	**2.5 × 10**^**–06**^ ***	− 0.35	**6.6 × 10**^**–17**^ ***
	CHEK2	− 0.047	0.27	− 0.47	**6.7 × 10**^**–31**^ ***
	TP53	0.18	**3.7 × 10**^**–05**^ ***	− 0.096	**0.027** *
	Combined Signature	− 0.1	**0.02** *	− 0.37	**3.2 × 10**^**–21**^ ***
Mismatch excision repair (MMR) related genes	PMS2	0.091	**0.034** *	− 0.29	**1.3 × 10**^**–11**^ ***
	MLH1	0.22	**2.4 × 10**^**–07**^ ***	0.21	**1.3 × 10**^**–06**^ ***
	MSH2	− 0.051	0.24	− 0.36	**1.7 × 10**^**–17**^ ***
	MSH3	0.099	**0.021** *	− 0.02	0.65
	MSH6	− 0.035	0.42	− 0.44	**4.8 × 10**^**–27**^ ***
	PCNA	− 0.19	**5.6 × 10**^**–06**^ ***	− 0.43	**4.7 × 10**^**–26**^ ***
	Combined Signature	0.0013	0.98	− 0.37	**3.2 × 10**^**–19**^ ***

Analysis was conducted using GEPIA2 [48]. Results are presented as Spearman’s rho value (R) alongside statistical significance

Any correlates/analyses meeting the threshold for significance (*p* < 0.05) have been highlighted in bold text

*p < 0.05; **p < 0.01; ***p < 0.001

**Fig. 7:**
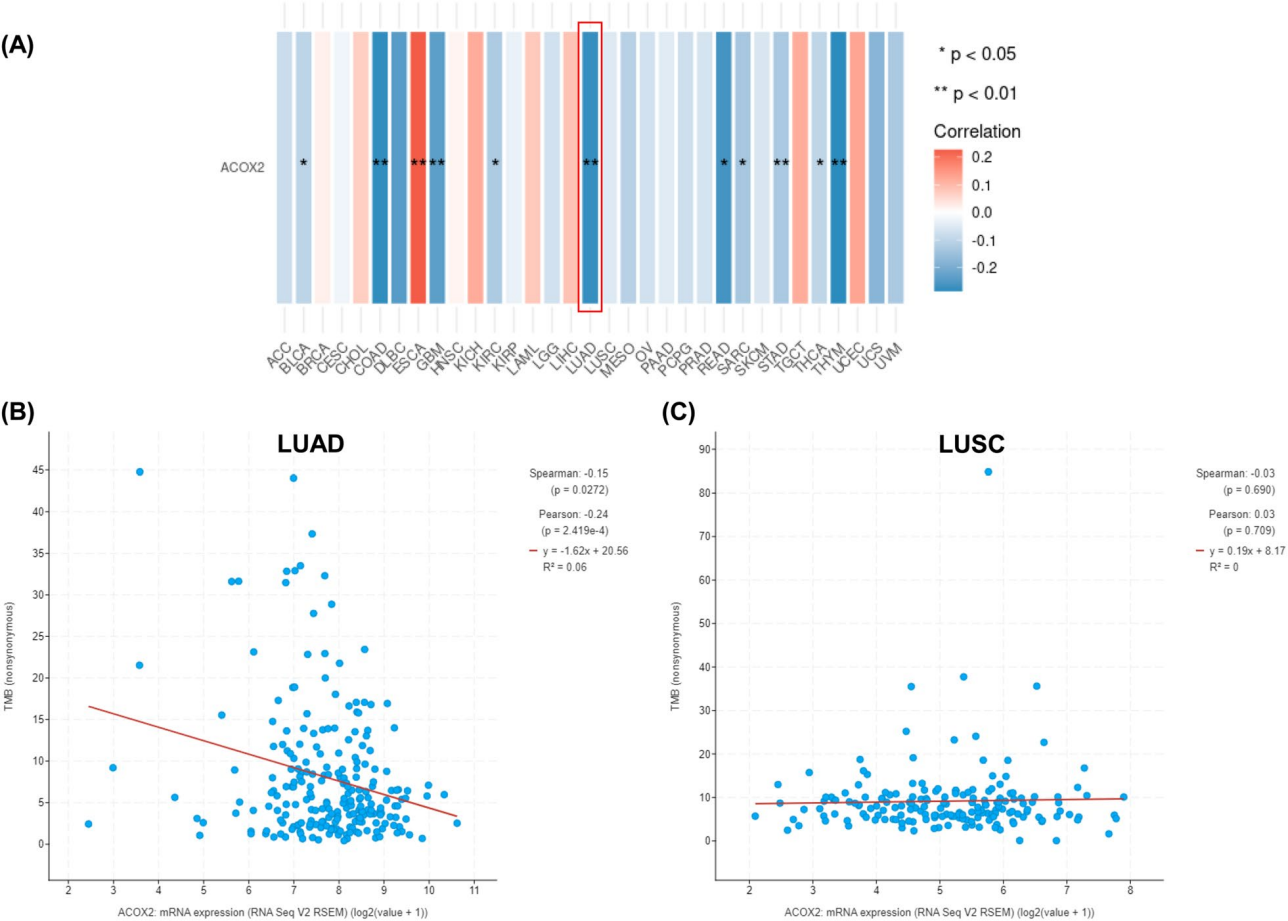
Associations between TMB and ACOX2 expression in NSCLC. The associations between TMB and ACOX2 expression were assessed in the TCGA datasets as follows: **A** XenaShiny analysis of the correlation between ACOX2 expression and TMB across all TCGA datasets; **B** cBioPortal analysis for ACOX2 expression and TMB in the TCGA-LUAD dataset; and **C** cBioPortal analysis for ACOX2 expression and TMB in the TCGA-LUSC dataset

Whilst this represents a proxy for assessing the effect of ACOX2 expression on TMB, we subsequently reassessed the TCGA datasets for whether ACOX2 expression is associated with TMB using functions in UCSCXenaShiny [60] and also through by querying cBioPortal [41, 42]. As shown in Fig. 7A TMB is significantly associated with ACOX2 expression in LUAD, as reflected in the cBioPortal data (Fig. 7B). In contrast, TMB has no association with ACOX2 expression for LUSC (Fig. 7C). It must be noted however, that the cBioPortal does not have TMB data on all the patients, and the correlations observed reflect only a subset of patients. The fact that TMB may not have any influence on ACOX2 expression in LUSC may reflect either the loss of expression of ACOX2 expression by DNA CpG methylation in LUSC (Fig. 5), or a limitation in the numbers of samples available in the TCGA-LUSC dataset.

Taken together, these results suggest that TMB may be associated with ACOX2 expression particularly in LUAD.

### ACOX2 and anti-cancer drug sensitivity in NSCLC

To examine any associations between ACOX2 expression and drug sensitivity, we examined the effects of ACOX2 expression against the pharmacological profiles for 24 anticancer drugs from the CCLE database [50]. Using parameters previously defined for IC50 for drug sensitive versus drug-resistant NSCLC [51], we identified three drugs for which lower ACOX2 mRNA expression was associated with resistance to 17-AAG (r = − 0.2399, p = 0.0344) a HSP90 inhibitor, PD-0325901 (r = -0.3472, p = 0.0018) a MEK inhibitor, and Crizotinib (r = − 0.2307, p = 0.0422) a c-MET/ALK inhibitor as shown in Fig. 6. A separate analysis using the DepMap PRISM repurposing Primary Screen [52] confirmed the potential to target MEK as it identified Tramatenib (GSK1120212) a MEK1/2 inhibitor FDA approved for the treatment of BRAF-mutated advanced NSCLC [61], and currently in a Phase I trial for KRAS mutated NSCLC [62].

## Discussion

Metabolic pathway reprogramming is now considered to be a hallmark of cancer [63], and is an evolving area of therapeutic interest. Peroxisomes are key metabolic organelles [9], with key emerging roles in various cancers [9, 12]. In this manuscript we identified the family of acyl-CoA oxidases as being significantly dysregulated in NSCLC. In particular, we demonstrate that ACOX2 is significantly downregulated in NSCLC in both LUAD and LUSC (Fig. 1A–C). Re-analysis of existing TCGA datasets confirms this significantly decreased expression in larger well established datasets for NSCLC (Fig. 1D), and further show that the level of ACOX2 protein is significantly altered in the CTPAC dataset for NSCLC LUAD (Fig. 1E). However, whilst high mRNA expression of ACOX2 was found to be a potential prognostic biomarker for overall survival, in contrast expression of ACOX2 protein was not found to have any overall prognostic value (Fig. 2). Surprisingly, ACOX2 mRNA was not found to have any association with PFS (Additional file 5: Figure S4), whereas all other associated genes examined demonstrated associations with PFS benefit (Additional file 1: Table S5). PFS is often used as a surrogate measure of clinical benefit particularly for clinical trial drug approvals [64], but crossover and post-progression treatments may bias the relationship between surrogate endpoints such as PFS and OS [65], and discrepancies between PFS and OS often arise [66]. Nevertheless, when clinical trials are biomarker driven, strong evidence links PFS to response rate and survival benefit [67, 68].

Loss of ACOX2 expression due to mutation has been shown to have a functional role in cardiac cancer [69], with associated metabolic defects [70]. Building on from this we identified a series of additional peroxisomal genes which were also altered in NSCLC (Additional file 3: Figure S2, Additional file 1: Table S2). During the preparation of this manuscript, a publication emerged that identified that other key peroxisomal genes such as ACAA1were dysregulated in NSCLC [25, 59], and our analyses confirm/validate the observations from these papers (Additional file 1: Tables S2 and S3). Additional analyses indicate that the altered expression of ACOX2 (and other acyl-CoA oxidases) has significant prognostic value at least at the mRNA level predominantly in LUAD (Additional file 4: Figure S3). Other peroxisomal genes which showed prognostic value were also predominantly associated with the LUAD histological subtype (Additional file 4: Figure S3; Additional file 1: Tables S3 and S4).

Epigenetics plays a role in the dysregulation of many genes in cancer [71]. One of the best established mechanisms by which this occurs is via DNA CpG methylation at the promoter regions of genes [72]. Analysis of existing methylation data in the TCGA datasets demonstrates that altered methylation does occur at the promoter of ACOX2, and in particular hypermethylation occurs predominantly in the ACOX2 promoter in LUSC samples (Fig. 6). Given that the expression of ACOX2 is reduced in our patient LUSC samples (Fig. 1C) and confirmed as dramatically reduced in the TCGA LUSC dataset compared to LUAD (Fig. 1D), this may indicate that aberrant DNA methylation plays more important roles in the downregulation of ACOX2 in the squamous cell subtype. This is reflected in the DNA methylation at particular promoter specific methylation probes which are predominantly significant in the LUSC samples but not in LUAD (Additional file 1: Table S5) for example cg16587010. Increased promoter methylation may be responsible for the loss of ACOX2 expression in LUSC, but it does not appear to be a factor in the loss of ACOX2 expression in LUAD. As such it would appear that multiple elements may play a role in the dysregulation of ACOX2 in NSCLC. In this regard, mutation of certain lung cancer “driver” genes such as TP53, KRAS and CDKN2A was also associated with altered expression of ACOX2 in both LUAD and LUSC (Table 2) suggesting that cohorts of patients with such mutations could have altered peroxisomal pathways that may play a role in tumorigenesis. Other additional genes were also identified which when mutated correlated with altered ACOX2 expression. Examples of these such as FAT2 and LRRC7 have known roles in various cancers including lung cancer [73–78].

One emerging area in cancer where the peroxisomal pathway plays important roles is in the regulation of cellular immune responses [12]. Indeed, similar observations for immune infiltrating scores for LUAD and LUSC have recently been identified which have prognostic value [79–81]. In this manuscript we linked dysregulated expression of ACOX2 in NSCLC to altered infiltration of immune cells into the tumour environment. In this regard, in LUAD a negative correlation between ACOX2 expression and CD8 + T cell infiltration (Table 4a) occurs, whereas in LUSC a positive correlation between ACOX2 expression and CD8 + T cell infiltration is observed (Table 4a). As such it is possible that this observation may have potential implications for patient management for checkpoint inhibitors given that CD8 + tumour infiltrating lymphocytes are associated with better outcome in NSCLC [82, 83]. Moreover, in colon adenocarcinomas, reduced peroxisomal number and associated enzymatic activities occurs, and most recently an at-risk subpopulation has been identified where a low peroxisome pathway score is associated with a worse clinical outcome and high immune cell infiltration in CRC patients [84], and suggesting that peroxisomal genes such as ACOX2 could potentially be used in a similar fashion to explore for further associations with respect to utility in a predictive score for checkpoint inhibitor stratification or outcome in NSCLC.

It has also been suggested that peroxisomes play a role in the Warburg effect [85], whereby cancer cells utilize glycolysis as their predominant energy source. Thus competition for nutrients between cancer cells and immune cells is proposed to extend the function of the Warburg effect to a cell-extrinsic advantage depleting extracellular glucose in the tumour microenvironment, and thus rendering tumour-infiltrating T cells dysfunctional [59, 86]. Given the altered expression of a significant number of peroxisomal associated genes identified in this and other studies [25, 59], and the demonstration that altered cholesterol metabolism can affect the anti-tumour response of CD8( +) T cells [87], the data presented here further links dysregulation of peroxisome function with effects on T cell infiltration and activity in NSCLC.

It has been suggested that it may be possible to target peroxisomes by inducing “pexophagy” or the autophagocytic degradation of peroxisomes [12]. However, no agents have currently been developed that specifically target the peroxisome [12]. It may however be possible to utilise anti-lipolytic agents, or drugs such as small molecule inhibitors of specific peroxisome proteins could alternatively be targeted [12].

**Fig. 8:**
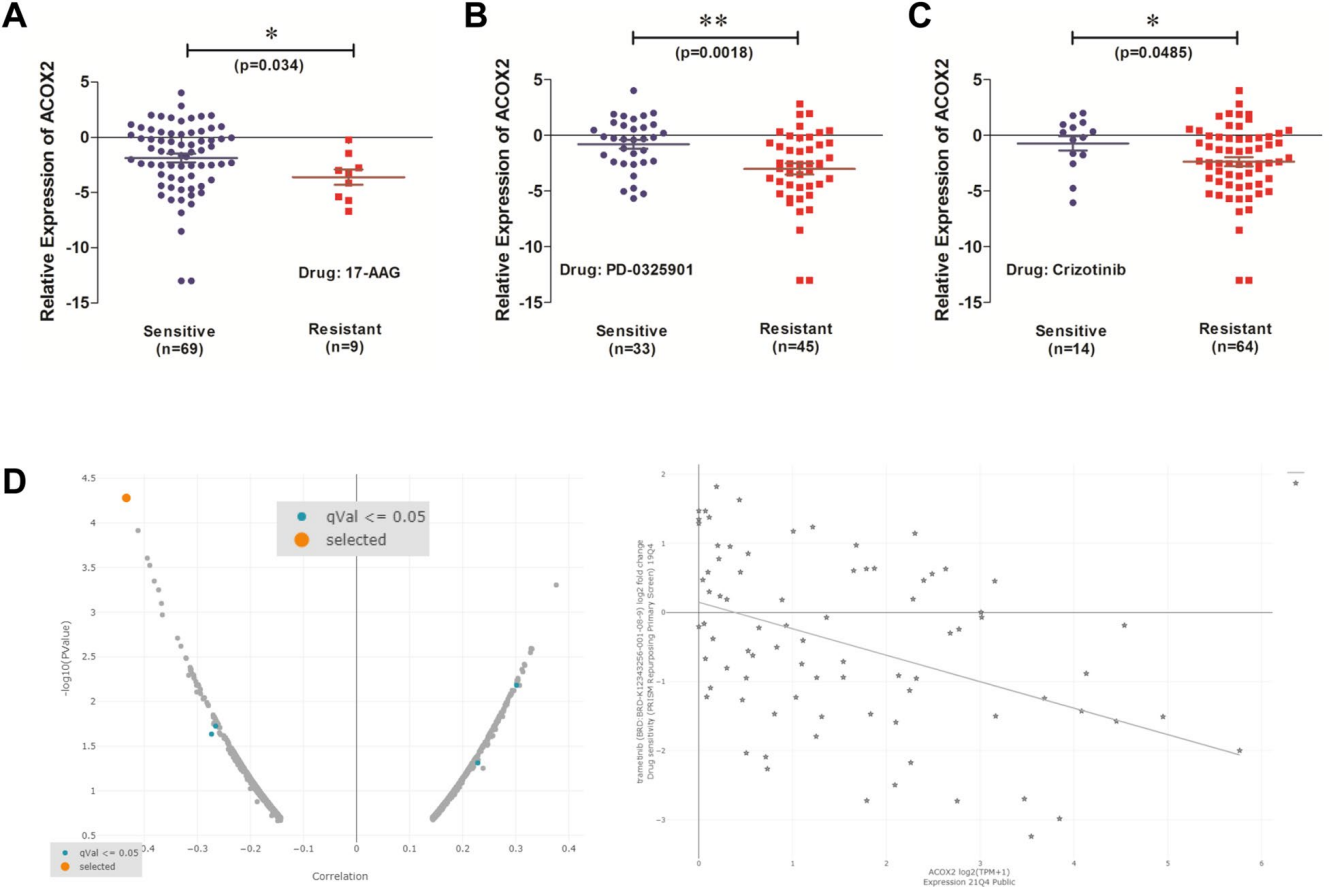
Associations between ACOX2 expression in NSCLC cell lines and differential anti-cancer drug sensitivity. Analysis of the CCLE database [50] demonstrated that lower ACOX2 mRNA expression differences were found to be associated with resistance to **A** Tanespimycin (HSP90 inhibitor), **B** PD-0235901 (MEK inhibitor) and **C** Crizotinib (c-MET/ALK inhibitor). A non-parametric Mann–Whitney test was used to assess for significance with p < 0.05 considered to be the threshold for significant. **D** Separate analysis of the DepMAp PRISM repurposing Primary Screen [52] also identified trametinib (GSK1120212) a separate MEK1/2 inhibitor as a potential candidate drug linked with ACOX2 expression

However, since the vast majority of patients with NSCLC display decreased ACOX2 at both the mRNA and protein level (Fig. 1), it may not be possible or clinically relevant to specifically target ACOX2 for the treatment of NSCLC patients. However, it may be possible to identify subsets of patients that may respond to other existing targeted therapies based on ACOX2 expression. In this regard, from our analysis increased sensitivity to established agents such as Tanespimycin (HSP90—Fig. 8A) and Crizotinib (c-MET/ALK—Fig. 8C) is associated with higher levels of ACOX2 expression. Moreover, MEK was identified as a candidate drug target associated with ACOX2 expression (Fig. 8B, D). MEK inhibitors are currently FDA approved for BRAF mutated advanced NSCLC [61], and are under investigation as a therapeutic option for KRAS mutated NSCLC [62]. As such it would be very interesting to compare levels of ACOX2 in a larger cohort of patients with KRAS mutations against a similar number of NSCLC wild-type for KRAS to determine if there may be any functional or biological link to sensitivity to MEK inhibitors. Indeed, a cohort of NSCLC patients with both RASA1 and NF-1 mutations has been shown to be uniquely sensitive to MEK inhibitors [78, 88], and as our analysis found that mutated RASA1 results in increased ACOX2 (Fig. 4) this lends further support to the possibility that cancers overexpressing ACOX2 may also be sensitive to MEK inhibitors. Despite the overall downregulation of ACOX2 protein in NSCLC (Fig. 1F, G), there is a subset of patients which demonstrate medium to high expression of this gene. Given the strong links we have identified between ACOX2 and mutated KRAS in both LUAD and LUSC (Table 2), it may be possible to expand the treatment options for MEK inhibitors to include those patients with high ACOX2 expression. As such it may therefore be possible to stratify patients into treatment or develop clinical trials using these agents based on expression levels of this gene, but additional investigations will be required to fully establish these possibilities.

Nevertheless, it may be also possible to potentially restore levels of ACOX2 in patients where decreased ACOX2 is expressed through (PPAR)-α/γ dual activators such as SN158, which was shown to significantly upregulate ACOX2 mRNA [89], although such drugs have yet to enter the clinic due to serious side effects. Nevertheless, both in vitro and in vivo studies suggest that PPAR based agonists such as pioglitazone may have potential for treating NSCLC [90–92], however some degree of caution is indicated as other studies of such agonists suggest that when activated in myeloid cells of the tumour microenvironment, this may aggravate and promote lung cancer progression [2].

Overall, the results presented in this study raise interesting possibilities with respect to the potential use of ACOX2 in the diagnosis/prognosis of patients with NSCLC. Moreover, the results suggest that ACOX2 levels could potentially be useful to stratify patients into subsets that may respond to checkpoint inhibitor therapy. In addition, expression of ACOX2 can also potentially be used to stratify patients that may respond to various drugs including Tanespimycin, Crizotinib and MEK inhibitors. However, further studies will be required to elucidate the exact roles of these genes in the pathogenesis of LUAD and LUSC respectively, and also to further delineate the potential for use of ACOX2 expression to stratify or link patient responses to MEK inhibitors and potential subsets of patients with actionable mutations such as BRAF, KRAS, RASA1 and NF-1.

## Supplementary Information

**Additional file 1**. Analysis of TCGA-LUAD and –LUSC datasets for alterations in acyl-CoA peroxidases at the mRNA and protein level.

**Additional file 2**: **Figure S1**. Protein-Protein Interaction Networks associated with acyl-CoA oxidases. Functional enrichment analysis of acyl-CoA oxidases was carried out on the STRING database [34] to identify Protein-Protein Interaction Networks. The results were imported into Cytoscape [34] and first neighbours highlighted. The resulting first-neighbours are shown.

**Additional file 3**: **Figure S2**. IHC of ACOX2. Representative examples of ACOX2 protein expression in NSCLC. (A) Negative staining (B) Positive Staining . Images were taken at 40x.

**Additional file 4**: **Figure S3**. Prognostic value of acyl-CoA oxidases and other peroxisomal genes in NSCLC. The prognostic effects of ACOX2, other acyl-CoA oxidases and key other peroxisomal associated genes were assessed for overall survival (OS) using KM-Plotter [38]. Analyses were conducted on the gene chip datasets for LUAD. Higher expression of the mRNA for (A) ACOX1, (B) ACOX2, (C) ACOX3, (D) ACOXL, (E) AGPS and (F) CAT was found to be associated with significantly better OS in LUAD, with p<0.05 considered to be significant.

**Additional file 5**: **Figure S4**. Prognostic value of acyl-CoA oxidases mRNA expression in NSCLC as assessed by Progression Free Survival. Progression free survival (PFS) is defined First Progression (FP) and KM-Plot was used to analyse ACOX1-3 across the NSCLC. The results are presented as follows: (A) ACOX1 – all histologies; (B) ACOX1 – LUAD; (C) ACOX1 – LUSC; (D) ACOX2 – all histologies; (E) ACOX2 – LUAD; (F) ACOX2 – LUSC; (G) ACOX3 – all histologies; (H) ACOX3 – LUAD; (I) ACOX3 – LUSC.

**Additional file 6**: **Figure S5**. Meta-analysis of ACOX2 expression in multiple NSCLC datasets. A meta-analysis was conducted on LCE [33] for a large number of gene expression datasets generating forest plots summarizing (A) tumour - normal standardized mean difference for tumour vs normal meta-analysis and (B) hazard ratios for OS meta-analysis for all NSCLC datasets.

**Additional file 7**: **Figure S6**. Meta-analysis of ACOX2 expression in multiple NSCLC datasets. Meta-analysis conducted on LCE [32] generating forest plots summarizing hazard ratios for OS in (A) LUAD and (B) LUSC specific datasets.

## Data Availability

The data that support the findings presented in this study are available for interrogation at the following online resources: TIMER: https://cistrome.shinyapps.io/timer/. TIMER2.0: http://timer.cistrome.org/. GEPIA2.0: http://gepia2.cancer-pku.cn/#index. LCE: http://lce.biohpc.swmed.edu/lungcancer/. UALCAN: http://ualcan.path.uab.edu/index.html. Oncolnc: http://oncolnc.org. KM-PLOT: https://kmplot.com/analysis/index.php?p=background. cBioPortal: https://www.cbioportal.org/. TCGA WANDERER: http://maplab.imppc.org/wanderer/#. MEXPRESS: https://mexpress.be/index.html. CCLE: https://portals.broadinstitute.org/ccle. MuTarget: https://www.mutarget.com/. DepMap: https://depmap.org/portal/. cProSite: https://cprosite.ccr.cancer.gov/#/. ESTIMATE: https://bioinformatics.mdanderson.org/estimate/index.html.
