# Supplementary material for: Altered expression of ACOX2 in non-small cell lung cancer
Source: BMC Pulm Med. 2022 Aug 23;22:321. doi: 10.1186/s12890-022-02115-7 (PMC9396774; doi:10.1186/s12890-022-02115-7)
Supplement: Supplementary file 1 — Additional file 1. Analysis of TCGA-LUAD and –LUSC datasets for alterations in acyl-CoA peroxidases at the mRNA and protein level. [file 12890_2022_2115_MOESM1_ESM.pdf]

**Supplementary Table 1: Analysis of TCGA-LUAD and –LUSC datasets for alterations in acyl-CoA peroxidases at the mRNA and protein level**

**mRNA**

| **Gene** | **TCGA-LUAD** | **TCGA-LUSC** |
|---|---|---|
| **ACOX1** | Not Significant | down **($p=6.4x10^{-7}$)** |
| **ACOX2** | down **($p= 1.7 \times 10^{-4}$)** | down **($p= 1.2 \times 10^{-68}$)** |
| **ACOX3** | Not Significant | down **($p= 4.8 \times 10^{-16}$)** |

**Protein**

| | **CPTAC-LUAD** | **CPTAC-LUSC** |
|---|---|---|
| **ACOX1** | 0.6228164 | N/A |
| **ACOX2** | **$5.76 x10^{-05}$** | N/A |
| **ACOX3** | 0.139 | N/A |

**Protein**

| | **cProSite-LUAD** | | **cProSite-LUSC** | |
|---|---|---|---|---|
| | P Value (Paired) | P Value (Unpaired) | P Value (Paired) | P Value (Unpaired) |
| **ACOX1** | 0.1829 | 0.2392 | 0.1604 | 0.1577 |
| **ACOX2** | **0.0001** | **< 0.0001** | **< 0.0001** | **< 0.0001** |
| **ACOX3** | **0.0363** | 0.0788 | **< 0.0001** | **< 0.0001** |

mRNA analysis was conducted using LCE (Comparative Analysis) [1] and the parameter chosen was normal lung tissue versus tumor tissue for the TCGA LUAD [2] and TCGA LUSC [3] datasets. Protein analysis was conducted on CPTAC data set using UALCAN [4], or alternatively using cProSite [5].

**Supplementary Table 2: Systematic Analysis results for acyl-CoA oxidases and associated first neighbours in NSCLC as determined using Lung Cancer Explorer.**

**Meta-analysis of Standardized Mean Difference of Tumor-Normal gene expression**

| Entrez ID | Symbol | SMD | SMD lower | SMD upper | pv | p.adj | Tumor-Normal Standardized Expression Difference |
|---|---|---|---|---|---|---|---|
| 51 | ACOX1 | 0.46 | 0.2 | 0.72 | 0.00046 | **0.0011** | Adenocarcinoma |
| | | -0.2 | -0.45 | 0.05 | 0.11 | 0.16 | Squamous Cell Carcinoma |
| 8309 | ACOX2 | 0.06 | -0.18 | 0.3 | 0.6 | 0.67 | Adenocarcinoma |
| | | -1.67 | -2.69 | -0.64 | 0.0014 | **0.0031** | Squamous Cell Carcinoma |
| 8310 | ACOX3 | -0.2 | -0.53 | 0.12 | 0.22 | 0.29 | Adenocarcinoma |
| | | -0.65 | -1.01 | -0.28 | 0.00046 | **0.0011** | Squamous Cell Carcinoma |
| 55289 | ACOXL | -1.26 | -2.07 | -0.44 | 0.0025 | **0.0052** | Adenocarcinoma |
| | | -2.42 | -3.74 | -1.1 | 0.00033 | **0.00083** | Squamous Cell Carcinoma |
| 30 | ACAA1 | -0.65 | -0.92 | -0.38 | 1. 6e-06 | **5.6e-06** | Adenocarcinoma |
| | | -1.76 | -2.28 | -1.23 | 4.7e-11 | **3.9e-10** | Squamous Cell Carcinoma |
| 34 | ACADM | -0.23 | -0.6 | 0.14 | 0.22 | 0.29 | Adenocarcinoma |
| | | -0.85 | -1.17 | -0.53 | 2.7e-07 | **1.2e-06** | Squamous Cell Carcinoma |
| 122970 | ACOT4 | -0.15 | -0.37 | 0.07 | 0.18 | 0.24 | Adenocarcinoma |
| | | -1.31 | -1.61 | -1.01 | 1.9e-17 | **3.4e-16** | Squamous Cell Carcinoma |
| 2180 | ACSL1 | -1.08 | -1.3 | -0.85 | 4.9e-21 | **8e-20** | Adenocarcinoma |
| | | -1.51 | -2.22 | -0.8 | 3.1e-05 | **9.5e-05** | Squamous Cell Carcinoma |
| 51703 | ACSL5 | 0.25 | -0.02 | 0.52 | 0.075 | 0.11 | Adenocarcinoma |
| | | -2.28 | -2.93 | -1.64 | 4.8e-12 | **4.6e-11** | Squamous Cell Carcinoma |
| 8540 | AGPS | 0.04 | -0.5 | 0.58 | 0.89 | 0.91 | Adenocarcinoma |
| | | 0.3 | -0.46 | 1.05 | 0.44 | 0.52 | Squamous Cell Carcinoma |
| 23600 | AMACR | 0.99 | 0.52 | 1.47 | 4.2e-05 | **0.00012** | Adenocarcinoma |
| | | 0.63 | 0.08 | 1.17 | 0.024 | 0.04 | Squamous Cell Carcinoma |
| 847 | CAT | 2.43 | -2.67 | -2.19 | 6.1e-87 | **5.1e-84** | Adenocarcinoma |
| | | 2.86 | -3.64 | -2.09 | 4.5e-13 | **4.8e-12** | Squamous Cell Carcinoma |
| 1384 | CRAT | 0.38 | 0.16 | 0.6 | 0.00082 | **0.0019** | Adenocarcinoma |
| | | .12 | -0.15 | 0.4 | 0.38 | 0.46 | Squamous Cell Carcinoma |
| 1610 | DAO | -0.18 | -0.78 | 0.42 | 0.56 | 0.64 | Adenocarcinoma |

| | | | | | | | |
|---|---|---|---|---|---|---|---|
| | | -0.35 | -1.18 | 0.47 | 0.4 | 0.48 | Squamous Cell Carcinoma |
| 26063 | DECR2 | 1.44 | 1.19 | 1.69 | 1.7e-29 | **5.3e-28** | Adenocarcinoma |
| | | 0.47 | 0.24 | 0.71 | 6.9e-05 | **2e-04** | Squamous Cell Carcinoma |
| 1891 | ECH1 | 0.08 | -0.09 | 0.25 | 0.37 | 0.46 | Adenocarcinoma |
| | | -0.02 | -0.33 | 0.29 | 0.9 | 0.93 | Squamous Cell Carcinoma |
| 1962 | EHHADH | 0.16 | -0.01 | 0.34 | 0.063 | 0.096 | Adenocarcinoma |
| | | 1.12 | 0.46 | 1.77 | 0.00081 | **0.0019** | Squamous Cell Carcinoma |
| 8443 | GNPAT | 0.81 | 0.52 | 1.1 | 6.3e-08 | **2.7e-07** | Adenocarcinoma |
| | | 0.59 | 0.25 | 0.93 | 0.00062 | **0.0015** | Squamous Cell Carcinoma |
| 3033 | HADH | -0.1 | -0.31 | 0.11 | 0.35 | 0.43 | Adenocarcinoma |
| | | -0.31 | -0.65 | 0.02 | 0.068 | 0.1 | Squamous Cell Carcinoma |
| 3032 | HADHB | 0.17 | -0.34 | 0 | 0.046 | 0.073 | Adenocarcinoma |
| | | 0.26 | -0.07 | 0.59 | 0.13 | 0.18 | Squamous Cell Carcinoma |
| 54363 | HAO1 | 0.04 | -0.2 | 0.28 | 0.73 | 0.78 | Adenocarcinoma |
| | | -0.08 | -0.27 | 0.1 | 0.37 | 0.45 | Squamous Cell Carcinoma |
| 51179 | HAO2 | 0.24 | -0.01 | 0.48 | 0.062 | 0.095 | Adenocarcinoma |
| | | 0.24 | -0.06 | 0.54 | 0.12 | 0.17 | Squamous Cell Carcinoma |
| 3295 | HSD17B4 | -1.12 | -1.85 | -0.39 | 0.0025 | **0.005** | Adenocarcinoma |
| | | -1.88 | -2.99 | -0.78 | 0.00081 | **0.0019** | Squamous Cell Carcinoma |
| 55825 | PECR | -0.53 | -0.85 | -0.21 | 0.0013 | **0.0029** | Adenocarcinoma |
| | | -1.6 | -1.9 | -1.3 | 1.2e-25 | **4.3e-24** | Squamous Cell Carcinoma |
| 5830 | PEX5 | -0.22 | -0.59 | 0.14 | 0.23 | 0.3 | Adenocarcinoma |
| | | 0.32 | 0.15 | 0.49 | 0.00031 | **0.00078** | Squamous Cell Carcinoma |
| 5195 | PEX14 | -0.5 | -0.91 | -0.08 | 0.018 | **0.032** | Adenocarcinoma |
| | | -0.41 | -0.85 | 0.03 | 0.066 | 0.1 | Squamous Cell Carcinoma |
| 6342 | SCP2 | -0.09 | -0.29 | 0.11 | 0.38 | 0.46 | Adenocarcinoma |
| | | -0.91 | -1.64 | -0.19 | 0.013 | **0.024** | Squamous Cell Carcinoma |

Entrez ID -NCBI designated Gene ID; Symbol – Gene Symbol; SMD – tumor-normal standardized mean difference; SMD.lower – lower bound of 95% confidence interval for SMD; SMD.upper – upper bound of 95% confidence interval for SMD; pv – p-value; p.adj – multiple comparison adjusted p-value by Benjamini Hochberg procedures

**Supplementary Table 3: Systematic meta-analysis of Survival Associations for acyl-CoA oxidases and associated first neighbours in NSCLC as determined using Lung Cancer Explorer based on Cox Proportional Hazards Model.**

| Entrez ID | Symbol | HR | Z | pv | p.adj | Tumor-Normal Standardized Expression Difference |
|---|---|---|---|---|---|---|
| 51 | ACOX1 | 1.08 | 2.17 | 0.03 | 0.1 | Adenocarcinoma |
| | | 1.06 | 1.15 | 0.25 | 0.82 | Squamous Cell Carcinoma |
| 8309 | ACOX2 | 0.86 | -4.11 | 3.9e-05 | **0.00049** | Adenocarcinoma |
| | | 1.03 | 0.52 | 0.6 | 0.94 | Squamous Cell Carcinoma |
| 8310 | ACOX3 | 0.99 | -0.12 | 0.9 | 0.95 | Adenocarcinoma |
| | | 1 | -0.02 | 0.99 | 1 | Squamous Cell Carcinoma |
| 55289 | ACOXL | 0.88 | -2.81 | 0.005 | **0.025** | Adenocarcinoma |
| | | 1.09 | 0.79 | 0.43 | 0.9 | Squamous Cell Carcinoma |
| 30 | ACAA1 | 0.87 | -4.21 | 2.5e-05 | **0.00034** | Adenocarcinoma |
| | | 0.99 | -0.29 | 0.77 | 0.98 | Squamous Cell Carcinoma |
| 34 | ACADM | 0.9 | -1.88 | 0.06 | 0.17 | Adenocarcinoma |
| | | 1.06 | 1.19 | 0.23 | 0.81 | Squamous Cell Carcinoma |
| 122970 | ACOT4 | 1.1 | 2.56 | 0.01 | **0.044** | Adenocarcinoma |
| | | 0.95 | -0.83 | 0.4 | 0.89 | Squamous Cell Carcinoma |
| 2180 | ACSL1 | 0.97 | -0.67 | 0.5 | 0.69 | Adenocarcinoma |
| | | 0.97 | -0.41 | 0.68 | 0.96 | Squamous Cell Carcinoma |
| 51703 | ACSL5 | 0.85 | -4.96 | 7.1e-07 | **1.7e-05** | Adenocarcinoma |
| | | 1.11 | 1.92 | 0.055 | 0.57 | Squamous Cell Carcinoma |
| 8540 | AGPS | 1.23 | 5.02 | 5.1e-07 | **1.3e-05** | Adenocarcinoma |
| | | 1.11 | 1.87 | 0.061 | 0.59 | Squamous Cell Carcinoma |
| 23600 | AMACR | 0.96 | -0.82 | 0.41 | 0.61 | Adenocarcinoma |
| | | 1.02 | 0.4 | 0.69 | 0.96 | Squamous Cell Carcinoma |
| 847 | CAT | 0.79 | -6.82 | 8.8e-12 | **1e-09** | Adenocarcinoma |
| | | 1.01 | 0.08 | 0.94 | 0.99 | Squamous Cell Carcinoma |
| 1384 | CRAT | 0.98 | -0.63 | 0.53 | 0.71 | Adenocarcinoma |
| | | 1.04 | 0.99 | 0.32 | 0.86 | Squamous Cell Carcinoma |
| 1610 | DAO | 0.96 | -0.91 | 0.36 | 0.57 | Adenocarcinoma |

| | | | | | | |
|---|---|---|---|---|---|---|
| | | 0.99 | -0.22 | 0.83 | 0.98 | Squamous Cell Carcinoma |
| 26063 | DECR2 | 0.99 | -0.31 | 0.75 | 0.87 | Adenocarcinoma |
| | | 1.06 | 1.17 | 0.24 | 0.81 | Squamous Cell Carcinoma |
| 1891 | ECH1 | 0.98 | -0.42 | 0.67 | 0.82 | Adenocarcinoma |
| | | 1.01 | 0.21 | 0.83 | 0.98 | Squamous Cell Carcinoma |
| 1962 | EHHADH | 1.05 | 1.04 | 0.3 | 0.5 | Adenocarcinoma |
| | | 0.92 | -2.02 | 0.043 | 0.54 | Squamous Cell Carcinoma |
| 8443 | GNPAT | 0.99 | -0.27 | 0.79 | 0.89 | Adenocarcinoma |
| | | 1.05 | 1.07 | 0.28 | 0.84 | Squamous Cell Carcinoma |
| 3033 | HADH | 0.86 | -3.65 | 0.00026 | **0.0023** | Adenocarcinoma |
| | | 1 | -0.01 | 0.99 | 1 | Squamous Cell Carcinoma |
| 3032 | HADHB | 0.98 | -0.59 | 0.55 | 0.73 | Adenocarcinoma |
| | | 0.97 | -0.43 | 0.67 | 0.96 | Squamous Cell Carcinoma |
| 54363 | HAO1 | 1.02 | 0.58 | 0.56 | 0.74 | Adenocarcinoma |
| | | 1.02 | 0.27 | 0.78 | 0.98 | Squamous Cell Carcinoma |
| 51179 | HAO2 | 1.02 | 0.34 | 0.74 | 0.86 | Adenocarcinoma |
| | | 1.08 | 1.78 | 0.075 | 0.62 | Squamous Cell Carcinoma |
| 3295 | HSD17B4 | 0.85 | -3.79 | 0.00015 | **0.0015** | Adenocarcinoma |
| | | 0.97 | -0.42 | 0.67 | 0.96 | Squamous Cell Carcinoma |
| 55825 | PECR | 0.95 | -1.41 | 0.16 | 0.33 | Adenocarcinoma |
| | | 1.05 | 1.06 | 0.29 | 0.84 | Squamous Cell Carcinoma |
| 5830 | PEX5 | 0.98 | -0.63 | 0.53 | 0.71 | Adenocarcinoma |
| | | 1.03 | 0.59 | 0.56 | 0.93 | Squamous Cell Carcinoma |
| 5195 | PEX14 | 0.99 | -0.2 | 0.84 | 0.92 | Adenocarcinoma |
| | | 1.07 | 1.57 | 0.12 | 0.7 | Squamous Cell Carcinoma |
| 6342 | SCP2 | 0.91 | -3.18 | 0.0015 | **0.0094** | Adenocarcinoma |
| | | 1.03 | 0.61 | 0.54 | 0.93 | Squamous Cell Carcinoma |

Entrez ID -NCBI designated Gene ID; Symbol – Gene Symbol; HR – Hazard Ratio; Z – Z score from survival association meta-analysis; pv – p-value; p.adj – multiple comparison adjusted p-value by Benjamini Hochberg procedures

**Supplementary Table 4: Comparison of Kaplan-Meier analyses using different platforms for prognostic value.**

| Symbol | KM-Plot (microarray) | KM-Plot (RNA-Seq) | LCE† | OncoLnc * | UALCAN ** | Tumor-Normal Standardized Expression Difference |
|---|---|---|---|---|---|---|
| ACOX1 | **p=0.00064** | p=0.11 | p=0.19 | p=0.887 | **p=0.041** | Adenocarcinoma |
| | p=0.057 | p=0.11 | p=1.0 | p=0.857 | p=0.44 | Squamous Cell Carcinoma |
| ACOX2 | **p=0.0004** | p=0.0563 | p=0.9 | p=0.202 | p=0.27 | Adenocarcinoma |
| | p=0.9 | p= 0.0558 | p=0.14 | p=0.407 | p=0.38 | Squamous Cell Carcinoma |
| ACOX3 | **p= $9.3ex10^{-6}$** | p=0.1125 | p=0.56 | p=0.494 | p=0.17 | Adenocarcinoma |
| | p=0.17 | **p= 0.0037** | p=0.16 | p=0.162 | p=0.78 | Squamous Cell Carcinoma |
| ACOXL | **p= $7.8ex10^{-5}$** | **p=0.0013** | **p=0.033** | **p=0.0118** | **p=0.0018** | Adenocarcinoma |
| | p= 0.8366 | **p= 0.0188** | **p=0.0078** | **p=0.0355** | p=0.52 | Squamous Cell Carcinoma |
| ACAA1 | **p=$1.0x10^{-5}$** | **p= 0.01** | p=0.059 | p= 0.0693 | p=0.061 | Adenocarcinoma |
| | p= 0.8984 | p= 0.0945 | p=0.51 | p=0.238 | **p=0.038** | Squamous Cell Carcinoma |
| ACADM | **p=$8.6x10^{-8}$** | p=0.2933 | p=0.97 | p=0.791 | p=0.73 | Adenocarcinoma |
| | p= 0.6318 | **p=0.0181** | p=0.1 | p= 0.0797 | p=0.72 | Squamous Cell Carcinoma |
| ACOT4 | **p=0.0012** | p=0.1013 | p=0.74 | p=0.501 | p=0.45 | Adenocarcinoma |
| | p=0.8737 | p=0.0988 | p=0.12 | p=0.855 | p=0.39 | Squamous Cell Carcinoma |
| ACSL1 | **p=$6.5x10^{-5}$** | **p=0.0149** | **p=0.043** | p= 0.944 | p=0.42 | Adenocarcinoma |
| | p=0.0676 | p= 0.3376 | p=0.63 | p=0.965 | p=0.94 | Squamous Cell Carcinoma |
| ACSL5 | **p=$7.8x10^{-11}$** | **p=0.0412** | p=1.0 | p=0.29 | p=0.32 | Adenocarcinoma |
| | p= 0.5559 | **p=0.0062** | p=0.12 | p= 0.0573 | p=0.085 | Squamous Cell Carcinoma |
| AGPS | **p=$4.4x10^{-6}$** | **p=$6.2x10^{-5}$** | **p=0.0079** | **p= 0.000456** | **p=0.0044** | Adenocarcinoma |
| | p= 0.2198 | p=0.292 | p=0.83 | p=0.346 | p=0.078 | Squamous Cell Carcinoma |
| AMACR | p=0.0503 | **p=0.0229** | p=0.6 | p=0.062 | p=0.079 | Adenocarcinoma |

| | | | | | | |
|---|---|---|---|---|---|---|
| | p=0.1785 | p=0.2022 | p=0.17 | p=0.718 | p=0.73 | Squamous Cell Carcinoma |
| CAT | **$p=9.4x10^{-14}$** | **p= 0.0012** | **p=0.034** | **p=0.0427** | p=0.084 | Adenocarcinoma |
| | p=0.0707 | p=0.0911 | p=0.2 | p=0.193 | p=0.81 | Squamous Cell Carcinoma |
| CRAT | p=0.1784 | **p=0.0349** | p=0.21 | p=0.122 | p=0.2 | Adenocarcinoma |
| | p=0.2327 | p=0.2766 | p=0.51 | p=0.651 | p=0.34 | Squamous Cell Carcinoma |
| DAO | **p=0.0031** (206878_at) | **p=0.0072** | p=0.19 | N/A | p=0.18 | Adenocarcinoma |
| | p=0.5905 (206878_at) | p=0.1582 | **p=0.0032** | N/A | p=0.22 | Squamous Cell Carcinoma |
| DECR2 | p=0.0704 | p=0.3494 | p=0.28 | p=0.689 | p=0.81 | Adenocarcinoma |
| | p=0.7191 | p=0.1682 | p=0.26 | p=0.996 | p=0.86 | Squamous Cell Carcinoma |
| ECH1 | **p=0.0003** | **p=0.0457** | p=0.46 | p=0.357 | p=0.51 | Adenocarcinoma |
| | **p=0.0342** | **p=0.0173** | p=0.59 | p=0.121 | p=0.5 | Squamous Cell Carcinoma |
| EHHADH | **p=0.014** | **p=0.0293** | p=0.79 | p=0.44 | **p=0.022** | Adenocarcinoma |
| | p=0.677 | **p=0.0228** | p=0.3 | p=0.313 | p=0.18 | Squamous Cell Carcinoma |
| GNPAT | **p=0.0191** | p=0.1069 | p=0.3 | p=0.236 | p=0.22 | Adenocarcinoma |
| | p=0.2291 | p=0.1231 | p=0.98 | p=0.879 | p=0.57 | Squamous Cell Carcinoma |
| HADH | **$p<1x10^{-16}$** | **p=0.0052** | p=0.09 | p=0.165 | p=0.39 | Adenocarcinoma |
| | p=0.4194 | p=0.1856 | p=0.96 | p=0.826 | p=0.63 | Squamous Cell Carcinoma |
| HADHB | **$p=9.4x10^{-7}$** | p=0.1667 | p=0.21 | p=0.968 | p=0.74 | Adenocarcinoma |
| | p=0.207 | p=0.1601 | p=0.15 | p=0.754 | p=0.42 | Squamous Cell Carcinoma |
| HAO1 | **$p=2.8x10^{-5}$** | **p=0.0104** | p=0.99 | N/A | N/A | Adenocarcinoma |
| | p=0.6622 | **p=0.0358** | **p=0.021** | N/A | N/A | Squamous Cell Carcinoma |
| HAO2 | **p=0.0146** | p=0.1516 | p=0.75 | N/A | p=0.77 | Adenocarcinoma |
| | p=0.3659 | **p=0.0389** | p=0.44 | N/A | p=0.24 | Squamous Cell Carcinoma |
| HSD17B4 | **$p=3.0x10^{-11}$** | **p=0.0139** | p=0.4 | p=0.37 | p=0.063 | Adenocarcinoma |

| | | | | | | |
|---|---|---|---|---|---|---|
| | p=0.9316 | **p=0.0097** | p=0.5 | p=0.199 | **p=0.032** | Squamous Cell Carcinoma |
| PECR | **p=0.00077** | p=0.095 | p=0.68 | p=0.984 | p=0.93 | Adenocarcinoma |
| | p=0.3655 | p=0.3043 | p=0.85 | p=0.929 | p=0.6 | Squamous Cell Carcinoma |
| PEX5 | p=0.6514 | **p=0.00018** | p=0.44 | p=0.416 | p=0.21 | Adenocarcinoma |
| | p=0.996 | p=0.34 | p=0.65 | p=0.402 | p=0.64 | Squamous Cell Carcinoma |
| PEX14 | **p=0.0208** | p=0.1011 | p=0.93 | p=0.129 | p=0.67 | Adenocarcinoma |
| | p=0.3825 | **p=0.0479** | p=0.65 | p=0.215 | p=0.32 | Squamous Cell Carcinoma |
| SCP2 | p=0.7007 (206546_at) | p=0.1314 | p=0.11 | p=0.36 | p=0.3 | Adenocarcinoma |
| | p=0.1842 (206546_at) | p=0.3137 | p=0.92 | p=0.816 | p=0.97 | Squamous Cell Carcinoma |

† - analysis utilized the TCGA LUAD [2] and TCGA LUSC [3] datasets

* - values assessed at 50:50 percentiles

** - values examined high vs low/medium expression (default)

Supplementary Table 5: PFS

| Gene | Histology | Hazard Ratio | logrank P value |
|---|---|---|---|
| ACOX1 | All | 0.49 (0.37 − 0.65) | 2.8e−07 |
| | Adenocarcinoma | 0.5 (0.36 − 0.7) | 3e−05 |
| | Squamous Cell Carcinoma | 0.51 (0.3 − 0.86) | 0.01 |
| ACOX2 | All | 1.03 (0.85 − 1.24) | 0.77 |
| | Adenocarcinoma | 0.84 (0.62 − 1.15) | 0.28 |
| | Squamous Cell Carcinoma | 0.8 (0.48 − 1.34) | 0.39 |
| ACOX3 | All | 0.71 (0.59 − 0.86) | 0.00049 |
| | Adenocarcinoma | 0.58 (0.43 − 0.8) | 0.00077 |
| | Squamous Cell Carcinoma | 0.83 (0.5 − 1.38) | 0.47 |
| ACOXL | All | 0.46 (0.35 − 0.62) | 4.5e−08 |
| | Adenocarcinoma | 0.42 (0.3 − 0.59) | 2.8e−07 |
| | Squamous Cell Carcinoma | 0.8 (0.48 − 1.34) | 0.4 |
| AGPS | All | 0.78 (0.59 − 1.02) | 0.067 |
| | Adenocarcinoma | 0.6 (0.43 − 0.83) | 0.0018 |
| | Squamous Cell Carcinoma | 1.77 (1.05 − 2.98) | 0.03 |
| CAT | All | 0.63 (0.52 − 0.76) | 1.6e−06 |
| | Adenocarcinoma | 0.53 (0.39 − 0.73) | 6.8e−05 |
| | Squamous Cell Carcinoma | 1.13 (0.68 − 1.89) | 0.64 |

**Supplementary Table 6. Mutated genes in LUAD that significantly correlate with altered ACOX2 expression**

| Mutation of | Mean expression (mutant) | Mean expression (wild) | Number of mutant | Number of wild | FC (mutant/wild) | Direction | p-value |
|---|---|---|---|---|---|---|---|
| TP53 | 307.23 | 476.67 | 241 | 267 | 1.56 | down | 6.64E-12 |
| FAT2 | 232.1 | 410.32 | 40 | 468 | 1.75 | down | 2.47E-06 |
| PTPRZ1 | 240.6 | 412.16 | 47 | 461 | 1.72 | down | 6.30E-06 |
| GUCY1A3 | 135.71 | 403.67 | 14 | 494 | 2.94 | down | 9.82E-06 |
| LRRC7 | 288.01 | 415.63 | 77 | 431 | 1.45 | down | 3.47E-05 |
| PDE3A | 210.04 | 405.52 | 24 | 484 | 1.92 | down | 3.87E-05 |
| MGAM | 246.95 | 409.74 | 42 | 466 | 1.67 | down | 3.93E-05 |
| CSMD2 | 285.1 | 414.05 | 70 | 438 | 1.45 | down | 4.25E-05 |
| LRIT1 | 131.92 | 402.68 | 12 | 496 | 3.03 | down | 5.32E-05 |
| FAM171B | 198.27 | 405.25 | 22 | 486 | 2.04 | down | 6.57E-05 |
| PKHD1L1 | 279.2 | 411.67 | 59 | 449 | 1.47 | down | 6.97E-05 |
| IRS1 | 168.75 | 403.68 | 16 | 492 | 2.38 | down | 7.24E-05 |
| IFT172 | 194.11 | 404.14 | 19 | 489 | 2.08 | down | 9.23E-05 |
| AHNAK2 | 282.56 | 414.46 | 70 | 438 | 1.47 | down | 9.43E-05 |
| NOVA1 | 180.11 | 404.22 | 18 | 490 | 2.22 | down | 1.06E-04 |
| SORCS1 | 271.08 | 413.05 | 60 | 448 | 1.52 | down | 1.15E-04 |
| PKHD1 | 285.63 | 411.95 | 63 | 445 | 1.45 | down | 1.26E-04 |
| KDM6B | 163.29 | 402.89 | 14 | 494 | 2.44 | down | 1.28E-04 |
| REG3A | 256.24 | 404.76 | 29 | 479 | 1.59 | down | 1.36E-04 |
| ZNF735 | 218.28 | 405.5 | 25 | 483 | 1.85 | down | 1.69E-04 |
| EIF4G1 | 181.19 | 403.28 | 16 | 492 | 2.22 | down | 1.71E-04 |
| CLTCL1 | 165.21 | 402.83 | 14 | 494 | 2.44 | down | 2.16E-04 |
| COL5A1 | 236.76 | 407.73 | 34 | 474 | 1.72 | down | 2.31E-04 |
| PKD1L2 | 228.26 | 407.2 | 31 | 477 | 1.79 | down | 2.41E-04 |

| | | | | | | | |
|---|---|---|---|---|---|---|---|
| SAMD9L | 239.45 | 406.48 | 31 | 477 | 1.69 | down | 2.78E-04 |
| CDH18 | 277.91 | 407.79 | 45 | 463 | 1.47 | down | 2.93E-04 |
| TLR4 | 279.24 | 410.49 | 55 | 453 | 1.47 | down | 2.97E-04 |
| DCAF8L1 | 199.61 | 403.51 | 18 | 490 | 2.04 | down | 3.01E-04 |
| SCN7A | 227.86 | 406.11 | 28 | 480 | 1.79 | down | 3.15E-04 |
| CNTN1 | 227.04 | 405.78 | 27 | 481 | 1.79 | down | 3.32E-04 |
| POP1 | 176.73 | 402.96 | 15 | 493 | 2.27 | down | 3.34E-04 |
| WDR78 | 191.88 | 403.36 | 17 | 491 | 2.08 | down | 3.42E-04 |
| CTNNA2 | 276.8 | 410.5 | 54 | 454 | 1.49 | down | 3.43E-04 |
| RFX4 | 197.44 | 402.75 | 16 | 492 | 2.04 | down | 3.57E-04 |
| PRAMEF18 | 179.67 | 402.87 | 15 | 493 | 2.22 | down | 3.70E-04 |
| ANO4 | 235.66 | 406.01 | 29 | 479 | 1.72 | down | 4.15E-04 |
| ABCA10 | 247.03 | 405.98 | 31 | 477 | 1.64 | down | 4.21E-04 |
| HHIPL2 | 232.26 | 404.06 | 23 | 485 | 1.75 | down | 4.29E-04 |
| VPS13C | 256.54 | 407.26 | 37 | 471 | 1.59 | down | 4.35E-04 |
| TRAPPC9 | 162.92 | 401.93 | 12 | 496 | 2.44 | down | 4.80E-04 |
| DCDC1 | 266.93 | 408.86 | 45 | 463 | 1.54 | down | 4.92E-04 |
| NPR1 | 169.08 | 402.25 | 13 | 495 | 2.38 | down | 5.02E-04 |
| OR5T3 | 222.25 | 403.42 | 20 | 488 | 1.82 | down | 5.12E-04 |
| SPEF2 | 265.31 | 409.01 | 45 | 463 | 1.54 | down | 5.16E-04 |
| DZIP3 | 242.62 | 404.57 | 26 | 482 | 1.67 | down | 5.43E-04 |
| ABCA5 | 219.5 | 404.29 | 22 | 486 | 1.85 | down | 5.52E-04 |
| OR8K3 | 198.65 | 403.13 | 17 | 491 | 2.04 | down | 5.58E-04 |
| CFAP58 | 216.18 | 402.52 | 17 | 491 | 1.85 | down | 5.76E-04 |
| ACSM2B | 229.04 | 404.58 | 24 | 484 | 1.75 | down | 5.98E-04 |
| NID1 | 243.91 | 407.56 | 35 | 473 | 1.67 | down | 6.11E-04 |
| SCN1A | 245.15 | 404.77 | 27 | 481 | 1.64 | down | 6.24E-04 |
| THSD1 | 169.58 | 401.77 | 12 | 496 | 2.38 | down | 6.56E-04 |
| CAPN6 | 214.3 | 403.74 | 20 | 488 | 1.89 | down | 6.98E-04 |

| | | | | | | | |
|---|---|---|---|---|---|---|---|
| OPLAH | 171.25 | 401.73 | 12 | 496 | 2.33 | down | 7.32E-04 |
| SLC35F1 | 174.92 | 402.1 | 13 | 495 | 2.27 | down | 7.37E-04 |
| ZSWIM2 | 236.71 | 405.59 | 28 | 480 | 1.72 | down | 7.41E-04 |
| IMPG1 | 199.35 | 403.1 | 17 | 491 | 2.04 | down | 7.46E-04 |
| COL5A2 | 277 | 409.31 | 50 | 458 | 1.47 | down | 7.48E-04 |
| CDH6 | 252.76 | 404.97 | 29 | 479 | 1.61 | down | 7.82E-04 |
| CD163 | 246.83 | 405.33 | 29 | 479 | 1.64 | down | 8.04E-04 |
| ZNF462 | 207.17 | 403.23 | 18 | 490 | 1.96 | down | 8.47E-04 |
| XIRP1 | 219.45 | 403.53 | 20 | 488 | 1.85 | down | 8.83E-04 |
| KIAA1429 | 203.69 | 402.55 | 16 | 492 | 1.96 | down | 9.00E-04 |
| KCNK9 | 215.43 | 401.41 | 14 | 494 | 1.85 | down | 9.53E-04 |
| SDK1 | 258.81 | 405.53 | 32 | 476 | 1.56 | down | 9.65E-04 |
| OR2T4 | 242.45 | 405.6 | 29 | 479 | 1.67 | down | 9.65E-04 |
| PHF3 | 227.75 | 404.64 | 24 | 484 | 1.79 | down | 9.84E-04 |
| SP140 | 206.35 | 402.86 | 17 | 491 | 1.96 | down | 1.00E-03 |
| POM121L12 | 265.95 | 407.12 | 39 | 469 | 1.54 | down | 1.02E-03 |
| DOCK5 | 196.62 | 402.78 | 16 | 492 | 2.04 | down | 1.03E-03 |
| TPR | 237.04 | 404.18 | 24 | 484 | 1.69 | down | 1.06E-03 |
| OR5M8 | 167.36 | 401.35 | 11 | 497 | 2.38 | down | 1.06E-03 |
| SELE | 180.08 | 401.96 | 13 | 495 | 2.22 | down | 1.07E-03 |
| CDH11 | 246.64 | 405.01 | 28 | 480 | 1.64 | down | 1.08E-03 |
| PREPL | 182.54 | 401.9 | 13 | 495 | 2.22 | down | 1.09E-03 |
| ADGRB1 | 203.65 | 402.95 | 17 | 491 | 1.96 | down | 1.10E-03 |
| SLC39A12 | 273.7 | 406.76 | 40 | 468 | 1.49 | down | 1.23E-03 |
| CFH | 250.59 | 406.08 | 32 | 476 | 1.61 | down | 1.24E-03 |
| KCNU1 | 257.63 | 407.49 | 38 | 470 | 1.59 | down | 1.26E-03 |
| RREB1 | 200.14 | 401.84 | 14 | 494 | 2 | down | 1.27E-03 |
| OR4K2 | 215.2 | 403.7 | 20 | 488 | 1.89 | down | 1.28E-03 |
| OR5D14 | 233.21 | 404.37 | 24 | 484 | 1.72 | down | 1.31E-03 |

| | | | | | | | |
|---|---|---|---|---|---|---|---|
| DNAH17 | 242 | 404.61 | 26 | 482 | 1.67 | down | 1.32E-03 |
| SMARCA4 | 273.91 | 407.6 | 43 | 465 | 1.49 | down | 1.38E-03 |
| FRAS1 | 255.52 | 406.06 | 33 | 475 | 1.59 | down | 1.39E-03 |
| ROS1 | 218 | 403.59 | 20 | 488 | 1.85 | down | 1.41E-03 |
| FRYL | 236.3 | 405.26 | 27 | 481 | 1.72 | down | 1.42E-03 |
| DNAH11 | 279.7 | 409.01 | 50 | 458 | 1.47 | down | 1.50E-03 |
| CRISP2 | 167.64 | 401.34 | 11 | 497 | 2.38 | down | 1.52E-03 |
| ACCSL | 183 | 401.44 | 12 | 496 | 2.17 | down | 1.53E-03 |
| ZNF658 | 182.25 | 401.46 | 12 | 496 | 2.22 | down | 1.53E-03 |
| DDX60L | 211.35 | 402.69 | 17 | 491 | 1.92 | down | 1.53E-03 |
| UBR5 | 256.93 | 404.72 | 29 | 479 | 1.59 | down | 1.54E-03 |
| BMPER | 248.9 | 402.32 | 20 | 488 | 1.61 | down | 1.56E-03 |
| GRIN2B | 284.1 | 408.8 | 51 | 457 | 1.45 | down | 1.57E-03 |
| TSHZ2 | 274.74 | 405 | 34 | 474 | 1.47 | down | 1.60E-03 |
| RLF | 199.6 | 402.27 | 15 | 493 | 2 | down | 1.64E-03 |
| HTR1E | 208.47 | 402.79 | 17 | 491 | 1.92 | down | 1.67E-03 |
| CACNA1A | 239.41 | 403.38 | 22 | 486 | 1.69 | down | 1.69E-03 |
| PIK3CG | 252.11 | 404.38 | 27 | 481 | 1.61 | down | 1.71E-03 |
| TNS3 | 210.47 | 401.94 | 15 | 493 | 1.92 | down | 1.74E-03 |
| GRM8 | 280.9 | 405.88 | 39 | 469 | 1.45 | down | 1.80E-03 |
| CNTN5 | 260.29 | 406.04 | 34 | 474 | 1.56 | down | 1.81E-03 |
| IGF2R | 268.92 | 403.15 | 26 | 482 | 1.49 | down | 1.87E-03 |
| CREBBP | 270.41 | 401.98 | 22 | 486 | 1.49 | down | 1.91E-03 |
| SMARCA1 | 229.06 | 402.43 | 18 | 490 | 1.75 | down | 1.93E-03 |
| KRT79 | 183.08 | 401.44 | 12 | 496 | 2.17 | down | 1.99E-03 |
| GON4L | 194.64 | 402 | 14 | 494 | 2.08 | down | 2.00E-03 |
| MAGEC1 | 275.64 | 408.01 | 45 | 463 | 1.47 | down | 2.01E-03 |
| OR2T1 | 248.08 | 403.63 | 24 | 484 | 1.64 | down | 2.03E-03 |
| OR6Q1 | 196 | 401.54 | 13 | 495 | 2.04 | down | 2.05E-03 |

| | | | | | | | |
|---|---|---|---|---|---|---|---|
| C10orf12 | 229.36 | 401.01 | 14 | 494 | 1.75 | down | 2.07E-03 |
| LRRC4C | 269.85 | 406.8 | 39 | 469 | 1.52 | down | 2.17E-03 |
| RPGRIP1L | 196.46 | 401.53 | 13 | 495 | 2.04 | down | 2.19E-03 |
| WDR17 | 214.29 | 402.58 | 17 | 491 | 1.89 | down | 2.19E-03 |
| APOA5 | 188.09 | 400.89 | 11 | 497 | 2.13 | down | 2.24E-03 |
| CPED1 | 266.06 | 405.62 | 34 | 474 | 1.52 | down | 2.26E-03 |
| HELZ | 215.47 | 402.54 | 17 | 491 | 1.85 | down | 2.29E-03 |
| SLITRK5 | 237.67 | 404.15 | 24 | 484 | 1.69 | down | 2.29E-03 |
| FSIP2 | 253.93 | 404.59 | 28 | 480 | 1.59 | down | 2.30E-03 |
| CAPN11 | 214.57 | 401.43 | 14 | 494 | 1.89 | down | 2.32E-03 |
| KIF5A | 235.45 | 403.56 | 22 | 486 | 1.72 | down | 2.32E-03 |
| SLC17A4 | 220 | 401.65 | 15 | 493 | 1.82 | down | 2.42E-03 |
| COL22A1 | 282.6 | 408.69 | 50 | 458 | 1.45 | down | 2.43E-03 |
| DPYS | 223.11 | 403.01 | 19 | 489 | 1.82 | down | 2.57E-03 |
| GRIK3 | 225.06 | 402.57 | 18 | 490 | 1.79 | down | 2.61E-03 |
| LZTR1 | 209.75 | 400.8 | 12 | 496 | 1.92 | down | 2.62E-03 |
| NELL2 | 243.45 | 403.2 | 22 | 486 | 1.67 | down | 2.75E-03 |
| TF | 214.69 | 401.05 | 13 | 495 | 1.85 | down | 2.77E-03 |
| TRAF3IP3 | 193.5 | 401.19 | 12 | 496 | 2.08 | down | 2.88E-03 |
| CNTN3 | 264.72 | 404.25 | 29 | 479 | 1.54 | down | 2.92E-03 |
| NLRP5 | 267.21 | 406.72 | 38 | 470 | 1.52 | down | 2.94E-03 |
| LRRC32 | 238.52 | 403.09 | 21 | 487 | 1.69 | down | 2.97E-03 |
| CASR | 236.91 | 403.5 | 22 | 486 | 1.69 | down | 3.21E-03 |
| ATP10D | 234.27 | 403.62 | 22 | 486 | 1.72 | down | 3.23E-03 |
| RUNX1T1 | 244.69 | 404.46 | 26 | 482 | 1.67 | down | 3.24E-03 |
| OR4D10 | 200.15 | 401.43 | 13 | 495 | 2 | down | 3.31E-03 |
| OR4D5 | 193.67 | 401.19 | 12 | 496 | 2.08 | down | 3.33E-03 |
| GOLGB1 | 256.32 | 402.62 | 22 | 486 | 1.56 | down | 3.38E-03 |
| USP26 | 239.81 | 403.03 | 21 | 487 | 1.67 | down | 3.40E-03 |

| | | | | | | | |
|---|---|---|---|---|---|---|---|
| PRTG | 201.46 | 401.4 | 13 | 495 | 2 | down | 3.42E-03 |
| COL27A1 | 258.57 | 402.22 | 21 | 487 | 1.56 | down | 3.43E-03 |
| DLC1 | 255.27 | 403.89 | 26 | 482 | 1.59 | down | 3.50E-03 |
| CSPG4 | 234.23 | 403.62 | 22 | 486 | 1.72 | down | 3.51E-03 |
| SNTG2 | 231.26 | 402.7 | 19 | 489 | 1.75 | down | 3.53E-03 |
| DMXL1 | 264.16 | 404.87 | 31 | 477 | 1.54 | down | 3.55E-03 |
| EPHB6 | 266.5 | 406.18 | 36 | 472 | 1.52 | down | 3.57E-03 |
| ABCC5 | 207.85 | 401.23 | 13 | 495 | 1.92 | down | 3.58E-03 |
| DYNC2H1 | 275.72 | 403.58 | 29 | 479 | 1.47 | down | 3.59E-03 |
| CALCRL | 214 | 402.21 | 16 | 492 | 1.89 | down | 3.62E-03 |
| ATOH1 | 230.07 | 401.34 | 15 | 493 | 1.75 | down | 3.63E-03 |
| CD6 | 199.5 | 401.04 | 12 | 496 | 2 | down | 3.68E-03 |
| ZNF33A | 242.11 | 402.27 | 19 | 489 | 1.67 | down | 3.78E-03 |
| FAM120A | 203.92 | 401.34 | 13 | 495 | 1.96 | down | 3.81E-03 |
| SLC17A3 | 235.29 | 400.85 | 14 | 494 | 1.69 | down | 3.85E-03 |
| CFHR5 | 229.47 | 401.36 | 15 | 493 | 1.75 | down | 3.87E-03 |
| SHOX2 | 181.73 | 401.03 | 11 | 497 | 2.22 | down | 3.87E-03 |
| ZNF292 | 255.29 | 402.36 | 21 | 487 | 1.59 | down | 3.91E-03 |
| TRPM7 | 194 | 400.76 | 11 | 497 | 2.08 | down | 4.01E-03 |
| CALCR | 240.82 | 403.32 | 22 | 486 | 1.67 | down | 4.03E-03 |
| OR8H3 | 248.05 | 402.99 | 22 | 486 | 1.61 | down | 4.05E-03 |
| MMRN1 | 281.36 | 406.64 | 42 | 466 | 1.45 | down | 4.23E-03 |
| BRINP1 | 257.35 | 401.98 | 20 | 488 | 1.56 | down | 4.25E-03 |
| CDH23 | 272.56 | 405.72 | 36 | 472 | 1.49 | down | 4.25E-03 |
| CTNNA3 | 251.04 | 403.49 | 24 | 484 | 1.61 | down | 4.26E-03 |
| HIPK1 | 196 | 400.72 | 11 | 497 | 2.04 | down | 4.28E-03 |
| SPATA16 | 192.82 | 400.79 | 11 | 497 | 2.08 | down | 4.28E-03 |
| SPHKAP | 283.71 | 408.3 | 49 | 459 | 1.45 | down | 4.40E-03 |
| NLGN1 | 257 | 404.41 | 28 | 480 | 1.56 | down | 4.41E-03 |

| | | | | | | | |
|---|---|---|---|---|---|---|---|
| LATS1 | 222.13 | 401.58 | 15 | 493 | 1.82 | down | 4.45E-03 |
| COL20A1 | 243.09 | 403.22 | 22 | 486 | 1.67 | down | 4.60E-03 |
| F13B | 242.86 | 402.9 | 21 | 487 | 1.67 | down | 4.60E-03 |
| CPXM2 | 241.33 | 402.97 | 21 | 487 | 1.67 | down | 4.69E-03 |
| PRSS55 | 202.92 | 400.96 | 12 | 496 | 1.96 | down | 4.82E-03 |
| PRKDC | 276.95 | 406.76 | 41 | 467 | 1.47 | down | 4.99E-03 |
| CORIN | 230.18 | 402.03 | 17 | 491 | 1.75 | down | 5.01E-03 |
| DPP10 | 261.16 | 403.28 | 25 | 483 | 1.54 | down | 5.11E-03 |
| NOTCH2 | 223.35 | 402.27 | 17 | 491 | 1.79 | down | 5.19E-03 |
| DSC1 | 226.2 | 401.46 | 15 | 493 | 1.79 | down | 5.20E-03 |
| C2orf16 | 232.71 | 401.95 | 17 | 491 | 1.72 | down | 5.21E-03 |
| ARHGAP33 | 197.45 | 400.68 | 11 | 497 | 2.04 | down | 5.22E-03 |
| USP24 | 218.4 | 401.7 | 15 | 493 | 1.85 | down | 5.24E-03 |
| DTNA | 264.26 | 402.54 | 23 | 485 | 1.52 | down | 5.26E-03 |
| ADAM23 | 274.71 | 402.31 | 24 | 484 | 1.47 | down | 5.27E-03 |
| FHL5 | 220.81 | 401.99 | 16 | 492 | 1.82 | down | 5.32E-03 |
| PIK3C2G | 224.65 | 402.23 | 17 | 491 | 1.79 | down | 5.41E-03 |
| INSR | 209.69 | 401.18 | 13 | 495 | 1.92 | down | 5.51E-03 |
| OR6K2 | 243.42 | 403.86 | 24 | 484 | 1.67 | down | 5.52E-03 |
| OR8K1 | 228.18 | 402.1 | 17 | 491 | 1.75 | down | 5.54E-03 |
| SLC7A14 | 219.14 | 401.3 | 14 | 494 | 1.82 | down | 5.55E-03 |
| OR4D6 | 266.07 | 399.97 | 14 | 494 | 1.49 | down | 5.61E-03 |
| COBL | 254.91 | 402.68 | 22 | 486 | 1.59 | down | 5.72E-03 |
| ENAM | 227.62 | 400.71 | 13 | 495 | 1.75 | down | 5.81E-03 |
| SLC6A15 | 244.57 | 402.83 | 21 | 487 | 1.64 | down | 5.84E-03 |
| CACNA2D1 | 273.86 | 405.9 | 37 | 471 | 1.49 | down | 5.89E-03 |
| AMY2B | 269.19 | 400.42 | 16 | 492 | 1.49 | down | 5.91E-03 |
| USP29 | 258.78 | 405.53 | 32 | 476 | 1.56 | down | 6.01E-03 |
| PRAMEF19 | 201.75 | 400.99 | 12 | 496 | 2 | down | 6.17E-03 |

| | | | | | | | |
|---|---|---|---|---|---|---|---|
| HEPHL1 | 242.45 | 402.59 | 20 | 488 | 1.67 | down | 6.23E-03 |
| ATP2B3 | 263.08 | 403.47 | 26 | 482 | 1.54 | down | 6.27E-03 |
| SMYD1 | 226.19 | 401.82 | 16 | 492 | 1.79 | down | 6.37E-03 |
| CADM3 | 203.27 | 400.56 | 11 | 497 | 1.96 | down | 6.48E-03 |
| FBN1 | 260.43 | 402.73 | 23 | 485 | 1.54 | down | 6.50E-03 |
| UBR1 | 231.08 | 400.28 | 12 | 496 | 1.72 | down | 6.59E-03 |
| TSC2 | 201.18 | 400.6 | 11 | 497 | 2 | down | 6.64E-03 |
| CD109 | 248.4 | 402.34 | 20 | 488 | 1.61 | down | 6.81E-03 |
| NME8 | 241.75 | 401.31 | 16 | 492 | 1.67 | down | 6.98E-03 |
| CNST | 202.67 | 400.97 | 12 | 496 | 1.96 | down | 7.00E-03 |
| PYHIN1 | 271.42 | 403.02 | 26 | 482 | 1.49 | down | 7.08E-03 |
| PIWIL1 | 204.85 | 401.31 | 13 | 495 | 1.96 | down | 7.16E-03 |
| SGIP1 | 265.7 | 404.48 | 30 | 478 | 1.52 | down | 7.18E-03 |
| PCDHA8 | 222.47 | 401.57 | 15 | 493 | 1.82 | down | 7.19E-03 |
| KCNB1 | 259.68 | 402.47 | 22 | 486 | 1.54 | down | 7.21E-03 |
| OR4A5 | 267.75 | 403.78 | 28 | 480 | 1.52 | down | 7.29E-03 |
| CAD | 259.29 | 402.19 | 21 | 487 | 1.56 | down | 7.38E-03 |
| CYP11B1 | 254.52 | 403.01 | 23 | 485 | 1.59 | down | 7.41E-03 |
| ASIC2 | 220.45 | 400.18 | 11 | 497 | 1.82 | down | 7.43E-03 |
| JMJD1C | 259.24 | 402.19 | 21 | 487 | 1.56 | down | 7.57E-03 |
| ST18 | 267.04 | 403.26 | 26 | 482 | 1.52 | down | 7.71E-03 |
| PREX2 | 259.08 | 403.39 | 25 | 483 | 1.56 | down | 7.72E-03 |
| C2CD3 | 252.46 | 403.42 | 24 | 484 | 1.59 | down | 7.80E-03 |
| TDRD5 | 248.17 | 401.72 | 18 | 490 | 1.61 | down | 7.91E-03 |
| POTEC | 233.67 | 402.26 | 18 | 490 | 1.72 | down | 8.10E-03 |
| ZNF676 | 250.87 | 403.18 | 23 | 485 | 1.61 | down | 8.11E-03 |
| ANKFN1 | 228.07 | 401.4 | 15 | 493 | 1.75 | down | 8.21E-03 |
| LILRA1 | 264.14 | 400.03 | 14 | 494 | 1.52 | down | 8.22E-03 |
| ZNF862 | 207.09 | 400.47 | 11 | 497 | 1.92 | down | 8.23E-03 |

| | | | | | | | |
|---|---|---|---|---|---|---|---|
| RNF20 | 230.93 | 400.97 | 14 | 494 | 1.72 | down | 8.56E-03 |
| PCK1 | 263.18 | 402.31 | 22 | 486 | 1.54 | down | 8.58E-03 |
| KDM4C | 209.15 | 401.2 | 13 | 495 | 1.92 | down | 8.61E-03 |
| SLC4A5 | 203.27 | 400.56 | 11 | 497 | 1.96 | down | 8.75E-03 |
| SF3B2 | 198.91 | 400.65 | 11 | 497 | 2 | down | 8.83E-03 |
| PRKD1 | 261.91 | 402.66 | 23 | 485 | 1.54 | down | 8.95E-03 |
| MYF5 | 207.64 | 400.46 | 11 | 497 | 1.92 | down | 9.10E-03 |
| OR10G7 | 224.71 | 401.15 | 14 | 494 | 1.79 | down | 9.11E-03 |
| TBX22 | 275.89 | 403.04 | 27 | 481 | 1.47 | down | 9.18E-03 |
| RICTOR | 274.16 | 401.03 | 19 | 489 | 1.47 | down | 9.22E-03 |
| TRPV6 | 251.14 | 402.85 | 22 | 486 | 1.61 | down | 9.32E-03 |
| CHRDL1 | 235.65 | 401.85 | 17 | 491 | 1.69 | down | 9.46E-03 |
| ASXL2 | 246.24 | 401.48 | 17 | 491 | 1.64 | down | 9.49E-03 |
| NLRP10 | 270.34 | 403.91 | 29 | 479 | 1.49 | down | 9.58E-03 |
| GABRR1 | 240.64 | 399.73 | 11 | 497 | 1.67 | down | 9.61E-03 |
| PLCXD3 | 245.64 | 400.55 | 14 | 494 | 1.64 | down | 9.72E-03 |
| SERPINB3 | 249.43 | 402.62 | 21 | 487 | 1.61 | down | 9.80E-03 |

**Supplementary Table 7. Mutated genes in LUSC that significantly correlate with altered ACOX2 expression**

| Mutation of | Mean expression (mutant) | Mean expression (wild) | Number of mutant | Number of wild | FC (mutant/wild) | Direction | p-value |
|---|---|---|---|---|---|---|---|
| RASA1 | 119.3 | 72.45 | 30 | 458 | 1.65 | up | 1.53E-05 |
| PTCHD2 | 45.53 | 76.27 | 15 | 473 | 1.67 | down | 7.22E-05 |
| AMER3 | 109.07 | 73.12 | 30 | 458 | 1.49 | up | 9.49E-04 |
| GLRA2 | 136.5 | 73.79 | 12 | 476 | 1.85 | up | 9.56E-04 |
| PCSK5 | 146.12 | 72.93 | 16 | 472 | 2 | up | 9.73E-04 |
| BRCA2 | 44.07 | 77.38 | 30 | 458 | 1.75 | down | 1.17E-03 |
| NBEA | 41.18 | 77.41 | 28 | 460 | 1.89 | down | 1.42E-03 |
| CHD5 | 127.91 | 72.85 | 22 | 466 | 1.76 | up | 1.50E-03 |
| CEP162 | 46.68 | 76.68 | 22 | 466 | 1.64 | down | 1.56E-03 |
| UHRF1BP1L | 117 | 73.83 | 17 | 471 | 1.58 | up | 1.79E-03 |
| HTR1A | 119.93 | 74.01 | 14 | 474 | 1.62 | up | 2.00E-03 |
| STRC | 179.4 | 73.15 | 10 | 478 | 2.45 | up | 2.19E-03 |
| SUPT5H | 36.14 | 76.49 | 14 | 474 | 2.13 | down | 2.49E-03 |
| ADAMTSL1 | 123.92 | 74 | 13 | 475 | 1.67 | up | 2.53E-03 |
| HECW2 | 50.11 | 76.87 | 28 | 460 | 1.54 | down | 2.75E-03 |
| PTPRT | 109.03 | 72.96 | 32 | 456 | 1.49 | up | 3.27E-03 |
| BACH2 | 141.1 | 73.95 | 10 | 478 | 1.91 | up | 3.40E-03 |
| SPG7 | 29.1 | 76.3 | 10 | 478 | 2.63 | down | 3.82E-03 |
| CDKN2A | 54.63 | 79.09 | 75 | 413 | 1.45 | down | 3.93E-03 |
| RAG2 | 37.67 | 76.28 | 12 | 476 | 2.04 | down | 3.95E-03 |
| NFE2L2 | 51.52 | 79.52 | 73 | 415 | 1.54 | down | 4.38E-03 |
| TSPEAR | 111.1 | 74.58 | 10 | 478 | 1.49 | up | 5.17E-03 |
| JAKMIP1 | 141.41 | 72.94 | 17 | 471 | 1.94 | up | 5.23E-03 |
| OR5A2 | 34.5 | 76.36 | 12 | 476 | 2.22 | down | 6.43E-03 |

| | | | | | | | |
|---|---|---|---|---|---|---|---|
| DAPK1 | 33 | 76.31 | 11 | 477 | 2.33 | down | 6.98E-03 |
| CPAMD8 | 41.74 | 76.99 | 23 | 465 | 1.85 | down | 7.07E-03 |
| IGSF10 | 37.06 | 76.71 | 17 | 471 | 2.08 | down | 7.41E-03 |
| FAM65B | 33.5 | 76.21 | 10 | 478 | 2.27 | down | 7.47E-03 |
| ST6GAL2 | 108.73 | 74.56 | 11 | 477 | 1.46 | up | 7.59E-03 |
| TDRD6 | 40.63 | 76.74 | 19 | 469 | 1.89 | down | 8.36E-03 |
| PCDH11Y | 48.35 | 76.85 | 26 | 462 | 1.59 | down | 8.50E-03 |
| LRRC6 | 35.45 | 76.25 | 11 | 477 | 2.17 | down | 8.71E-03 |
| SLC22A8 | 115.9 | 74.48 | 10 | 478 | 1.56 | up | 8.87E-03 |
| MAGEA4 | 109.92 | 74.38 | 13 | 475 | 1.48 | up | 9.58E-03 |
| WT1 | 33.09 | 76.3 | 11 | 477 | 2.33 | down | 9.88E-03 |

**Supplementary Table 8: Methylation Analysis using Mexpress**

| Probe ID | TCGA-LUAD Mexpress | TCGA-LUAD Wanderer | TCGA-LUSC Mexpress | TCGA-LUSC Wanderer |
|---|---|---|---|---|
| Promoter Specific | | | | |
| cg22012981 | absent | nd | absent | nd |
| cg16209444 | r = -0.676*** | ns | r = -0.498*** | **<0.05** |
| cg16587010 | r = -0.578*** | ns | r = -0.365*** | **<0.05** |
| cg13705284 | r = -0.556*** | **<0.05** | r = -0.241*** | **<0.05** |
| cg02259384 | r = -0.577*** | ns | r = -0.181*** | **<0.05** |
| | | | | |
| Other | | | | |
| cg12075202 | r = 0.187*** | **<0.05** | r = 0.081 | **<0.05** |
| cg23652987 | r = -0.039 | **<0.05** | r = 0.024 | **<0.05** |
| cg16869862 | r = 0.230*** | ns | r = 0.123* | **<0.05** |
| | | | | |

(*cg16587010, cg13705284, cg02259384* are the probes in UALCAN methylation analysis)

Pearson correlation coefficients are provided for Mexpress [6] analysis; Adjusted p-value for Wanderer [7].

nd - not determined; ns - not significant

**Table 9: TIMER2 analysis of Immune Cell infiltrations associated with ACOX2 expression in LUAD**

| Infiltrates | rho | p | adj.p |
|---|---|---|---|
| **B Cells** | | | |
| B cell memory_CIBERSORT | 0.078344 | 0.08225 | 0.23169 |
| B cell memory_CIBERSORT-ABS | 0.070577 | 0.117574 | 0.292408 |
| B cell memory_XCELL | -0.12347 | 0.006051 | 0.03491 |
| B cell naive_CIBERSORT | -0.00024 | 0.995813 | 0.997475 |
| B cell naive_CIBERSORT-ABS | 0.011313 | 0.802146 | 0.89769 |
| B cell naive_XCELL | -0.06378 | 0.15737 | 0.345867 |
| B cell plasma_CIBERSORT | -0.05299 | 0.240223 | 0.454703 |
| B cell plasma_CIBERSORT-ABS | -0.02053 | 0.649257 | 0.807396 |
| B cell plasma_XCELL | -0.12751 | 0.004574 | 0.03016 |
| B cell_EPIC | -0.05039 | 0.264075 | 0.47297 |
| B cell_MCPCOUNTER | -0.00504 | 0.911127 | 0.939306 |
| B cell_QUANTISEQ | -0.10633 | 0.018195 | 0.07688 |
| B cell_TIMER | 0.058528 | 0.194514 | 0.395705 |
| B cell_XCELL | -0.12115 | 0.007078 | 0.039152 |
| Class-switched memory B cell_XCELL | 0.059555 | 0.186783 | 0.387786 |
| **CD8+ T Cells** | | | |
| T cell CD8+ central memory_XCELL | -0.130134837 | 0.003798293 | 0.019991018 |
| T cell CD8+ effector memory_XCELL | -0.13023406 | 0.00377145 | 0.019991018 |
| T cell CD8+ naive_XCELL | -0.046826111 | 0.299438432 | 0.541889398 |
| T cell CD8+_CIBERSORT | -0.202849597 | 5.63E-06 | 8.66E-05 |
| T cell CD8+_CIBERSORT-ABS | -0.1187435 | 0.008310583 | 0.036935925 |
| T cell CD8+_EPIC | -0.001016259 | 0.982043256 | 0.996522938 |
| T cell CD8+_MCPCOUNTER | -0.188841194 | 2.44E-05 | 0.000314751 |
| T cell CD8+_QUANTISEQ | -0.128478919 | 0.004272563 | 0.022195135 |
| T cell CD8+_TIMER | -0.170408777 | 0.000143575 | 0.001472561 |
| T cell CD8+_XCELL | -0.074645876 | 0.097821003 | 0.26260672 |
| **CD4+ T Cells** | | | |
| T cell CD4+ (non-regulatory)_QUANTISEQ | 0.062359 | 0.166844 | 0.341816 |
| T cell CD4+ (non-regulatory)_XCELL | 0.070802 | 0.116406 | 0.260056 |
| T cell CD4+ central memory_XCELL | 0.158832 | 0.0004 | 0.002332 |
| T cell CD4+ effector memory_XCELL | 0.158223 | 0.000421 | 0.002434 |
| T cell CD4+ memory activated_CIBERSORT | -0.23322 | 1.63E-07 | 2.33E-06 |
| T cell CD4+ memory activated_CIBERSORT-ABS | -0.23525 | 1.26E-07 | 1.89E-06 |
| T cell CD4+ memory resting_CIBERSORT | 0.222324 | 6.15E-07 | 7.75E-06 |
| T cell CD4+ memory resting_CIBERSORT-ABS | 0.19934 | 8.21E-06 | 7.60E-05 |
| T cell CD4+ memory_XCELL | -0.1377 | 0.002183 | 0.010577 |
| T cell CD4+ naïve_CIBERSORT | 0.011078 | 0.806187 | 0.897563 |
| T cell CD4+ naïve_CIBERSORT-ABS | 0.011078 | 0.806187 | 0.897563 |

| | | | |
|---|---|---|---|
| T cell CD4+ naïve_XCELL | 0.062563 | 0.165454 | 0.34064 |
| T cell CD4+ Th1_XCELL | -0.19824 | 9.23E-06 | 8.35E-05 |
| T cell CD4+ Th2_XCELL | -0.37975 | 2.33E-18 | 4.90E-16 |
| T cell CD4+_EPIC | 0.05023 | 0.265642 | 0.457252 |
| T cell CD4+_TIMER | 0.064152 | 0.15495 | 0.32324 |
| **Macrophages** | | | |
| Macrophage M0_CIBERSORT | -0.17497 | 9.41E-05 | 0.000513 |
| Macrophage M0_CIBERSORT-ABS | -0.17076 | 0.000139 | 0.000725 |
| Macrophage M1_CIBERSORT | -0.24653 | 2.93E-08 | 3.51E-07 |
| Macrophage M1_CIBERSORT-ABS | -0.18083 | 5.39E-05 | 0.00032 |
| Macrophage M1_QUANTISEQ | 0.149214 | 0.00089 | 0.003681 |
| Macrophage M1_XCELL | -0.21084 | 2.33E-06 | 1.84E-05 |
| Macrophage M2_CIBERSORT | 0.126024 | 0.005075 | 0.016729 |
| Macrophage M2_CIBERSORT-ABS | 0.12436 | 0.005693 | 0.018266 |
| Macrophage M2_QUANTISEQ | 0.216947 | 1.16E-06 | 9.78E-06 |
| Macrophage M2_TIDE | 0.246922 | 2.78E-08 | 3.40E-07 |
| Macrophage M2_XCELL | 0.026921 | 0.550958 | 0.701858 |
| Macrophage_EPIC | -0.09544 | 0.034131 | 0.083586 |
| Macrophage_TIMER | -0.12269 | 0.00638 | 0.020146 |
| Macrophage_XCELL | -0.11666 | 0.009529 | 0.028444 |
| Macrophage/Monocyte_MCPCOUNTER | -0.11119 | 0.013499 | 0.038569 |
| **Neutrophils** | | | |
| Neutrophil_CIBERSORT | 0.072769 | 0.106577 | 0.257292 |
| Neutrophil_CIBERSORT-ABS | 0.077337 | 0.086278 | 0.217058 |
| Neutrophil_MCPCOUNTER | 0.341431 | 6.33E-15 | 3.78E-13 |
| Neutrophil_QUANTISEQ | 0.200149 | 7.53E-06 | 8.18E-05 |
| Neutrophil_TIMER | -0.09861 | 0.028572 | 0.102121 |
| Neutrophil_XCELL | -0.03459 | 0.443446 | 0.650205 |
| **Dendritic Cells** | | | |
| Myeloid dendritic cell activated_CIBERSORT | 0.204954 | 4.48E-06 | 5.46E-05 |
| Myeloid dendritic cell activated_CIBERSORT-ABS | 0.221037 | 7.17E-07 | 1.19E-05 |
| Myeloid dendritic cell activated_XCELL | 0.02504 | 0.579125 | 0.760698 |
| Myeloid dendritic cell resting_CIBERSORT | 0.200318 | 7.39E-06 | 7.74E-05 |
| Myeloid dendritic cell resting_CIBERSORT-ABS | 0.201678 | 6.39E-06 | 7.06E-05 |
| Myeloid dendritic cell_MCPCOUNTER | 0.289953 | 5.27E-11 | 1.61E-09 |
| Myeloid dendritic cell_QUANTISEQ | -0.12311 | 0.006202 | 0.028373 |
| Myeloid dendritic cell_TIMER | 0.065421 | 0.146936 | 0.321322 |
| Myeloid dendritic cell_XCELL | 0.181924 | 4.84E-05 | 0.000387 |
| Plasmacytoid dendritic cell_XCELL | -0.28638 | 9.25E-11 | 2.63E-09 |

Results are presented as purity-corrected partial Spearman’s rho value and statistical significance. Neg – negative correlation ($p<0.05$, $\rho<0$); Pos – Positive correlation ($p<0.05$, $\rho>0$); ns – not significant ($p>0.05$)

**Table 10. TIMER2 analysis of Immune Cell infiltrations associated with ACOX2 expression in LUSC**

| Infiltrates | rho | p | adj.p |
|---|---|---|---|
| **B Cells** | | | |
| B cell memory_CIBERSORT | 0.101768 | 0.026243 | 0.100935 |
| B cell memory_CIBERSORT-ABS | 0.131772 | 0.003939 | 0.026979 |
| B cell memory_XCELL | -0.01577 | 0.731264 | 0.859496 |
| B cell naive_CIBERSORT | 0.024136 | 0.598998 | 0.764679 |
| B cell naive_CIBERSORT-ABS | 0.096389 | 0.03533 | 0.123244 |
| B cell naive_XCELL | -0.07261 | 0.113262 | 0.283156 |
| B cell plasma_CIBERSORT | -0.12992 | 0.004483 | 0.029886 |
| B cell plasma_CIBERSORT-ABS | 0.056214 | 0.220393 | 0.432143 |
| B cell plasma_XCELL | -0.08534 | 0.062539 | 0.189512 |
| B cell_EPIC | 0.112166 | 0.014244 | 0.064259 |
| B cell_MCPCOUNTER | 0.159908 | 0.000455 | 0.004629 |
| B cell_QUANTISEQ | 0.081562 | 0.075135 | 0.214672 |
| B cell_TIMER | 0.022138 | 0.629604 | 0.790298 |
| B cell_XCELL | 0.014828 | 0.746691 | 0.866566 |
| Class-switched memory B cell_XCELL | 0.03774 | 0.410858 | 0.621997 |
| **CD8+ T Cells** | | | |
| T cell CD8+ central memory_XCELL | 0.082097421 | 0.073236307 | 0.215799806 |
| T cell CD8+ effector memory_XCELL | 0.016312418 | 0.722321794 | 0.88628441 |
| T cell CD8+ naive_XCELL | -0.153298807 | 0.000781718 | 0.005899759 |
| T cell CD8+_CIBERSORT | -0.088353551 | 0.053807821 | 0.173573616 |
| T cell CD8+_CIBERSORT-ABS | 0.151358864 | 0.000912573 | 0.006636897 |
| T cell CD8+_EPIC | -0.057679876 | 0.208576474 | 0.43680937 |
| T cell CD8+_MCPCOUNTER | 0.153651449 | 0.000759883 | 0.005845257 |
| T cell CD8+_QUANTISEQ | 0.223228608 | 8.44E-07 | 1.89E-05 |
| T cell CD8+_TIMER | 0.274665079 | 1.06E-09 | 5.28E-08 |
| T cell CD8+_XCELL | 0.021994757 | 0.631817441 | 0.83408243 |
| **CD4+ T Cells** | | | |
| T cell CD4+ (non-regulatory)_QUANTISEQ | -0.15559 | 0.000649 | 0.003558 |
| T cell CD4+ (non-regulatory)_XCELL | -0.04611 | 0.314938 | 0.50358 |
| T cell CD4+ central memory_XCELL | -0.08057 | 0.078762 | 0.196126 |
| T cell CD4+ effector memory_XCELL | 0.230939 | 3.40E-07 | 4.47E-06 |
| T cell CD4+ memory activated_CIBERSORT | -0.08995 | 0.049595 | 0.138867 |
| T cell CD4+ memory activated_CIBERSORT-ABS | -0.06877 | 0.133648 | 0.288351 |
| T cell CD4+ memory resting_CIBERSORT | 0.24102 | 9.88E-08 | 1.56E-06 |
| T cell CD4+ memory resting_CIBERSORT-ABS | 0.356206 | 1.02E-15 | 1.61E-13 |
| T cell CD4+ memory_XCELL | -0.11274 | 0.013753 | 0.05067 |
| T cell CD4+ naïve_CIBERSORT | -0.10176 | 0.02626 | 0.088944 |

| | | | |
|---|---|---|---|
| T cell CD4+ naïve_CIBERSORT-ABS | -0.10157 | 0.026542 | 0.08932 |
| T cell CD4+ naïve_XCELL | 0.155982 | 0.000629 | 0.003477 |
| T cell CD4+ Th1_XCELL | -0.13976 | 0.002217 | 0.010663 |
| T cell CD4+ Th2_XCELL | -0.24937 | 3.40E-08 | 6.12E-07 |
| T cell CD4+_EPIC | -0.02626 | 0.567195 | 0.741355 |
| T cell CD4+_TIMER | 0.164644 | 0.000305 | 0.001847 |
| **Macrophages** | | | |
| Macrophage M0_CIBERSORT | 0.043703 | 0.340876 | 0.501288 |
| Macrophage M0_CIBERSORT-ABS | 0.190502 | 2.81E-05 | 0.000178 |
| Macrophage M1_CIBERSORT | -0.05014 | 0.274423 | 0.433498 |
| Macrophage M1_CIBERSORT-ABS | 0.14258 | 0.001798 | 0.006958 |
| Macrophage M1_QUANTISEQ | 0.426531 | 1.65E-22 | 1.98E-20 |
| Macrophage M1_XCELL | 0.262462 | 5.90E-09 | 8.23E-08 |
| Macrophage M2_CIBERSORT | 0.191817 | 2.47E-05 | 0.000158 |
| Macrophage M2_CIBERSORT-ABS | 0.428859 | 9.20E-23 | 1.38E-20 |
| Macrophage M2_QUANTISEQ | 0.325275 | 3.23E-13 | 9.69E-12 |
| Macrophage M2_TIDE | -0.23444 | 2.23E-07 | 2.16E-06 |
| Macrophage M2_XCELL | 0.325462 | 3.12E-13 | 9.69E-12 |
| Macrophage_EPIC | 0.378207 | 1.14E-17 | 6.86E-16 |
| Macrophage_TIMER | 0.328222 | 1.92E-13 | 6.39E-12 |
| Macrophage_XCELL | 0.290356 | 1.02E-10 | 2.10E-09 |
| Macrophage/Monocyte_MCPCOUNTER | 0.269064 | 2.35E-09 | 3.81E-08 |
| **Neutrophils** | | | |
| Neutrophil_CIBERSORT | 0.147395 | 0.001245 | 0.007006 |
| Neutrophil_CIBERSORT-ABS | 0.205924 | 5.78E-06 | 6.91E-05 |
| Neutrophil_MCPCOUNTER | 0.093091 | 0.042131 | 0.127459 |
| Neutrophil_QUANTISEQ | 0.180885 | 7.10E-05 | 0.000678 |
| Neutrophil_TIMER | 0.227269 | 5.26E-07 | 8.99E-06 |
| Neutrophil_XCELL | 0.125445 | 0.00608 | 0.028206 |
| **Dendritic Cells** | | | |
| Myeloid dendritic cell activated_CIBERSORT | -0.05154 | 0.261245 | 0.479149 |
| Myeloid dendritic cell activated_CIBERSORT-ABS | 0.018251 | 0.690922 | 0.840939 |
| Myeloid dendritic cell activated_XCELL | 0.163996 | 0.000322 | 0.002069 |
| Myeloid dendritic cell resting_CIBERSORT | -0.02861 | 0.533028 | 0.732911 |
| Myeloid dendritic cell resting_CIBERSORT-ABS | 0.032319 | 0.481317 | 0.703343 |
| Myeloid dendritic cell_MCPCOUNTER | 0.311338 | 3.51E-12 | 1.75E-10 |
| Myeloid dendritic cell_QUANTISEQ | -0.06686 | 0.144822 | 0.321322 |
| Myeloid dendritic cell_TIMER | 0.372871 | 3.51E-17 | 2.80E-15 |
| Myeloid dendritic cell_XCELL | 0.3067 | 7.57E-12 | 2.74E-10 |
| Plasmacytoid dendritic cell_XCELL | 0.046856 | 0.307145 | 0.544245 |

**Supplementary Table 11: ESTIMATEscores**

| sample_id | cohort | StromalScore | ImmuneScore | ESTIMATEScore | TumorPurity |
|---|---|---|---|---|---|
| TCGA.MP.A4T4.01A | LUAD | 858.8188034 | 2308.270925 | 3167.089728 | 0.48023016 |
| TCGA.05.4250.01A | LUAD | 477.7867942 | 1595.301304 | 2073.088098 | 0.61430418 |
| TCGA.64.5774.01A | LUAD | -568.3290526 | -124.3448233 | -692.6738758 | 0.875990515 |
| TCGA.97.7937.01A | LUAD | -179.7864599 | 181.1190544 | 1.332594477 | 0.822398108 |
| TCGA.97.A4M6.01A | LUAD | 456.3642942 | 2085.539407 | 2541.903702 | 0.558591872 |
| TCGA.44.A4SU.01A | LUAD | -97.98412203 | 914.0355667 | 816.0514447 | 0.748649213 |
| TCGA.78.7155.01A | LUAD | -1163.739514 | -375.7926291 | -1539.532143 | 0.929034447 |
| TCGA.78.7163.01A | LUAD | -1716.708642 | -56.55337186 | -1773.262014 | 0.941179266 |
| TCGA.55.7574.01A | LUAD | 1131.074553 | 2362.098112 | 3493.172665 | 0.437711577 |
| TCGA.78.7535.01A | LUAD | -21.00145372 | 1507.917993 | 1486.916539 | 0.679841897 |
| TCGA.55.8619.01A | LUAD | 720.4207665 | 2506.858232 | 3227.278998 | 0.472461888 |
| TCGA.95.7567.01A | LUAD | 206.5874862 | 1105.242755 | 1311.830241 | 0.698463095 |
| TCGA.44.7670.01A | LUAD | -1007.675504 | 9.545489954 | -998.1300144 | 0.896729231 |
| TCGA.44.6777.11A | LUAD | 1396.996974 | 2537.154195 | 3934.151169 | 0.3786353 |
| TCGA.55.6979.11A | LUAD | 853.193386 | 2783.401153 | 3636.594539 | 0.418687201 |
| TCGA.44.3396.11A | LUAD | 1440.441324 | 2616.362979 | 4056.804303 | 0.361911253 |
| TCGA.NJ.A7XG.01A | LUAD | -1579.740982 | -167.5570405 | -1747.298022 | 0.939884595 |
| TCGA.44.8117.01A | LUAD | 277.6375229 | 627.3598556 | 904.9973785 | 0.739929809 |
| TCGA.62.8402.01A | LUAD | -633.2400194 | 1675.497572 | 1042.257553 | 0.726227169 |
| TCGA.49.4507.01A | LUAD | -163.6432137 | 1824.767412 | 1661.124198 | 0.660868473 |
| TCGA.J2.A4AD.01A | LUAD | -340.6675695 | -121.7425703 | -462.4101398 | 0.859190563 |
| TCGA.55.A490.01A | LUAD | 325.7093945 | 1014.53812 | 1340.247515 | 0.695471834 |
| TCGA.67.3770.01A | LUAD | 120.4171795 | 1589.583482 | 1710.000661 | 0.655467037 |
| TCGA.55.A48Y.01A | LUAD | 650.5078371 | 854.6308222 | 1505.138659 | 0.677877882 |
| TCGA.44.3918.01A | LUAD | 1288.779577 | 2403.475602 | 3692.255179 | 0.411253577 |
| TCGA.05.4249.01A | LUAD | 217.599962 | 1366.30547 | 1583.905432 | 0.669332639 |
| TCGA.67.4679.01B | LUAD | 581.7052743 | 1525.250525 | 2106.955799 | 0.610373837 |
| TCGA.49.6742.11A | LUAD | 586.6990102 | 1662.635395 | 2249.334405 | 0.593686947 |
| TCGA.55.A494.01A | LUAD | -1238.304987 | -852.9442138 | -2091.249201 | 0.955920898 |
| TCGA.64.5778.01A | LUAD | -265.7749575 | 2179.191686 | 1913.416729 | 0.632627897 |
| TCGA.50.5931.11A | LUAD | 782.3995031 | 1923.629487 | 2706.02899 | 0.538449033 |
| TCGA.49.AARQ.01A | LUAD | -934.3904987 | 1012.285032 | 77.89453342 | 0.81595264 |
| TCGA.95.8494.01A | LUAD | -361.4374867 | 1168.30375 | 806.8662635 | 0.749542396 |
| TCGA.55.7726.01A | LUAD | 1116.594166 | 934.0193915 | 2050.613557 | 0.616903974 |
| TCGA.44.6147.11A | LUAD | 948.708204 | 2275.613149 | 3224.321353 | 0.47284448 |
| TCGA.78.8660.01A | LUAD | 45.86763911 | 2037.313118 | 2083.180757 | 0.613134512 |
| TCGA.49.4490.01A | LUAD | 233.8806534 | 766.2918692 | 1000.172523 | 0.730460102 |
| TCGA.J2.8194.01A | LUAD | 309.2883041 | 1017.254774 | 1326.543078 | 0.696915905 |
| TCGA.44.2659.01A | LUAD | 769.316541 | 1681.613465 | 2450.930006 | 0.569618018 |
| TCGA.78.7633.01A | LUAD | -465.0010804 | -130.5886678 | -595.5897482 | 0.869028223 |
| TCGA.55.8299.01A | LUAD | 1388.312161 | 2428.995873 | 3817.308034 | 0.394453072 |
| TCGA.67.6217.01A | LUAD | 204.7049312 | 1626.697337 | 1831.402268 | 0.641905325 |
| TCGA.67.3774.01A | LUAD | 211.4613218 | 1553.686296 | 1765.147618 | 0.649332142 |
| TCGA.MP.A4T6.01A | LUAD | -1012.408768 | 1207.04358 | 194.6348116 | 0.805926615 |
| TCGA.50.5942.01A | LUAD | 131.3881455 | 849.2555856 | 980.643731 | 0.732414858 |
| TCGA.55.A48Z.01A | LUAD | 658.3878321 | 1180.245311 | 1838.633143 | 0.641091093 |
| TCGA.99.AA5R.01A | LUAD | 1034.233144 | 2691.981959 | 3726.215103 | 0.406704624 |
| TCGA.86.8280.01A | LUAD | 799.0270883 | 2021.12613 | 2820.153218 | 0.524257838 |
| TCGA.49.6761.11A | LUAD | 720.115753 | 2230.176598 | 2950.292351 | 0.507895904 |
| TCGA.83.5908.01A | LUAD | 365.6910669 | 2222.962531 | 2588.653598 | 0.552886845 |
| TCGA.78.7146.01A | LUAD | -573.2269537 | 543.9776302 | -29.24932348 | 0.824943701 |
| TCGA.95.7948.01A | LUAD | -383.6531553 | 352.6402201 | -31.01293518 | 0.825089995 |
| TCGA.55.7570.01A | LUAD | -730.1954369 | -374.1125048 | -1104.307942 | 0.903517948 |
| TCGA.50.5072.01A | LUAD | 22.97378281 | 419.3750054 | 442.3487882 | 0.783872042 |
| TCGA.91.8499.01A | LUAD | -305.4578904 | 947.5139716 | 642.0560813 | 0.7653357 |
| TCGA.49.AAQV.01A | LUAD | -494.7729271 | 1266.176858 | 771.4039312 | 0.752978012 |
| TCGA.86.8671.01A | LUAD | 1474.790998 | 3145.709671 | 4620.500669 | 0.283625943 |
| TCGA.91.6829.01A | LUAD | 1184.400735 | 578.8822826 | 1763.283018 | 0.649540269 |
| TCGA.05.5428.01A | LUAD | 358.5967436 | 1153.398775 | 1511.995519 | 0.67713758 |
| TCGA.91.8497.01A | LUAD | 586.3768323 | 1999.337817 | 2585.714649 | 0.553246263 |
| TCGA.78.7145.01A | LUAD | 166.2073695 | 745.9164107 | 912.1237802 | 0.739225728 |
| TCGA.69.7973.01A | LUAD | -88.41047786 | 234.7007614 | 146.2902835 | 0.810107475 |
| TCGA.50.5936.11A | LUAD | 982.7135379 | 2303.122081 | 3285.835619 | 0.464868934 |
| TCGA.80.5611.01A | LUAD | -196.5199367 | 1869.893568 | 1673.373631 | 0.659517947 |
| TCGA.91.6849.01A | LUAD | 147.7455221 | 1614.185511 | 1761.931033 | 0.649691148 |
| TCGA.49.AAR9.01A | LUAD | -850.4182553 | -248.8799294 | -1099.298185 | 0.903202558 |

Supplementary Table 11 ESTIMATE

| | | | | | |
|---|---|---|---|---|---|
| TCGA.86.8669.01A | LUAD | -242.682983 | 1167.3522 | 924.6692168 | 0.737984286 |
| TCGA.86.8279.01A | LUAD | 349.4847857 | 377.9034621 | 727.3882479 | 0.757213893 |
| TCGA.75.6206.01A | LUAD | 297.7286714 | 894.1383752 | 1191.867047 | 0.710956114 |
| TCGA.75.6214.01A | LUAD | -959.2263788 | 62.53466031 | -896.6917185 | 0.890040075 |
| TCGA.49.6744.01A | LUAD | 1218.683355 | 2306.465748 | 3525.149103 | 0.43348653 |
| TCGA.64.1680.01A | LUAD | -674.2333633 | 497.524021 | -176.7093423 | 0.836983814 |
| TCGA.55.8091.01A | LUAD | 1113.325389 | 1769.694768 | 2883.020157 | 0.516377318 |
| TCGA.44.5645.11A | LUAD | 968.1573444 | 2202.073046 | 3170.230391 | 0.479825735 |
| TCGA.44.A47B.01A | LUAD | -131.616498 | 1178.129252 | 1046.512754 | 0.725797634 |
| TCGA.73.4676.01A | LUAD | -12.37048873 | 835.8547493 | 823.4842606 | 0.747925438 |
| TCGA.55.6982.01A | LUAD | 985.162416 | 1403.055777 | 2388.218193 | 0.577159756 |
| TCGA.4B.A93V.01A | LUAD | -798.2467781 | 801.4480291 | 3.201251009 | 0.822242026 |
| TCGA.62.A46Y.01A | LUAD | -140.8268613 | 1872.723989 | 1731.897128 | 0.653036255 |
| TCGA.78.7160.01A | LUAD | 727.128847 | 1962.819643 | 2689.94849 | 0.540436567 |
| TCGA.44.2656.01A | LUAD | 894.4073577 | 2404.067322 | 3298.474679 | 0.463225519 |
| TCGA.75.7025.01A | LUAD | 514.0415885 | 1638.428635 | 2152.470223 | 0.605068153 |
| TCGA.L4.A4E5.01A | LUAD | -580.8335285 | 293.9044333 | -286.9290952 | 0.845727472 |
| TCGA.50.5051.01A | LUAD | -600.0580355 | 82.64520465 | -517.4128309 | 0.863293499 |
| TCGA.64.1681.01A | LUAD | 311.1930496 | 1288.63206 | 1599.82511 | 0.66759464 |
| TCGA.78.7161.01A | LUAD | -312.0705043 | -221.3820633 | -533.4525676 | 0.8644794 |
| TCGA.05.4426.01A | LUAD | -337.8621116 | 1021.268371 | 683.4062592 | 0.76141496 |
| TCGA.50.5049.01A | LUAD | 1739.735334 | 3009.247103 | 4748.982437 | 0.265491413 |
| TCGA.44.6145.11A | LUAD | 754.564739 | 2291.066274 | 3045.631013 | 0.495791347 |
| TCGA.44.8120.01A | LUAD | 564.3336874 | 930.2383999 | 1494.572087 | 0.679017359 |
| TCGA.91.6836.11A | LUAD | 865.2714771 | 2622.328797 | 3487.600274 | 0.438446873 |
| TCGA.86.7713.01A | LUAD | -980.6310759 | -249.7098754 | -1230.340951 | 0.911291141 |
| TCGA.67.3773.01A | LUAD | 657.7917775 | 1925.755931 | 2583.547709 | 0.553511202 |
| TCGA.44.A479.01A | LUAD | 1005.638345 | 2490.174703 | 3495.813048 | 0.437363068 |
| TCGA.50.8460.01A | LUAD | 324.74812 | 1850.281611 | 2175.029732 | 0.602428331 |
| TCGA.05.4422.01A | LUAD | -508.6871901 | 1795.682572 | 1286.995382 | 0.701067314 |
| TCGA.05.4390.01A | LUAD | 245.7941672 | 516.9031976 | 762.6973648 | 0.753818394 |
| TCGA.91.6830.01A | LUAD | 826.1603509 | 1536.114618 | 2362.274969 | 0.580265429 |
| TCGA.50.5068.01A | LUAD | 762.979094 | 2369.830636 | 3132.80973 | 0.48463776 |
| TCGA.44.7669.01A | LUAD | -55.22368224 | 1120.749339 | 1065.525657 | 0.723874945 |
| TCGA.50.5066.01A | LUAD | 1095.637822 | 2380.585082 | 3476.222904 | 0.439947245 |
| TCGA.64.5815.01A | LUAD | 1565.631907 | 1768.544193 | 3334.1761 | 0.458574788 |
| TCGA.50.5939.01A | LUAD | 596.3458559 | 1643.142149 | 2239.488005 | 0.594849384 |
| TCGA.MP.A4TA.01A | LUAD | -282.5762958 | 969.7037169 | 687.1274211 | 0.761060749 |
| TCGA.97.A4M1.01A | LUAD | 108.921444 | 1461.27888 | 1570.200324 | 0.67082594 |
| TCGA.44.5645.01B | LUAD | 624.2387088 | 1370.370147 | 1994.608856 | 0.623353172 |
| TCGA.64.1677.01A | LUAD | -621.9629936 | 1269.768289 | 647.8052952 | 0.764792255 |
| TCGA.44.2662.01A | LUAD | 835.9600702 | 1844.317607 | 2680.277677 | 0.541630421 |
| TCGA.44.6775.01A | LUAD | 1461.007491 | 1682.38179 | 3143.389282 | 0.483278783 |
| TCGA.38.4632.01A | LUAD | 330.2279266 | 1875.67448 | 2205.902407 | 0.598805037 |
| TCGA.55.8092.01A | LUAD | 279.0931456 | 1880.814124 | 2159.90727 | 0.604198632 |
| TCGA.50.5946.02A | LUAD | 1056.174144 | 743.1054075 | 1799.279552 | 0.645513748 |
| TCGA.MP.A4TI.01A | LUAD | 1274.561118 | 2896.330558 | 4170.891677 | 0.346249747 |
| TCGA.05.4418.01A | LUAD | 103.8099162 | 1639.846792 | 1743.656708 | 0.651728009 |
| TCGA.MN.A4N4.01A | LUAD | 661.5042385 | 761.573072 | 1423.077311 | 0.686684147 |
| TCGA.55.8507.01A | LUAD | -192.859071 | 601.2623206 | 408.4032496 | 0.786956237 |
| TCGA.86.8668.01A | LUAD | 839.3099119 | 1455.330771 | 2294.640683 | 0.588322277 |
| TCGA.05.5425.01A | LUAD | 423.9665428 | 2143.631126 | 2567.597668 | 0.5554596 |
| TCGA.49.4510.01A | LUAD | -192.7135576 | 509.3925698 | 316.6790122 | 0.795192098 |
| TCGA.55.6979.01A | LUAD | 1096.583971 | 2657.62422 | 3754.208191 | 0.402947329 |
| TCGA.78.7158.01A | LUAD | -634.7372964 | 288.2514281 | -346.4858683 | 0.850360088 |
| TCGA.44.6775.01C | LUAD | 1142.660523 | 1366.751293 | 2509.411816 | 0.562541474 |
| TCGA.97.8547.01A | LUAD | 1027.205281 | 1135.687833 | 2162.893114 | 0.603849332 |
| TCGA.78.7153.01A | LUAD | -589.5992785 | 341.4932024 | -248.1060762 | 0.842672807 |
| TCGA.35.4122.01A | LUAD | 915.0429976 | 2414.205805 | 3329.248802 | 0.459217407 |
| TCGA.44.3918.01B | LUAD | 1086.108285 | 1888.476843 | 2974.585128 | 0.504820951 |
| TCGA.49.AARE.01A | LUAD | -74.44512934 | 914.5658249 | 840.1206955 | 0.746302228 |
| TCGA.75.5126.01A | LUAD | 783.787319 | 2396.412168 | 3180.199487 | 0.478541334 |
| TCGA.49.6761.01A | LUAD | -16.20263548 | 1490.182982 | 1473.980347 | 0.681233233 |
| TCGA.91.7771.01A | LUAD | 794.745577 | 2046.874728 | 2841.620304 | 0.521571874 |
| TCGA.69.7980.01A | LUAD | 292.2071897 | 1460.176819 | 1752.384008 | 0.650755846 |
| TCGA.69.8253.01A | LUAD | -499.5475392 | 729.4242369 | 229.8766977 | 0.80285329 |
| TCGA.95.7947.01A | LUAD | -703.348478 | 1008.597928 | 305.2494496 | 0.796208281 |
| TCGA.49.4490.11A | LUAD | 1177.171428 | 2276.916397 | 3454.087825 | 0.442862751 |

Supplementary Table 11 ESTIMATE

| | | | | | |
|---|---|---|---|---|---|
| TCGA.62.8394.01A | LUAD | -293.560866 | 698.7106059 | 405.1497399 | 0.787250817 |
| TCGA.55.8616.01A | LUAD | -205.007761 | 657.2274296 | 452.2196686 | 0.782971546 |
| TCGA.49.6743.01A | LUAD | 504.4246294 | 1082.884333 | 1587.308963 | 0.668961372 |
| TCGA.49.AAR4.01A | LUAD | 109.0433727 | 2269.111134 | 2378.154507 | 0.57836548 |
| TCGA.38.4625.01A | LUAD | 72.89951565 | 1372.625871 | 1445.525387 | 0.684285019 |
| TCGA.75.6205.01A | LUAD | 1368.826457 | 2832.986237 | 4201.812694 | 0.341988111 |
| TCGA.05.4417.01A | LUAD | 1429.978655 | 2010.904952 | 3440.883607 | 0.444599712 |
| TCGA.50.5045.01A | LUAD | 1529.035584 | 2588.346012 | 4117.381596 | 0.353607779 |
| TCGA.97.A4M2.01A | LUAD | 461.083637 | 2442.985266 | 2904.068903 | 0.513728949 |
| TCGA.97.A4M3.01A | LUAD | 51.82663092 | 185.3212596 | 237.1478906 | 0.802216518 |
| TCGA.44.2668.01A | LUAD | 1068.432342 | 2079.784029 | 3148.216371 | 0.482658341 |
| TCGA.71.8520.01A | LUAD | 522.6694483 | 585.5431311 | 1108.212579 | 0.719537692 |
| TCGA.75.5125.01A | LUAD | 400.1302671 | 1889.495842 | 2289.626109 | 0.588917333 |
| TCGA.49.4512.01A | LUAD | 882.3405671 | 1238.664396 | 2121.004963 | 0.608739001 |
| TCGA.86.7711.01A | LUAD | 698.6181136 | 1540.263732 | 2238.881846 | 0.594920904 |
| TCGA.44.2656.01B | LUAD | 1003.448348 | 1945.752788 | 2949.201136 | 0.508033877 |
| TCGA.L4.A4E6.01A | LUAD | 1071.712333 | 2635.301661 | 3707.013994 | 0.409277874 |
| TCGA.49.AAR0.01A | LUAD | 31.91303475 | 1647.720342 | 1679.633377 | 0.658826973 |
| TCGA.50.7109.01A | LUAD | 203.5896741 | 1160.302488 | 1363.892162 | 0.692973724 |
| TCGA.L9.A50W.01A | LUAD | -282.2638775 | 986.2922645 | 704.028387 | 0.759449119 |
| TCGA.49.6745.11A | LUAD | 808.8712415 | 2184.135975 | 2993.007216 | 0.502484821 |
| TCGA.80.5607.01A | LUAD | -89.72333283 | 1542.346829 | 1452.623496 | 0.683524864 |
| TCGA.55.8094.01A | LUAD | -1782.403888 | -753.8281985 | -2536.232086 | 0.973047826 |
| TCGA.05.4430.01A | LUAD | 1224.952515 | 1697.950384 | 2922.902899 | 0.511355082 |
| TCGA.91.A4BD.01A | LUAD | -484.9063117 | 1815.461764 | 1330.555452 | 0.696493403 |
| TCGA.78.7149.01A | LUAD | -82.61991477 | 173.6269209 | 91.00700611 | 0.81483839 |
| TCGA.55.7724.01A | LUAD | 811.8060819 | 1908.333024 | 2720.139106 | 0.536702564 |
| TCGA.50.6593.01A | LUAD | 909.2618999 | 1368.983521 | 2278.245421 | 0.59026664 |
| TCGA.86.8674.01A | LUAD | -914.3119991 | -369.0127889 | -1283.324788 | 0.914465995 |
| TCGA.49.4505.01A | LUAD | 623.1888556 | 1965.38742 | 2588.576276 | 0.552896302 |
| TCGA.05.5420.01A | LUAD | 775.1266605 | 2821.843453 | 3596.970113 | 0.423962145 |
| TCGA.55.6983.01A | LUAD | 522.612737 | 1759.723155 | 2282.335892 | 0.589781859 |
| TCGA.44.8119.01A | LUAD | 1007.275448 | 1169.671352 | 2176.946801 | 0.602203699 |
| TCGA.55.6971.11A | LUAD | 840.9089698 | 2179.026572 | 3019.935542 | 0.499063399 |
| TCGA.55.7994.01A | LUAD | -250.6200652 | 2058.595695 | 1807.97563 | 0.64453831 |
| TCGA.55.8508.01A | LUAD | 14.18784718 | 1067.502321 | 1081.690168 | 0.722235865 |
| TCGA.73.7499.01A | LUAD | 196.3250742 | 1965.995686 | 2162.32076 | 0.603916298 |
| TCGA.50.5933.01A | LUAD | 1496.74242 | 1726.278702 | 3223.021122 | 0.473012646 |
| TCGA.55.6970.11A | LUAD | 1227.873498 | 2910.076513 | 4137.950011 | 0.350782026 |
| TCGA.NJ.A4YI.01A | LUAD | 167.132818 | 1148.608338 | 1315.741156 | 0.698052144 |
| TCGA.55.7573.01A | LUAD | 277.9021473 | 1668.54842 | 1946.450567 | 0.628865149 |
| TCGA.75.7030.01A | LUAD | 1031.69262 | 1566.678475 | 2598.371095 | 0.551697712 |
| TCGA.91.A4BC.01A | LUAD | 1018.559009 | 2504.729638 | 3523.288647 | 0.433732615 |
| TCGA.78.7220.01A | LUAD | -698.5821996 | -137.9370666 | -836.5192662 | 0.885978775 |
| TCGA.05.4382.01A | LUAD | 1312.149004 | 1946.569444 | 3258.718448 | 0.468389483 |
| TCGA.75.7031.01A | LUAD | -422.7706687 | 1259.7709 | 837.0002311 | 0.74660703 |
| TCGA.86.8278.01A | LUAD | 535.9545207 | 1127.246981 | 1663.201502 | 0.660639596 |
| TCGA.NJ.A55R.01A | LUAD | -357.6176331 | 74.85486365 | -282.7627694 | 0.845400972 |
| TCGA.91.8496.01A | LUAD | 219.7954473 | 2050.249039 | 2270.044487 | 0.591237931 |
| TCGA.44.2655.11A | LUAD | 1153.917297 | 1740.362445 | 2894.279742 | 0.51496124 |
| TCGA.MP.A4TJ.01A | LUAD | 992.131862 | 2863.419673 | 3855.551535 | 0.389288362 |
| TCGA.69.7765.01A | LUAD | 1205.636777 | 1472.976356 | 2678.613133 | 0.541835797 |
| TCGA.64.5775.01A | LUAD | -8.198467066 | 702.8646126 | 694.6661455 | 0.760342455 |
| TCGA.44.7661.01A | LUAD | 978.4644964 | 2020.815877 | 2999.280373 | 0.501688475 |
| TCGA.49.4486.01A | LUAD | -1288.609672 | -85.91851392 | -1374.528186 | 0.919801349 |
| TCGA.75.6211.01A | LUAD | -40.28392379 | 341.5343534 | 301.2504296 | 0.796563298 |
| TCGA.05.4427.01A | LUAD | 204.7732668 | 1149.700328 | 1354.473595 | 0.693969819 |
| TCGA.MP.A4TE.01A | LUAD | -1104.219087 | -388.0084768 | -1492.227564 | 0.926442925 |
| TCGA.38.7271.01A | LUAD | 1297.403125 | 2934.393747 | 4231.796872 | 0.337848864 |
| TCGA.62.8399.01A | LUAD | 78.48360419 | 491.4213468 | 569.904951 | 0.772109316 |
| TCGA.49.AAR2.01A | LUAD | -639.8093845 | 1196.547827 | 556.7384429 | 0.773336078 |
| TCGA.86.7953.01A | LUAD | 187.0900728 | 1717.030391 | 1904.120464 | 0.633684117 |
| TCGA.69.8254.01A | LUAD | -352.175159 | 1473.101948 | 1120.926789 | 0.718240382 |
| TCGA.86.8055.01A | LUAD | 1331.996073 | 1290.343683 | 2622.339756 | 0.548759868 |
| TCGA.44.2665.11A | LUAD | 1275.526351 | 2062.870751 | 3338.397102 | 0.458024094 |
| TCGA.55.5899.01A | LUAD | 356.6191357 | 971.3961564 | 1328.015292 | 0.69676091 |
| TCGA.78.8655.01A | LUAD | 218.4743153 | 1644.077047 | 1862.551362 | 0.638392642 |
| TCGA.05.4420.01A | LUAD | -208.4109889 | 231.6620648 | 23.25107596 | 0.820563448 |

Supplementary Table 11 ESTIMATE

| | | | | | |
|---|---|---|---|---|---|
| TCGA.49.AARN.01A | LUAD | -162.8466558 | 989.2064438 | 826.3597879 | 0.747645192 |
| TCGA.49.6745.01A | LUAD | 797.9380343 | 1870.933783 | 2668.871817 | 0.543037062 |
| TCGA.38.4625.11A | LUAD | 970.7065016 | 2331.608731 | 3302.315233 | 0.462725829 |
| TCGA.55.8505.01A | LUAD | 256.772741 | 43.04507243 | 299.8178134 | 0.796690413 |
| TCGA.99.8028.01A | LUAD | 1415.747319 | 2945.363893 | 4361.111212 | 0.319923353 |
| TCGA.38.6178.01A | LUAD | 444.2536639 | 684.7710663 | 1129.02473 | 0.717412794 |
| TCGA.55.6987.01A | LUAD | 696.5260361 | 2861.596311 | 3558.122348 | 0.429119774 |
| TCGA.49.4494.01A | LUAD | -154.0290904 | 1244.7534 | 1090.724309 | 0.721318033 |
| TCGA.55.6543.01A | LUAD | 183.9910686 | 1306.004627 | 1489.995695 | 0.679510361 |
| TCGA.64.1678.01A | LUAD | -848.741849 | -168.0674218 | -1016.809271 | 0.897939367 |
| TCGA.91.6835.01A | LUAD | 985.7683612 | 2782.965802 | 3768.734163 | 0.400994936 |
| TCGA.S2.AA1A.01A | LUAD | 832.8755471 | 2331.531031 | 3164.406578 | 0.48057559 |
| TCGA.69.7761.01A | LUAD | 535.6976242 | 2247.453416 | 2783.15104 | 0.52887532 |
| TCGA.86.8672.01A | LUAD | 576.3464038 | 1734.657953 | 2311.004357 | 0.586378263 |
| TCGA.44.5644.01A | LUAD | -1093.164186 | -291.2873137 | -1384.451499 | 0.920371931 |
| TCGA.49.4506.01A | LUAD | -126.6100556 | 1678.352254 | 1551.742198 | 0.672832835 |
| TCGA.MP.A4SV.01A | LUAD | 101.3840022 | 1566.05936 | 1667.443362 | 0.660172038 |
| TCGA.53.7626.01A | LUAD | 814.7644789 | 2412.942463 | 3227.706942 | 0.472406523 |
| TCGA.91.6847.11A | LUAD | 133.7821611 | 1425.873971 | 1559.656132 | 0.671972981 |
| TCGA.MN.A4N1.01A | LUAD | -560.5819809 | -17.33271947 | -577.9147003 | 0.867741642 |
| TCGA.91.6835.11A | LUAD | 953.6948284 | 1313.104947 | 2266.799775 | 0.591621988 |
| TCGA.35.4123.01A | LUAD | 847.182137 | 2378.187976 | 3225.370113 | 0.472708826 |
| TCGA.55.6983.11A | LUAD | 251.8628752 | 2386.929247 | 2638.792122 | 0.546739365 |
| TCGA.55.8621.01A | LUAD | 1112.216415 | 2476.636715 | 3588.853129 | 0.425040942 |
| TCGA.69.A59K.01A | LUAD | -206.4149018 | 1151.38608 | 944.9711786 | 0.735969992 |
| TCGA.49.4501.01A | LUAD | 575.7998149 | 1587.114146 | 2162.913961 | 0.603846892 |
| TCGA.44.2655.01A | LUAD | -134.0125566 | 1249.81713 | 1115.804574 | 0.718763334 |
| TCGA.05.4434.01A | LUAD | 745.7372511 | 2222.167184 | 2967.904435 | 0.505667227 |
| TCGA.78.7148.01A | LUAD | -342.556046 | 754.7442605 | 412.1882146 | 0.786613313 |
| TCGA.44.6145.01A | LUAD | 837.075722 | 2238.380514 | 3075.456236 | 0.491984573 |
| TCGA.55.8615.01A | LUAD | -643.0119546 | 65.78255687 | -577.2293977 | 0.867691641 |
| TCGA.73.4676.11A | LUAD | 1045.968036 | 2612.223568 | 3658.191603 | 0.415806148 |
| TCGA.38.4626.01A | LUAD | 1165.654281 | 2164.829877 | 3330.484159 | 0.459056314 |
| TCGA.50.5930.01A | LUAD | 633.6766874 | 1556.254881 | 2189.931568 | 0.600680955 |
| TCGA.55.6981.01A | LUAD | -21.07658854 | 764.5156449 | 743.4390564 | 0.755672884 |
| TCGA.55.6984.11A | LUAD | 415.1475843 | 1921.3879 | 2336.535484 | 0.583338397 |
| TCGA.62.A46R.01A | LUAD | 100.7752284 | 1613.499503 | 1714.274732 | 0.654993093 |
| TCGA.95.8039.01A | LUAD | 130.8525023 | 1805.014178 | 1935.86668 | 0.63007233 |
| TCGA.55.6986.11A | LUAD | 574.2612276 | 1732.508876 | 2306.770103 | 0.58688162 |
| TCGA.97.7554.01A | LUAD | 1137.493429 | 1193.211452 | 2330.704882 | 0.584033342 |
| TCGA.67.6216.01A | LUAD | -159.2834579 | 1676.187731 | 1516.904273 | 0.676607185 |
| TCGA.MN.A4N5.01A | LUAD | -518.5315861 | 1230.146855 | 711.6152688 | 0.758724134 |
| TCGA.44.3398.11B | LUAD | 1413.761463 | 2493.963114 | 3907.724577 | 0.382222743 |
| TCGA.93.8067.01A | LUAD | -526.4603985 | 406.9186504 | -119.541748 | 0.832362317 |
| TCGA.97.8176.01A | LUAD | -262.4903179 | 883.0872356 | 620.5969178 | 0.76735931 |
| TCGA.55.A493.01A | LUAD | 213.0287066 | 2048.665368 | 2261.694074 | 0.592226046 |
| TCGA.62.A46S.01A | LUAD | -287.4262764 | 1015.55429 | 728.1280136 | 0.757142962 |
| TCGA.55.6968.01A | LUAD | -15.69330944 | 1487.927817 | 1472.234508 | 0.681420816 |
| TCGA.44.6147.01A | LUAD | 720.3137812 | 1271.363587 | 1991.677368 | 0.62368959 |
| TCGA.55.8514.01A | LUAD | -318.9976649 | 929.528536 | 610.5308711 | 0.768305921 |
| TCGA.55.7911.01A | LUAD | -95.97902631 | 2005.215046 | 1909.236019 | 0.633103045 |
| TCGA.35.3615.01A | LUAD | -143.5470409 | 758.2284665 | 614.6814256 | 0.767915806 |
| TCGA.73.4658.01A | LUAD | 1454.415256 | 2205.161026 | 3659.576283 | 0.415621288 |
| TCGA.44.6778.01A | LUAD | 794.244101 | 2842.08673 | 3636.330831 | 0.418722354 |
| TCGA.62.8397.01A | LUAD | -68.88693281 | 1169.597262 | 1100.710329 | 0.720302017 |
| TCGA.50.6595.01A | LUAD | 1188.268163 | 1482.46922 | 2670.737384 | 0.542807093 |
| TCGA.L9.A444.01A | LUAD | 679.6554119 | 2583.35977 | 3263.015182 | 0.467832143 |
| TCGA.64.5779.01A | LUAD | 403.2361328 | 1160.558355 | 1563.794488 | 0.671522985 |
| TCGA.44.7660.01A | LUAD | -642.5468034 | 582.5897522 | -59.95705129 | 0.82748304 |
| TCGA.44.2666.01A | LUAD | -544.5458718 | 282.0204254 | -262.5254463 | 0.843810549 |
| TCGA.49.4512.11A | LUAD | 1035.771431 | 2374.626382 | 3410.397812 | 0.448603601 |
| TCGA.44.6146.11A | LUAD | 1237.579533 | 2364.411305 | 3601.990838 | 0.423294558 |
| TCGA.44.6777.01A | LUAD | 2010.461826 | 2510.293683 | 4520.755509 | 0.297635223 |
| TCGA.73.4675.01A | LUAD | 321.6750621 | 722.003931 | 1043.678993 | 0.726083716 |
| TCGA.05.4410.01A | LUAD | 45.40116704 | 1813.844836 | 1859.246003 | 0.638766022 |
| TCGA.55.7903.01A | LUAD | -457.2998371 | 1243.519965 | 786.2201282 | 0.751545084 |
| TCGA.97.7553.01A | LUAD | 945.1266078 | 2751.712982 | 3696.83959 | 0.410640087 |
| TCGA.78.7147.01A | LUAD | -831.2927455 | 743.9476082 | -87.34513732 | 0.829733681 |

Supplementary Table 11 ESTIMATE

| | | | | | |
|---|---|---|---|---|---|
| TCGA.38.4627.11A | LUAD | 1523.214477 | 2534.17914 | 4057.393617 | 0.361830611 |
| TCGA.99.8033.01A | LUAD | -161.8609968 | 1466.954818 | 1305.093821 | 0.699170405 |
| TCGA.55.1594.01A | LUAD | -417.6336079 | 816.0986898 | 398.4650819 | 0.787855497 |
| TCGA.55.6980.01A | LUAD | 860.5576954 | 1887.658852 | 2748.216547 | 0.53322046 |
| TCGA.95.A4VK.01A | LUAD | -406.4096078 | 1156.797398 | 750.3877899 | 0.755004447 |
| TCGA.55.8207.01A | LUAD | 1078.023249 | 1556.932606 | 2634.955855 | 0.547210779 |
| TCGA.NJ.A55O.01A | LUAD | -138.3378432 | 1238.723978 | 1100.386135 | 0.720335026 |
| TCGA.95.7043.01A | LUAD | -505.5542251 | -343.2159381 | -848.7701632 | 0.886811255 |
| TCGA.44.7672.01A | LUAD | 1156.365235 | 2364.485816 | 3520.851051 | 0.434054989 |
| TCGA.44.6146.01B | LUAD | 242.6983876 | 151.3200104 | 394.018398 | 0.788257314 |
| TCGA.67.3771.01A | LUAD | 414.4044348 | 1495.258553 | 1909.662988 | 0.63305453 |
| TCGA.67.3772.01A | LUAD | 985.6333545 | 1561.056331 | 2546.689686 | 0.558009028 |
| TCGA.97.A4M5.01A | LUAD | 613.911438 | 1551.839388 | 2165.750826 | 0.603514912 |
| TCGA.53.A4EZ.01A | LUAD | -958.7949416 | 135.132023 | -823.6629186 | 0.885102072 |
| TCGA.99.8032.01A | LUAD | 588.3159025 | 774.6531674 | 1362.96907 | 0.693071407 |
| TCGA.73.4670.01A | LUAD | 143.798065 | 516.7744039 | 660.5724689 | 0.763583491 |
| TCGA.55.7576.01A | LUAD | 348.8522402 | 1207.082343 | 1555.934584 | 0.672377443 |
| TCGA.49.6743.11A | LUAD | 1267.179566 | 1958.113521 | 3225.293087 | 0.472718789 |
| TCGA.J2.A4AE.01A | LUAD | -434.9570622 | 1458.16394 | 1023.206878 | 0.728146738 |
| TCGA.93.7347.01A | LUAD | 1011.725901 | 2500.470617 | 3512.196518 | 0.435199111 |
| TCGA.75.7027.01A | LUAD | 422.3984942 | 264.5860008 | 686.984495 | 0.761074358 |
| TCGA.62.A472.01A | LUAD | -486.2068665 | 1308.927394 | 822.7205276 | 0.747999848 |
| TCGA.78.7154.01A | LUAD | -366.5986485 | 222.8158301 | -143.7828184 | 0.834329171 |
| TCGA.50.5044.01A | LUAD | 308.784037 | 1370.503033 | 1679.28707 | 0.658865215 |
| TCGA.95.A4VP.01A | LUAD | 280.0026381 | 1468.37918 | 1748.381818 | 0.651201796 |
| TCGA.44.2661.01A | LUAD | 1148.343571 | 2807.326746 | 3955.670316 | 0.375709837 |
| TCGA.49.AARO.01A | LUAD | 753.0252041 | 2115.834111 | 2868.859315 | 0.518156274 |
| TCGA.91.6831.01A | LUAD | 717.3183687 | 1110.343453 | 1827.661822 | 0.642326235 |
| TCGA.55.6642.01A | LUAD | 1398.052556 | 1444.187861 | 2842.240418 | 0.521494208 |
| TCGA.86.8056.01A | LUAD | 77.93707321 | 1197.574371 | 1275.511444 | 0.702268388 |
| TCGA.L9.A5IP.01A | LUAD | -610.8722027 | 845.8397035 | 234.9675008 | 0.80240756 |
| TCGA.97.7546.01A | LUAD | 824.6954585 | 1652.808319 | 2477.503778 | 0.566407652 |
| TCGA.97.7547.01A | LUAD | 191.9493309 | 935.4232201 | 1127.372551 | 0.717581725 |
| TCGA.44.6776.11A | LUAD | 512.1426317 | 1622.792466 | 2134.935097 | 0.60711546 |
| TCGA.55.7914.01A | LUAD | -25.31207493 | 1109.535126 | 1084.223051 | 0.721978663 |
| TCGA.78.7143.01A | LUAD | -683.0128901 | 1109.635036 | 426.6221461 | 0.785303345 |
| TCGA.97.A4M0.01A | LUAD | -237.468361 | 1909.359399 | 1671.891038 | 0.65968152 |
| TCGA.NJ.A55A.01A | LUAD | 383.6607558 | 1867.949518 | 2251.610273 | 0.593418088 |
| TCGA.55.8205.01A | LUAD | 767.1465095 | 2567.912448 | 3335.058958 | 0.458459621 |
| TCGA.05.4415.01A | LUAD | -939.4495963 | 450.6554554 | -488.7941409 | 0.861165688 |
| TCGA.97.7941.01A | LUAD | 175.2878977 | 1100.039362 | 1275.327259 | 0.702287635 |
| TCGA.86.8585.01A | LUAD | 26.93959075 | 2015.071332 | 2042.010922 | 0.617897328 |
| TCGA.55.8203.01A | LUAD | 108.6948926 | 1399.025178 | 1507.72007 | 0.67759926 |
| TCGA.71.6725.01A | LUAD | -783.375389 | 302.2425522 | -481.1328369 | 0.860593487 |
| TCGA.55.7816.01A | LUAD | 1620.581181 | 2016.339897 | 3636.921079 | 0.418643672 |
| TCGA.55.6978.01A | LUAD | 1511.825199 | 2500.419141 | 4012.24434 | 0.368000963 |
| TCGA.55.1592.01A | LUAD | 77.74395682 | 1012.641564 | 1090.385521 | 0.721352475 |
| TCGA.97.8175.01A | LUAD | -125.1579615 | 1489.152398 | 1363.994437 | 0.6929629 |
| TCGA.86.7954.01A | LUAD | 487.6212824 | 2081.458316 | 2569.079599 | 0.555278701 |
| TCGA.78.8662.01A | LUAD | -823.3676256 | -226.4232262 | -1049.790852 | 0.900059591 |
| TCGA.MP.A4TF.01A | LUAD | -1108.176669 | 525.7025411 | -582.4741274 | 0.868074086 |
| TCGA.MP.A4T7.01A | LUAD | -524.7263639 | 1116.78992 | 592.063556 | 0.770038225 |
| TCGA.44.5645.01A | LUAD | 287.3621728 | 1932.126278 | 2219.488451 | 0.597206644 |
| TCGA.55.8506.01A | LUAD | 73.0111234 | 1110.138618 | 1183.149741 | 0.711855393 |
| TCGA.50.6591.01A | LUAD | -986.3654788 | -1178.106219 | -2164.471698 | 0.959021558 |
| TCGA.91.6831.11A | LUAD | 1145.82886 | 2334.229146 | 3480.058005 | 0.439441634 |
| TCGA.78.7159.01A | LUAD | 60.67129942 | 613.1892874 | 673.8605868 | 0.762322557 |
| TCGA.69.7763.01A | LUAD | 774.2320995 | 925.257112 | 1699.489211 | 0.656631536 |
| TCGA.55.8085.01A | LUAD | -71.01693063 | 1720.618288 | 1649.601357 | 0.66213694 |
| TCGA.62.A471.01A | LUAD | -1295.105033 | 138.093562 | -1157.011471 | 0.906806256 |
| TCGA.91.6840.01A | LUAD | -214.0286104 | 1039.843765 | 825.815155 | 0.747698282 |
| TCGA.44.3917.01B | LUAD | 36.7521958 | 872.4654948 | 909.2176906 | 0.739512944 |
| TCGA.73.7498.01A | LUAD | 381.360467 | 1083.421991 | 1464.782458 | 0.682221006 |
| TCGA.55.8206.01A | LUAD | 475.4549736 | 2203.368052 | 2678.823025 | 0.541809902 |
| TCGA.75.6207.01A | LUAD | -493.6510097 | 499.2956175 | 5.644607761 | 0.822037848 |
| TCGA.97.8179.01A | LUAD | 8.190933966 | 514.6439442 | 522.8348782 | 0.776481666 |
| TCGA.91.6828.11A | LUAD | 399.7832143 | 1903.107502 | 2302.890716 | 0.587342592 |
| TCGA.50.8459.01A | LUAD | 1834.756837 | 2277.768706 | 4112.525542 | 0.354274449 |

Supplementary Table 11 ESTIMATE

| | | | | | |
|---|---|---|---|---|---|
| TCGA.50.6590.01A | LUAD | 465.2981159 | 2099.359564 | 2564.65768 | 0.555818406 |
| TCGA.49.6742.01A | LUAD | -827.5730074 | -35.09101414 | -862.6640215 | 0.887751908 |
| TCGA.64.1679.01A | LUAD | 1178.275163 | 1150.135227 | 2328.41039 | 0.584306703 |
| TCGA.99.7458.01A | LUAD | 885.3154186 | 1986.694965 | 2872.010384 | 0.517760615 |
| TCGA.97.A4M7.01A | LUAD | 493.3665307 | 2085.794895 | 2579.161425 | 0.554047317 |
| TCGA.MP.A4TH.01A | LUAD | 201.6893767 | 2133.657684 | 2335.347061 | 0.583480079 |
| TCGA.44.6144.11A | LUAD | 885.2710346 | 2429.326527 | 3314.597562 | 0.461126801 |
| TCGA.75.5122.01A | LUAD | 728.7034807 | 3353.364305 | 4082.067786 | 0.358451768 |
| TCGA.50.5930.11A | LUAD | 756.1768779 | 2126.81028 | 2882.987158 | 0.516381466 |
| TCGA.50.5066.02A | LUAD | 34.60317591 | 766.6404398 | 801.2436157 | 0.750088479 |
| TCGA.62.A46P.01A | LUAD | -442.8865079 | 333.6390616 | -109.2474464 | 0.831523876 |
| TCGA.38.4626.11A | LUAD | 757.1751253 | 2635.74452 | 3392.919645 | 0.45089507 |
| TCGA.50.5935.11A | LUAD | 1143.661213 | 2003.534931 | 3147.196143 | 0.482789494 |
| TCGA.38.4632.11A | LUAD | 1026.286214 | 2872.223968 | 3898.510182 | 0.383472259 |
| TCGA.75.6212.01A | LUAD | 249.1361416 | 1844.380933 | 2093.517075 | 0.61193521 |
| TCGA.44.6146.01A | LUAD | -902.4875781 | -91.87711665 | -994.3646948 | 0.896484478 |
| TCGA.55.7913.01B | LUAD | -1360.245441 | 78.01947332 | -1282.225968 | 0.914400714 |
| TCGA.78.7166.01A | LUAD | -901.5097656 | 577.3185838 | -324.1911818 | 0.84863349 |
| TCGA.93.A4JN.01A | LUAD | 278.9092 | 1106.381718 | 1385.290918 | 0.690705701 |
| TCGA.55.6985.01A | LUAD | 664.3673376 | 1790.511644 | 2454.878981 | 0.569141491 |
| TCGA.55.6975.11A | LUAD | 877.2594056 | 2163.415862 | 3040.675267 | 0.49642296 |
| TCGA.38.4627.01A | LUAD | 2115.229895 | 1815.169828 | 3930.399723 | 0.379144911 |
| TCGA.55.6975.01A | LUAD | 954.2914409 | 664.2019547 | 1618.493396 | 0.665551923 |
| TCGA.62.A46V.01A | LUAD | 251.3542183 | 521.1674548 | 772.521673 | 0.752870035 |
| TCGA.55.6984.01A | LUAD | -769.8877058 | 710.7281065 | -59.15959924 | 0.827417308 |
| TCGA.50.6594.01A | LUAD | -142.6641643 | 669.6071742 | 526.9430099 | 0.776101534 |
| TCGA.49.AAR3.01A | LUAD | 758.4690412 | 2378.518182 | 3136.987223 | 0.484101288 |
| TCGA.49.6744.11A | LUAD | 1236.770366 | 1886.461589 | 3123.231955 | 0.485867048 |
| TCGA.86.7955.01A | LUAD | -1684.839033 | -712.7599401 | -2397.598973 | 0.968153964 |
| TCGA.55.A4DG.01A | LUAD | -386.785154 | 1223.28272 | 836.4975659 | 0.746656115 |
| TCGA.44.6148.01A | LUAD | 515.1084695 | 1111.817296 | 1626.925765 | 0.664627599 |
| TCGA.93.A4JP.01A | LUAD | 497.2463587 | 1447.065676 | 1944.312035 | 0.629109189 |
| TCGA.78.7150.01A | LUAD | 85.07254149 | -78.91541151 | 6.157129975 | 0.821995006 |
| TCGA.97.8177.01A | LUAD | 1084.621147 | 1993.923353 | 3078.5445 | 0.491589859 |
| TCGA.50.5933.11A | LUAD | 851.0964744 | 2162.484685 | 3013.58116 | 0.49987147 |
| TCGA.75.6203.01A | LUAD | 872.9380267 | 2476.808743 | 3349.74677 | 0.456542486 |
| TCGA.50.5946.01A | LUAD | -1004.656471 | -342.6938577 | -1347.350329 | 0.918228658 |
| TCGA.50.5055.01A | LUAD | 1393.758349 | 2864.183335 | 4257.941684 | 0.334234288 |
| TCGA.78.8648.01A | LUAD | 2008.209762 | 2800.754192 | 4808.963954 | 0.25699261 |
| TCGA.44.6778.11A | LUAD | 1243.961354 | 2793.234494 | 4037.195847 | 0.364592931 |
| TCGA.78.8640.01A | LUAD | -711.2273073 | 920.3026796 | 209.0753723 | 0.804669906 |
| TCGA.50.5935.01A | LUAD | 198.5927191 | 1323.862575 | 1522.455294 | 0.676006968 |
| TCGA.78.7167.01A | LUAD | -626.7462152 | -135.7306508 | -762.476866 | 0.880886512 |
| TCGA.69.7978.01A | LUAD | 1027.968187 | 2576.911466 | 3604.879653 | 0.422910339 |
| TCGA.86.8054.01A | LUAD | -542.9689784 | -249.0143691 | -791.9833475 | 0.882928324 |
| TCGA.69.7974.01A | LUAD | 576.4229883 | 1922.647416 | 2499.070404 | 0.56379586 |
| TCGA.44.3398.01A | LUAD | 1365.785957 | 2159.161015 | 3524.946971 | 0.433513268 |
| TCGA.NJ.A4YG.01A | LUAD | 290.4779983 | 1390.3013 | 1680.779298 | 0.658700422 |
| TCGA.78.7537.01A | LUAD | 116.4412626 | 585.0930544 | 701.534317 | 0.759687241 |
| TCGA.38.4629.01A | LUAD | 1056.183982 | 2146.157839 | 3202.34182 | 0.475684891 |
| TCGA.55.8510.01A | LUAD | 735.5401808 | 2178.182036 | 2913.722217 | 0.512512719 |
| TCGA.69.7760.01A | LUAD | -543.4915596 | -275.6349626 | -819.1265222 | 0.884791972 |
| TCGA.55.7284.01B | LUAD | 434.694765 | 1525.225384 | 1959.920149 | 0.627326637 |
| TCGA.55.A4DF.01A | LUAD | -332.5603485 | 1593.965342 | 1261.404994 | 0.703741012 |
| TCGA.05.4402.01A | LUAD | 402.974213 | 1206.907839 | 1609.882052 | 0.666494813 |
| TCGA.86.A456.01A | LUAD | 331.6749702 | 1571.69947 | 1903.37444 | 0.633768828 |
| TCGA.49.AARR.01A | LUAD | 571.2955858 | 1759.089153 | 2330.384739 | 0.584071487 |
| TCGA.44.7667.01A | LUAD | -458.1086174 | 119.3486979 | -338.7599194 | 0.849762787 |
| TCGA.86.A4P8.01A | LUAD | 1066.469291 | 2865.896986 | 3932.366277 | 0.378877781 |
| TCGA.50.8457.01A | LUAD | 792.3155001 | 2087.796115 | 2880.111615 | 0.516742886 |
| TCGA.80.5608.01A | LUAD | -656.8215238 | 666.9422272 | 10.1207034 | 0.821663531 |
| TCGA.86.6851.01A | LUAD | 358.448587 | 2620.853586 | 2979.302173 | 0.504223127 |
| TCGA.55.6970.01A | LUAD | 95.21254631 | 1539.248834 | 1634.461381 | 0.663800712 |
| TCGA.05.4384.01A | LUAD | 117.2098607 | 1137.31288 | 1254.522741 | 0.704458381 |
| TCGA.44.7671.01A | LUAD | 173.5045994 | 44.62093419 | 218.1255336 | 0.803880461 |
| TCGA.55.8511.01A | LUAD | 696.0080413 | 1750.94489 | 2446.952931 | 0.570097742 |
| TCGA.MP.A4TC.01A | LUAD | 1028.80696 | 1539.995031 | 2568.801991 | 0.555312591 |
| TCGA.91.6828.01A | LUAD | 624.7989933 | 1965.396098 | 2590.195091 | 0.552698287 |

Supplementary Table 11 ESTIMATE

| | | | | | |
|---|---|---|---|---|---|
| TCGA.55.8302.01A | LUAD | -300.0141436 | 1141.190393 | 841.1762489 | 0.746199088 |
| TCGA.55.8087.01A | LUAD | -197.9233529 | 626.1655597 | 428.2422069 | 0.785156094 |
| TCGA.L9.A743.01A | LUAD | 766.3265461 | 2351.306987 | 3117.633533 | 0.48658515 |
| TCGA.38.4631.01A | LUAD | -525.7865049 | 726.7943439 | 201.0078389 | 0.80537244 |
| TCGA.NJ.A4YF.01A | LUAD | -670.4095216 | 210.7974333 | -459.6120883 | 0.858980343 |
| TCGA.55.7815.01A | LUAD | 1021.712296 | 1605.755377 | 2627.467673 | 0.548130455 |
| TCGA.55.8620.01A | LUAD | -905.2203658 | 877.1379714 | -28.08239445 | 0.824846872 |
| TCGA.50.5941.01A | LUAD | 727.2085389 | 2461.31488 | 3188.523419 | 0.477468107 |
| TCGA.78.7539.01A | LUAD | -102.2923313 | 1911.086904 | 1808.794573 | 0.644446395 |
| TCGA.55.8301.01A | LUAD | 819.3228037 | 2489.616887 | 3308.939691 | 0.461863582 |
| TCGA.50.5939.11A | LUAD | 1404.221985 | 2055.237255 | 3459.45924 | 0.442155688 |
| TCGA.73.A9RS.01A | LUAD | -954.6882452 | 211.174778 | -743.5134672 | 0.879565543 |
| TCGA.44.2665.01A | LUAD | 1567.701384 | 1579.25655 | 3146.957934 | 0.482820115 |
| TCGA.L9.A8F4.01A | LUAD | 154.2987119 | 1924.366047 | 2078.664759 | 0.613658051 |
| TCGA.55.7725.01A | LUAD | -140.2362761 | 1945.312921 | 1805.076645 | 0.644863605 |
| TCGA.55.A57B.01A | LUAD | 384.9103626 | 1306.51652 | 1691.426883 | 0.657523652 |
| TCGA.86.8673.01A | LUAD | 61.49926678 | 920.8764221 | 982.3756889 | 0.732241739 |
| TCGA.86.8074.01A | LUAD | 762.9240876 | 1214.821834 | 1977.745922 | 0.625286785 |
| TCGA.86.A4D0.01A | LUAD | -1040.330227 | 168.229986 | -872.1002407 | 0.888388661 |
| TCGA.MP.A4T8.01A | LUAD | 24.09483471 | 37.69030113 | 61.78513584 | 0.817317419 |
| TCGA.93.A4JQ.01A | LUAD | 959.1608899 | 2185.658792 | 3144.819682 | 0.483094954 |
| TCGA.44.2666.01B | LUAD | -170.9495638 | -78.85217277 | -249.8017365 | 0.842806797 |
| TCGA.97.8552.01A | LUAD | 134.2164035 | 1788.477579 | 1922.693983 | 0.631572661 |
| TCGA.69.7764.01A | LUAD | -273.9905144 | 682.442291 | 408.4517765 | 0.786951842 |
| TCGA.86.7701.01A | LUAD | 669.0074716 | 1987.828989 | 2656.836461 | 0.544519686 |
| TCGA.62.A470.01A | LUAD | -542.8998652 | 410.0125398 | -132.8873254 | 0.833446446 |
| TCGA.55.7727.01A | LUAD | -234.7287203 | 1354.468676 | 1119.739956 | 0.718361588 |
| TCGA.L9.A7SV.01A | LUAD | -944.9490331 | -38.16352179 | -983.1125549 | 0.895751436 |
| TCGA.97.7938.01A | LUAD | 99.62597345 | 696.8722935 | 796.4982669 | 0.75054896 |
| TCGA.67.6215.01A | LUAD | -662.062044 | 938.4651192 | 276.4030752 | 0.79876299 |
| TCGA.55.7907.01A | LUAD | 364.2131698 | 1537.938632 | 1902.151801 | 0.633907641 |
| TCGA.MP.A4T9.01A | LUAD | 475.9147958 | 1469.993669 | 1945.908465 | 0.628927018 |
| TCGA.97.7552.01A | LUAD | 488.0856585 | 2645.32015 | 3133.405809 | 0.484561223 |
| TCGA.05.5429.01A | LUAD | -211.3783935 | 354.2333914 | 142.854998 | 0.81040301 |
| TCGA.91.6847.01A | LUAD | -1785.965664 | -657.2989556 | -2443.26462 | 0.969810381 |
| TCGA.44.A4SS.01A | LUAD | 597.5981406 | 1993.536899 | 2591.13504 | 0.552583297 |
| TCGA.55.8097.01A | LUAD | 101.0994868 | 1052.850603 | 1153.95009 | 0.714859134 |
| TCGA.97.8171.01A | LUAD | -1090.690825 | -271.7597542 | -1362.450579 | 0.919104264 |
| TCGA.L9.A443.01A | LUAD | 293.8930028 | 886.9248074 | 1180.81781 | 0.712095758 |
| TCGA.55.8089.01A | LUAD | 658.2013365 | 2799.055642 | 3457.256978 | 0.442445615 |
| TCGA.05.4424.01A | LUAD | 661.5162732 | 1484.403593 | 2145.919866 | 0.605833407 |
| TCGA.62.8395.01A | LUAD | 402.428065 | 504.2104243 | 906.6384893 | 0.739767741 |
| TCGA.53.7624.01A | LUAD | -870.1662874 | 384.7940128 | -485.3722746 | 0.860910253 |
| TCGA.64.1676.01A | LUAD | 746.43065 | 1924.310289 | 2670.740939 | 0.542806654 |
| TCGA.55.1596.01A | LUAD | -575.0076303 | 471.7077769 | -103.2998534 | 0.831038596 |
| TCGA.97.8174.01A | LUAD | 813.6391992 | 1465.234368 | 2278.873567 | 0.590192209 |
| TCGA.93.7348.01A | LUAD | 591.5705442 | 1131.067989 | 1722.638534 | 0.6540649 |
| TCGA.38.4630.01A | LUAD | -315.7072648 | 12.40691172 | -303.3003531 | 0.847007365 |
| TCGA.05.5423.01A | LUAD | 322.265052 | 2117.278372 | 2439.543424 | 0.570990976 |
| TCGA.49.6767.01A | LUAD | -243.1171804 | 1399.65858 | 1156.5414 | 0.714593098 |
| TCGA.05.4397.01A | LUAD | -527.6893013 | 702.7874773 | 175.098176 | 0.807621052 |
| TCGA.55.7910.01A | LUAD | -413.9619182 | 396.4798476 | -17.48207058 | 0.823966177 |
| TCGA.J2.A4AG.01A | LUAD | 514.9397879 | 1831.114581 | 2346.054369 | 0.582202931 |
| TCGA.50.5936.01A | LUAD | 324.9714817 | 935.9977348 | 1260.969217 | 0.703786457 |
| TCGA.86.8073.01A | LUAD | 449.0958455 | 888.1768559 | 1337.272701 | 0.695785537 |
| TCGA.44.4112.01B | LUAD | 976.8353253 | 974.1418637 | 1950.977189 | 0.628348387 |
| TCGA.05.5715.01A | LUAD | 620.2022649 | 1205.7725 | 1825.974765 | 0.642516014 |
| TCGA.44.5643.01A | LUAD | -985.4241362 | 907.3556684 | -78.06846783 | 0.828972862 |
| TCGA.75.5146.01A | LUAD | 51.84695856 | 1438.48491 | 1490.331868 | 0.679474156 |
| TCGA.NJ.A4YQ.01A | LUAD | 52.05897394 | 2386.816115 | 2438.875089 | 0.571071512 |
| TCGA.44.2657.01A | LUAD | 833.3783339 | 2684.35395 | 3517.732284 | 0.434467368 |
| TCGA.55.7995.01A | LUAD | 62.29420721 | 2337.55044 | 2399.844647 | 0.57576523 |
| TCGA.44.2662.01B | LUAD | 1315.372999 | 1582.50953 | 2897.882529 | 0.514507833 |
| TCGA.49.4514.01A | LUAD | -590.727848 | 1132.364272 | 541.6364235 | 0.774739621 |
| TCGA.55.6712.01A | LUAD | 469.7193273 | 2090.791698 | 2560.511025 | 0.556324302 |
| TCGA.44.4112.01A | LUAD | 744.8978032 | 1040.958824 | 1785.856627 | 0.647017331 |
| TCGA.55.6972.01A | LUAD | -1753.183154 | -678.382829 | -2431.565983 | 0.969390188 |
| TCGA.44.3919.01A | LUAD | 593.2535406 | 2025.215712 | 2618.469253 | 0.549234738 |

Supplementary Table 11 ESTIMATE

| | | | | | |
|---|---|---|---|---|---|
| TCGA.J2.8192.01A | LUAD | 1609.400138 | 1914.999698 | 3524.399836 | 0.43358564 |
| TCGA.55.6968.11A | LUAD | 701.6435264 | 2596.729738 | 3298.373264 | 0.463238712 |
| TCGA.44.A47A.01A | LUAD | -54.61505838 | 1727.907678 | 1673.29262 | 0.659526886 |
| TCGA.75.5147.01A | LUAD | 337.1816027 | 1551.765143 | 1888.946746 | 0.635405585 |
| TCGA.86.A4JF.01A | LUAD | -198.3678013 | 1473.040083 | 1274.672282 | 0.702356076 |
| TCGA.69.8453.01A | LUAD | 596.026923 | 2476.455651 | 3072.482574 | 0.492364544 |
| TCGA.55.6985.11A | LUAD | 1049.554176 | 2405.716754 | 3455.270929 | 0.442707038 |
| TCGA.73.4662.01A | LUAD | 213.6672811 | 1706.832671 | 1920.499952 | 0.631822326 |
| TCGA.50.6595.11A | LUAD | 1037.0623 | 2399.348914 | 3436.411214 | 0.445187658 |
| TCGA.95.7039.01A | LUAD | 146.9333873 | 657.8538838 | 804.7872711 | 0.749744371 |
| TCGA.NJ.A4YP.01A | LUAD | 848.3756771 | 1027.960915 | 1876.336592 | 0.636833817 |
| TCGA.62.A46O.01A | LUAD | -1579.784434 | -949.8531804 | -2529.637614 | 0.972824148 |
| TCGA.73.4668.01A | LUAD | 140.6827801 | 455.4840457 | 596.1668258 | 0.769653811 |
| TCGA.05.4433.01A | LUAD | -151.6717812 | 1825.652466 | 1673.980685 | 0.659450963 |
| TCGA.95.A4VN.01A | LUAD | 303.1704756 | 2132.403514 | 2435.57399 | 0.571469223 |
| TCGA.78.7156.01A | LUAD | -691.8954317 | 49.26135408 | -642.6340776 | 0.872424088 |
| TCGA.38.A44F.01A | LUAD | 633.7844992 | 2267.909976 | 2901.694475 | 0.514027947 |
| TCGA.05.4396.01A | LUAD | -571.0948524 | 14.19836953 | -556.8964829 | 0.866204107 |
| TCGA.55.8204.01A | LUAD | 58.18535029 | 1209.917984 | 1268.103335 | 0.703042124 |
| TCGA.55.7281.01A | LUAD | 791.8978183 | 1947.331938 | 2739.229757 | 0.534335971 |
| TCGA.05.4405.01A | LUAD | 956.8865632 | 1120.785968 | 2077.672532 | 0.613773044 |
| TCGA.55.6982.11A | LUAD | 1017.675166 | 2606.249741 | 3623.924907 | 0.420375376 |
| TCGA.55.8614.01A | LUAD | 140.2822733 | 600.0791035 | 740.3613768 | 0.755968692 |
| TCGA.44.6148.11A | LUAD | 1096.524554 | 2015.925019 | 3112.449573 | 0.487249796 |
| TCGA.78.7152.01A | LUAD | -55.37040438 | 1360.75645 | 1305.386045 | 0.699139736 |
| TCGA.64.5781.01A | LUAD | 297.3465569 | 1668.134212 | 1965.480769 | 0.62669078 |
| TCGA.55.7227.01A | LUAD | 857.618444 | 1917.261371 | 2774.879815 | 0.529905353 |
| TCGA.MP.A4TD.01A | LUAD | 827.0997393 | 1214.705173 | 2041.804912 | 0.617921104 |
| TCGA.91.6849.11A | LUAD | 803.3060891 | 1691.665977 | 2494.972066 | 0.564292618 |
| TCGA.86.8281.01A | LUAD | -200.3885284 | 525.833709 | 325.4451807 | 0.794411196 |
| TCGA.55.8513.01A | LUAD | 619.6908484 | 2089.965015 | 2709.655863 | 0.53800034 |
| TCGA.78.7542.01A | LUAD | -1236.355623 | 613.7979224 | -622.557701 | 0.870979967 |
| TCGA.50.6673.01A | LUAD | 392.4705902 | 914.9768494 | 1307.44744 | 0.698923357 |
| TCGA.44.2662.11A | LUAD | 1483.884749 | 2502.175984 | 3986.060733 | 0.371571973 |
| TCGA.O1.A52J.01A | LUAD | -150.0242934 | 1571.11555 | 1421.091256 | 0.686896047 |
| TCGA.05.4425.01A | LUAD | 605.4965497 | 1919.444227 | 2524.940777 | 0.560655418 |
| TCGA.73.4659.01A | LUAD | 562.070077 | 1003.768838 | 1565.838915 | 0.671300587 |
| TCGA.35.5375.01A | LUAD | 89.65012656 | 1408.288032 | 1497.938159 | 0.678654546 |
| TCGA.44.2668.01B | LUAD | 917.4128864 | 2381.016174 | 3298.429061 | 0.463231454 |
| TCGA.91.6829.11A | LUAD | 501.4333525 | 2561.986419 | 3063.419772 | 0.493521999 |
| TCGA.86.6562.01A | LUAD | 852.4454225 | 923.5569114 | 1776.002334 | 0.648119571 |
| TCGA.86.8358.01A | LUAD | -501.0011219 | 193.2124553 | -307.7886667 | 0.847357404 |
| TCGA.50.5932.11A | LUAD | 1227.730384 | 2073.42685 | 3301.157234 | 0.46287651 |
| TCGA.44.6776.01A | LUAD | -672.6866412 | -37.15359771 | -709.8402389 | 0.877203115 |
| TCGA.95.7944.01A | LUAD | 175.4735327 | 2135.160217 | 2310.633749 | 0.586422329 |
| TCGA.MP.A4SW.01A | LUAD | 91.55113295 | 1649.855857 | 1741.40699 | 0.651978439 |
| TCGA.49.4487.01A | LUAD | 680.9618334 | 2145.412281 | 2826.374115 | 0.523480013 |
| TCGA.05.4403.01A | LUAD | 293.7947395 | 1625.296569 | 1919.091309 | 0.631982584 |
| TCGA.55.8512.01A | LUAD | -465.8161354 | 604.618274 | 138.8021386 | 0.81075141 |
| TCGA.44.7659.01A | LUAD | -447.8394084 | 929.2000797 | 481.3606713 | 0.780303502 |
| TCGA.55.6981.11A | LUAD | 952.5377431 | 2098.372136 | 3050.909879 | 0.495118264 |
| TCGA.91.6848.01A | LUAD | 1067.623555 | 1977.391564 | 3045.015118 | 0.495869858 |
| TCGA.55.6986.01A | LUAD | -216.1926607 | 1165.300791 | 949.1081307 | 0.735558735 |
| TCGA.55.6978.11A | LUAD | 660.2949794 | 2464.100578 | 3124.395557 | 0.485717754 |
| TCGA.50.5932.01A | LUAD | -525.0460042 | 232.5951374 | -292.4508668 | 0.846159706 |
| TCGA.55.6971.01A | LUAD | 697.9939046 | 2585.308115 | 3283.30202 | 0.465198177 |
| TCGA.55.7728.01A | LUAD | -95.10880418 | 2637.880657 | 2542.771853 | 0.558486168 |
| TCGA.44.2661.11A | LUAD | 1633.3693 | 2310.165209 | 3943.53451 | 0.377360126 |
| TCGA.44.6779.01A | LUAD | 745.2427924 | 2204.051132 | 2949.293924 | 0.508022145 |
| TCGA.MP.A4TK.01A | LUAD | 1345.185723 | 1831.171779 | 3176.357501 | 0.47903645 |
| TCGA.86.A4P7.01A | LUAD | 634.1574229 | 1931.693288 | 2565.850711 | 0.555672817 |
| TCGA.44.2657.11A | LUAD | 860.7186962 | 2954.506085 | 3815.224781 | 0.394734055 |
| TCGA.78.7162.01A | LUAD | 407.9172194 | 1456.169328 | 1864.086547 | 0.638219174 |
| TCGA.69.8255.01A | LUAD | -817.4323534 | 1758.159571 | 940.7272174 | 0.736391605 |
| TCGA.05.4432.01A | LUAD | -20.02818822 | 1278.526509 | 1258.498321 | 0.704044076 |
| TCGA.55.A492.01A | LUAD | -548.7865086 | 492.4370281 | -56.34948048 | 0.827185586 |
| TCGA.05.4244.01A | LUAD | -295.7126473 | 1249.919526 | 954.2068787 | 0.735051492 |
| TCGA.50.5931.01A | LUAD | -498.9409518 | -431.4041119 | -930.3450637 | 0.892281228 |

Supplementary Table 11 ESTIMATE

| | | | | | |
|---|---|---|---|---|---|
| TCGA.97.A4LX.01A | LUAD | 1181.290386 | 2646.597101 | 3827.887487 | 0.393025574 |
| TCGA.44.2668.11A | LUAD | 1032.673772 | 2566.170349 | 3598.844121 | 0.423712992 |
| TCGA.50.6597.01A | LUAD | -247.3749243 | 1469.651419 | 1222.276494 | 0.707809967 |
| TCGA.MP.A5C7.01A | LUAD | -1038.026595 | -340.0372276 | -1378.063822 | 0.920004869 |
| TCGA.55.6980.11A | LUAD | 823.5589284 | 2240.439264 | 3063.998192 | 0.493448152 |
| TCGA.38.4628.01A | LUAD | 494.8497476 | 954.4676152 | 1449.317363 | 0.683879019 |
| TCGA.55.8096.01A | LUAD | 1020.624673 | 1540.325312 | 2560.949984 | 0.556270758 |
| TCGA.73.4666.01A | LUAD | 98.96101472 | 2129.067942 | 2228.028957 | 0.59620064 |
| TCGA.53.7813.01A | LUAD | -849.4688978 | 720.571432 | -128.8974659 | 0.833122664 |
| TCGA.93.A4JO.01A | LUAD | 469.5382346 | 2307.105634 | 2776.643869 | 0.529685737 |
| TCGA.55.A48X.01A | LUAD | 232.434799 | 1894.041434 | 2126.476233 | 0.608101634 |
| TCGA.91.6836.01A | LUAD | -842.4777086 | 385.1755775 | -457.3021311 | 0.858806684 |
| TCGA.78.7540.01A | LUAD | -708.8435798 | 724.6890987 | 15.84551895 | 0.821184272 |
| TCGA.49.4488.01A | LUAD | -240.8658969 | 1359.00728 | 1118.141383 | 0.718524808 |
| TCGA.55.8090.01A | LUAD | 116.3899724 | 1093.194746 | 1209.584718 | 0.709124771 |
| TCGA.44.7662.01A | LUAD | 1273.39293 | 1459.404707 | 2732.797637 | 0.535133805 |
| TCGA.55.7283.01A | LUAD | -128.9930755 | 1151.960913 | 1022.967837 | 0.728170788 |
| TCGA.44.3917.01A | LUAD | 377.9600067 | 1198.924625 | 1576.884632 | 0.67009796 |
| TCGA.62.8398.01A | LUAD | -95.63680346 | 1078.043782 | 982.406979 | 0.732238611 |
| TCGA.55.8208.01A | LUAD | 1310.11146 | 2801.412917 | 4111.524378 | 0.354411873 |
| TCGA.78.7536.01A | LUAD | -399.1846195 | 970.9411527 | 571.7565332 | 0.771936567 |
| TCGA.69.7979.01A | LUAD | -60.30856347 | 88.39745513 | 28.08889166 | 0.820157359 |
| TCGA.97.8172.01A | LUAD | 938.2442781 | 2076.370574 | 3014.614852 | 0.499740047 |
| TCGA.50.6592.01A | LUAD | 436.704407 | 1803.114865 | 2239.819272 | 0.594810295 |
| TCGA.73.4677.01A | LUAD | -225.0375652 | 952.0219051 | 726.9843399 | 0.757252618 |
| TCGA.55.A491.01A | LUAD | 248.098811 | 1483.591986 | 1731.690797 | 0.653059192 |
| TCGA.MP.A4SY.01A | LUAD | 613.1852094 | 716.9720923 | 1330.157302 | 0.696535339 |
| TCGA.05.4395.01A | LUAD | -430.2422996 | 1135.275742 | 705.0334419 | 0.759353132 |
| TCGA.44.6147.01B | LUAD | 1298.88555 | 1537.807769 | 2836.693319 | 0.522188798 |
| TCGA.44.A47G.01A | LUAD | 937.3934739 | 2499.081943 | 3436.475417 | 0.445179219 |
| TCGA.55.6972.11A | LUAD | 899.31301 | 2387.469395 | 3286.782405 | 0.464745882 |
| TCGA.05.4389.01A | LUAD | -158.6796756 | 1835.449177 | 1676.769501 | 0.659143168 |
| TCGA.44.6774.01A | LUAD | 1958.742635 | 1367.843103 | 3326.585738 | 0.459564623 |
| TCGA.95.7562.01A | LUAD | 70.58260309 | 1023.783193 | 1094.365796 | 0.720947714 |
| TCGA.86.7714.01A | LUAD | 87.03056043 | 1147.266606 | 1234.297166 | 0.706562422 |
| TCGA.99.8025.01A | LUAD | 128.5111859 | 457.5695949 | 586.0807808 | 0.770598219 |
| TCGA.55.6969.11A | LUAD | 878.432067 | 2294.040577 | 3172.472644 | 0.479536936 |
| TCGA.86.8076.01A | LUAD | 293.372392 | 2274.11701 | 2567.489402 | 0.555472815 |
| TCGA.86.8359.01A | LUAD | -142.9568097 | 1566.499883 | 1423.543073 | 0.686634445 |
| TCGA.05.4398.01A | LUAD | 514.06939 | 1838.237149 | 2352.306539 | 0.581456518 |
| TCGA.86.8075.01A | LUAD | 1241.716831 | 1195.719613 | 2437.436443 | 0.571244855 |
| TCGA.44.3396.01A | LUAD | 1272.409933 | 2234.151907 | 3506.56184 | 0.435943634 |
| TCGA.50.5944.01A | LUAD | 802.342182 | 794.9126306 | 1597.254813 | 0.667875494 |
| TCGA.77.A5GA.01A | LUSC | -865.3419119 | 187.2668138 | -678.0750982 | 0.874954908 |
| TCGA.58.8387.01A | LUSC | -467.4762921 | 1703.080397 | 1235.604105 | 0.706426651 |
| TCGA.43.5668.01A | LUSC | 327.2964842 | 2093.929207 | 2421.225691 | 0.573196322 |
| TCGA.77.7142.11A | LUSC | 1217.963621 | 2151.183397 | 3369.147018 | 0.454007007 |
| TCGA.NC.A5HJ.01A | LUSC | 198.9624417 | 2263.581867 | 2462.544309 | 0.568215963 |
| TCGA.70.6722.01A | LUSC | 706.1729339 | 1218.664626 | 1924.83756 | 0.631328675 |
| TCGA.90.7769.01A | LUSC | -1094.32516 | -461.1556241 | -1555.480784 | 0.929898083 |
| TCGA.56.8504.01A | LUSC | -33.33552038 | 812.5487161 | 779.2131957 | 0.752223193 |
| TCGA.22.5482.11A | LUSC | 1623.412991 | 2510.360624 | 4133.773615 | 0.351356052 |
| TCGA.34.7107.11A | LUSC | 2037.807602 | 2872.53068 | 4910.338282 | 0.242583901 |
| TCGA.66.2800.01A | LUSC | 186.1995418 | 1284.265158 | 1470.4647 | 0.681610929 |
| TCGA.85.7697.01A | LUSC | -169.7309657 | 764.5210116 | 594.7900459 | 0.769782825 |
| TCGA.85.8049.01A | LUSC | 1097.78708 | 2127.543385 | 3225.330464 | 0.472713954 |
| TCGA.18.4086.01A | LUSC | 325.1207233 | 1009.203143 | 1334.323866 | 0.69609637 |
| TCGA.33.4532.01A | LUSC | -1413.675567 | -420.5696926 | -1834.24526 | 0.944166342 |
| TCGA.63.A5MJ.01A | LUSC | 306.9147225 | 351.8444848 | 658.7592073 | 0.763755329 |
| TCGA.NK.A5CX.01A | LUSC | 92.11271624 | 1049.199048 | 1141.311765 | 0.716155157 |
| TCGA.98.8020.01A | LUSC | 401.2437004 | 1043.980455 | 1445.224156 | 0.684317262 |
| TCGA.56.A5DS.01A | LUSC | -395.4859149 | 1130.11101 | 734.6250951 | 0.756519617 |
| TCGA.6A.AB49.01A | LUSC | -2015.583968 | -381.1851765 | -2396.769145 | 0.968123461 |
| TCGA.77.A5G6.01A | LUSC | -1144.667853 | -217.7245079 | -1362.392361 | 0.919100897 |
| TCGA.22.1016.01A | LUSC | 826.3756964 | 1524.093829 | 2350.469526 | 0.58167588 |
| TCGA.66.2785.01A | LUSC | 141.4046311 | 2090.585141 | 2231.989772 | 0.595733769 |
| TCGA.56.A5DR.01A | LUSC | -174.7359098 | 432.6344917 | 257.8985819 | 0.800394259 |
| TCGA.85.7698.01A | LUSC | 482.5444686 | 1022.347851 | 1504.89232 | 0.677904466 |

Supplementary Table 11 ESTIMATE

| | | | | | |
|---|---|---|---|---|---|
| TCGA.39.5022.01A | LUSC | 966.4620875 | 2455.340189 | 3421.802277 | 0.447106829 |
| TCGA.L3.A4E7.01A | LUSC | -358.3692922 | 897.4660813 | 539.0967892 | 0.774975274 |
| TCGA.43.6143.11A | LUSC | 1533.135951 | 2799.511984 | 4332.647935 | 0.323879043 |
| TCGA.63.A5MU.01A | LUSC | -1524.718613 | -514.1045861 | -2038.823199 | 0.95363302 |
| TCGA.77.7338.11A | LUSC | 1146.679158 | 2208.94142 | 3355.620578 | 0.45577521 |
| TCGA.56.A4ZJ.01A | LUSC | 1128.916671 | 1664.488101 | 2793.404772 | 0.527597319 |
| TCGA.21.5786.01A | LUSC | -198.0840888 | 293.9489754 | 95.86488662 | 0.814424818 |
| TCGA.22.1012.01A | LUSC | -385.8655462 | 695.6283419 | 309.7627958 | 0.795807274 |
| TCGA.63.A5MR.01A | LUSC | -893.667759 | 283.2896239 | -610.3781351 | 0.870100187 |
| TCGA.22.5471.01A | LUSC | -110.1677978 | 1525.673429 | 1415.505631 | 0.687491687 |
| TCGA.NC.A5HL.01A | LUSC | -669.1378305 | 1368.839322 | 699.7014917 | 0.759862165 |
| TCGA.18.3411.01A | LUSC | 75.68064789 | 1072.364569 | 1148.045217 | 0.715464968 |
| TCGA.92.8065.01A | LUSC | -37.59032566 | 1444.653472 | 1407.063147 | 0.688391099 |
| TCGA.77.7138.11A | LUSC | 987.5861172 | 2314.412931 | 3301.999048 | 0.462766973 |
| TCGA.NK.A7XE.01A | LUSC | -1406.392493 | 144.8311071 | -1261.561386 | 0.913168589 |
| TCGA.77.8133.01A | LUSC | -308.8182994 | 1602.951234 | 1294.132934 | 0.700319813 |
| TCGA.58.A46J.01A | LUSC | -351.7236537 | 920.5509284 | 568.8272748 | 0.772209835 |
| TCGA.22.5471.11A | LUSC | 857.8590953 | 2121.061271 | 2978.920366 | 0.504271525 |
| TCGA.22.5489.01A | LUSC | 1160.639714 | 1990.260254 | 3150.899968 | 0.482313304 |
| TCGA.66.2758.01A | LUSC | -176.1618968 | 1675.919949 | 1499.758053 | 0.678458319 |
| TCGA.98.A53B.01A | LUSC | -380.9366147 | 816.4991637 | 435.562549 | 0.78449018 |
| TCGA.90.A4ED.01A | LUSC | -84.3135176 | 366.6490526 | 282.335535 | 0.798238765 |
| TCGA.56.7580.01A | LUSC | -562.75713 | 313.8233971 | -248.9337328 | 0.842738214 |
| TCGA.18.3417.01A | LUSC | 911.3794315 | 812.251124 | 1723.630555 | 0.653954742 |
| TCGA.60.2720.01A | LUSC | 66.29078709 | 1509.135763 | 1575.42655 | 0.670256813 |
| TCGA.63.A5MP.01A | LUSC | -180.3950744 | 1522.608791 | 1342.213717 | 0.695264419 |
| TCGA.18.3414.01A | LUSC | -187.1886742 | 842.5116931 | 655.3230189 | 0.764080821 |
| TCGA.22.5473.01A | LUSC | -296.6637052 | 349.9448858 | 53.28118051 | 0.818036027 |
| TCGA.96.7545.01A | LUSC | 522.2181532 | 1485.759349 | 2007.977502 | 0.621817523 |
| TCGA.18.3416.01A | LUSC | -392.8808484 | 2056.165547 | 1663.284699 | 0.660630428 |
| TCGA.NK.A5CT.01A | LUSC | -1248.965464 | 395.6977274 | -853.2677363 | 0.887116157 |
| TCGA.77.8130.01A | LUSC | 404.6132203 | 86.45807908 | 491.0712994 | 0.779411258 |
| TCGA.46.6025.01A | LUSC | -568.4956612 | 15.61060651 | -552.8850547 | 0.865909724 |
| TCGA.66.2789.01A | LUSC | 579.3799762 | 738.6488993 | 1318.028875 | 0.697811649 |
| TCGA.56.8305.01A | LUSC | -288.9224135 | 22.128582 | -266.7938315 | 0.844146615 |
| TCGA.37.A5EL.01A | LUSC | -1638.649302 | -321.5635437 | -1960.212846 | 0.950096669 |
| TCGA.66.2766.01A | LUSC | -382.2190152 | 327.4835532 | -54.73546202 | 0.827052432 |
| TCGA.66.2727.01A | LUSC | -253.1828874 | 781.5177863 | 528.3348988 | 0.775972677 |
| TCGA.63.7022.01A | LUSC | 1646.772645 | 2231.425281 | 3878.197927 | 0.386224218 |
| TCGA.85.8071.01A | LUSC | -1082.164286 | 1.544891331 | -1080.619395 | 0.902022332 |
| TCGA.77.A5G1.01A | LUSC | -10.15312604 | 1194.239608 | 1184.086481 | 0.711758814 |
| TCGA.56.7823.11A | LUSC | 1235.08359 | 2449.165256 | 3684.248846 | 0.412324546 |
| TCGA.63.A5MW.01A | LUSC | 273.9593124 | -287.8488455 | -13.88953313 | 0.82366725 |
| TCGA.66.2794.01A | LUSC | -459.5498808 | 1822.910595 | 1363.360715 | 0.693029964 |
| TCGA.56.8304.01A | LUSC | -1113.612468 | 652.3382618 | -461.2742058 | 0.859105237 |
| TCGA.77.7337.11A | LUSC | 1197.449599 | 2724.789508 | 3922.239108 | 0.380253086 |
| TCGA.33.4587.01A | LUSC | -900.9711931 | -391.5171569 | -1292.48835 | 0.915009481 |
| TCGA.68.7755.01A | LUSC | -144.8311484 | -286.4204459 | -431.2515942 | 0.856841418 |
| TCGA.39.5016.01A | LUSC | -745.435697 | 1391.560147 | 646.1244497 | 0.764951193 |
| TCGA.33.4547.01A | LUSC | 38.5768004 | 704.720329 | 743.2971294 | 0.755686529 |
| TCGA.60.2709.11A | LUSC | 862.7309272 | 2657.388811 | 3520.119738 | 0.434151695 |
| TCGA.60.2719.01A | LUSC | -714.7270158 | 524.4904615 | -190.2365544 | 0.83806876 |
| TCGA.22.5491.01A | LUSC | -1283.89354 | 182.0947756 | -1101.798765 | 0.903360043 |
| TCGA.63.A5MI.01A | LUSC | -1022.316192 | -492.3870716 | -1514.703263 | 0.927679806 |
| TCGA.56.A4BW.01A | LUSC | -92.04048322 | 642.8040146 | 550.7635314 | 0.773891826 |
| TCGA.85.6561.01A | LUSC | 1410.892201 | 1852.024115 | 3262.916317 | 0.467844969 |
| TCGA.90.6837.11A | LUSC | 1526.811861 | 1999.172187 | 3525.984048 | 0.43337608 |
| TCGA.60.2703.01A | LUSC | 303.2354871 | 51.18550116 | 354.4209883 | 0.791820639 |
| TCGA.66.2753.01A | LUSC | 69.67481606 | 713.3252579 | 783.0000739 | 0.75185681 |
| TCGA.18.3407.01A | LUSC | 353.2735745 | 655.1227136 | 1008.396288 | 0.729635138 |
| TCGA.66.2744.01A | LUSC | 673.3776405 | 2468.656494 | 3142.034135 | 0.483452921 |
| TCGA.68.8250.01A | LUSC | -210.33216 | 1020.417127 | 810.0849668 | 0.749229559 |
| TCGA.22.1005.01A | LUSC | 866.706397 | 2749.39464 | 3616.101037 | 0.421417148 |
| TCGA.43.8115.01A | LUSC | 1395.408805 | 1973.29931 | 3368.708115 | 0.45406441 |
| TCGA.85.8288.01A | LUSC | 502.1477128 | 789.6893046 | 1291.837017 | 0.700560343 |
| TCGA.66.2787.01A | LUSC | 180.1917822 | 1357.919422 | 1538.111205 | 0.674311721 |
| TCGA.18.5592.01A | LUSC | -690.0532595 | 99.13497355 | -590.918286 | 0.868688752 |
| TCGA.85.8351.01A | LUSC | -475.5244906 | 362.5879549 | -112.9365357 | 0.83182456 |

Supplementary Table 11 ESTIMATE

| | | | | | |
|---|---|---|---|---|---|
| TCGA.58.8386.11A | LUSC | 1187.11817 | 2969.944786 | 4157.062956 | 0.348153364 |
| TCGA.22.4594.01A | LUSC | 594.3166982 | 1768.951324 | 2363.268022 | 0.580146705 |
| TCGA.22.5483.01A | LUSC | 369.0934455 | 665.6425509 | 1034.735996 | 0.72698573 |
| TCGA.22.4609.11A | LUSC | 1011.30995 | 2359.648029 | 3370.957979 | 0.453770139 |
| TCGA.33.4582.01A | LUSC | -1115.350883 | -283.6871286 | -1399.038011 | 0.921207097 |
| TCGA.18.3410.01A | LUSC | -224.4480162 | 1780.311116 | 1555.8631 | 0.67238521 |
| TCGA.NC.A5HO.01A | LUSC | -947.4995869 | 461.536953 | -485.9626339 | 0.860954338 |
| TCGA.66.2770.01A | LUSC | 430.4901349 | 1167.05881 | 1597.548945 | 0.667843359 |
| TCGA.90.6837.01A | LUSC | 920.1086221 | 1591.1174 | 2511.226022 | 0.562321282 |
| TCGA.94.7033.01A | LUSC | -1525.470699 | 29.81537148 | -1495.655327 | 0.926632214 |
| TCGA.90.7964.01A | LUSC | 33.63190603 | 800.4080316 | 834.0399376 | 0.746896041 |
| TCGA.43.2581.01A | LUSC | 822.716296 | 2078.059535 | 2900.775831 | 0.51414361 |
| TCGA.63.A5MB.01A | LUSC | -798.909494 | 270.1161385 | -528.7933555 | 0.864135414 |
| TCGA.77.8154.01A | LUSC | -931.7803323 | -650.9432173 | -1582.72355 | 0.931361516 |
| TCGA.60.2696.01A | LUSC | 34.2088571 | 1305.318056 | 1339.526913 | 0.695547836 |
| TCGA.98.8023.01A | LUSC | 744.9591394 | 1735.704437 | 2480.663576 | 0.566025344 |
| TCGA.NC.A5HP.01A | LUSC | 148.8003728 | -250.5706851 | -101.7703123 | 0.830913694 |
| TCGA.60.2714.01A | LUSC | -60.32384587 | 1281.085318 | 1220.761472 | 0.707967045 |
| TCGA.43.6770.01A | LUSC | 101.9836196 | 407.2695226 | 509.2531422 | 0.777736395 |
| TCGA.22.5489.11A | LUSC | 914.0708616 | 2280.734358 | 3194.80522 | 0.476657706 |
| TCGA.NC.A5HD.01A | LUSC | -1255.872216 | -694.518137 | -1950.390353 | 0.949645895 |
| TCGA.52.7809.01A | LUSC | -778.356064 | -41.59558346 | -819.9516474 | 0.884848405 |
| TCGA.21.5782.01A | LUSC | 108.771919 | 627.4209664 | 736.1928854 | 0.756369096 |
| TCGA.34.8454.11A | LUSC | 1725.432822 | 2640.309243 | 4365.742065 | 0.31927925 |
| TCGA.85.A513.01A | LUSC | 844.595818 | 2437.060887 | 3281.656705 | 0.465411953 |
| TCGA.NC.A5HF.01A | LUSC | -1462.392707 | -705.7592181 | -2168.151925 | 0.959174479 |
| TCGA.63.A5ML.01A | LUSC | -325.8029842 | 364.1988538 | 38.39586961 | 0.819290808 |
| TCGA.51.4081.11A | LUSC | 1395.135643 | 2956.19602 | 4351.331663 | 0.321283102 |
| TCGA.58.A46M.01A | LUSC | -651.9382026 | 971.8263022 | 319.8880996 | 0.79490638 |
| TCGA.77.8131.01A | LUSC | 927.4383272 | 2596.85286 | 3524.291188 | 0.433600011 |
| TCGA.58.8386.01A | LUSC | 355.0059902 | 1335.558679 | 1690.564669 | 0.657619004 |
| TCGA.39.5040.01A | LUSC | -758.6448626 | 1.46734208 | -757.1775206 | 0.880518052 |
| TCGA.18.4083.01A | LUSC | 52.95204642 | 476.6689282 | 529.6209746 | 0.775853586 |
| TCGA.XC.AA0X.01A | LUSC | 1.483427051 | 1477.472251 | 1478.955678 | 0.680698408 |
| TCGA.63.A5MV.01A | LUSC | 373.9280725 | 1280.527156 | 1654.455229 | 0.661602843 |
| TCGA.56.7822.01A | LUSC | -422.9380054 | 410.7807048 | -12.15730054 | 0.823523032 |
| TCGA.56.8626.01A | LUSC | -209.2881985 | 444.479315 | 235.1911166 | 0.802387971 |
| TCGA.56.7582.01A | LUSC | -234.1797943 | 520.9989417 | 286.8191474 | 0.797842166 |
| TCGA.90.7767.01A | LUSC | 686.4158939 | 1424.2337 | 2110.649594 | 0.609944259 |
| TCGA.90.A59Q.01A | LUSC | 412.7318267 | 2347.155868 | 2759.887695 | 0.531770361 |
| TCGA.85.8052.01A | LUSC | -528.3937986 | 308.8473013 | -219.5464973 | 0.840408211 |
| TCGA.22.4601.01A | LUSC | -720.4193195 | 61.38101533 | -659.0383042 | 0.873598443 |
| TCGA.66.2777.01A | LUSC | 153.6820117 | 1396.548457 | 1550.230469 | 0.672996982 |
| TCGA.22.4593.01A | LUSC | 485.3352834 | 10.16474383 | 495.5000272 | 0.779003807 |
| TCGA.18.3412.01A | LUSC | -407.5423312 | 525.9581152 | 118.415784 | 0.812499547 |
| TCGA.77.6845.01A | LUSC | -988.1738868 | -746.2679816 | -1734.441868 | 0.93923848 |
| TCGA.22.4593.11A | LUSC | 1484.683805 | 2681.25804 | 4165.941846 | 0.346931289 |
| TCGA.22.5480.01A | LUSC | 9.243754863 | 1659.269669 | 1668.513424 | 0.660054051 |
| TCGA.22.5481.11A | LUSC | 738.0722593 | 2418.110824 | 3156.183084 | 0.481633822 |
| TCGA.77.8009.01A | LUSC | -388.5671504 | 13.82863395 | -374.7385165 | 0.85253501 |
| TCGA.22.5474.01A | LUSC | 131.4990923 | 1937.516448 | 2069.015541 | 0.614775779 |
| TCGA.58.A46N.01A | LUSC | -1382.652116 | -443.0219535 | -1825.67407 | 0.943751073 |
| TCGA.68.7757.01B | LUSC | 440.7741447 | 709.5534614 | 1150.327606 | 0.715230861 |
| TCGA.22.4595.01A | LUSC | 470.6261915 | 1193.159408 | 1663.785599 | 0.660575229 |
| TCGA.77.8136.01A | LUSC | 785.130244 | 1915.269248 | 2700.399492 | 0.539145175 |
| TCGA.66.2734.01A | LUSC | 479.36192 | 1418.963379 | 1898.325299 | 0.634341954 |
| TCGA.21.5783.01A | LUSC | -97.65065791 | -321.2263886 | -418.8770465 | 0.855903485 |
| TCGA.21.1082.01A | LUSC | 101.9311759 | 1178.192441 | 1280.123617 | 0.701786253 |
| TCGA.60.2698.01A | LUSC | 205.7300862 | 1772.547911 | 1978.277997 | 0.625225832 |
| TCGA.34.5231.01A | LUSC | -560.937641 | 549.1532894 | -11.78435159 | 0.823491976 |
| TCGA.33.4587.11A | LUSC | 1139.456648 | 2510.225645 | 3649.682293 | 0.416941792 |
| TCGA.66.2757.01A | LUSC | -152.2214011 | 896.8268293 | 744.6054282 | 0.755560739 |
| TCGA.85.A4JB.01A | LUSC | -382.8608174 | 159.6315834 | -223.2292341 | 0.840701059 |
| TCGA.O2.A52N.01A | LUSC | -533.2958869 | 677.1869026 | 143.8910157 | 0.810313904 |
| TCGA.NC.A5HG.01A | LUSC | -1489.674913 | -421.0937109 | -1910.768624 | 0.947807542 |
| TCGA.85.6175.01A | LUSC | 1723.279766 | 1788.352506 | 3511.632273 | 0.43527368 |
| TCGA.85.8287.01A | LUSC | 1113.500361 | 1865.27438 | 2978.774741 | 0.504289984 |
| TCGA.52.7811.01A | LUSC | 97.15792868 | 781.3182331 | 878.4761618 | 0.742542959 |

Supplementary Table 11 ESTIMATE

| | | | | | |
|---|---|---|---|---|---|
| TCGA.37.4129.01A | LUSC | -1110.726467 | -1079.264019 | -2189.990486 | 0.960076154 |
| TCGA.39.5019.01A | LUSC | 142.9364095 | 947.6067006 | 1090.54311 | 0.721336454 |
| TCGA.NC.A5HT.01A | LUSC | -1173.105632 | -1065.20004 | -2238.305672 | 0.962035931 |
| TCGA.77.8140.01A | LUSC | -1194.770345 | -123.1594798 | -1317.929824 | 0.916509721 |
| TCGA.85.7844.01A | LUSC | 523.6045822 | 717.9698066 | 1241.574389 | 0.705806098 |
| TCGA.85.8072.01A | LUSC | -623.1427399 | 717.8656079 | 94.722868 | 0.81452208 |
| TCGA.85.6560.01A | LUSC | 662.1080103 | 1222.095826 | 1884.203836 | 0.635943025 |
| TCGA.60.2709.01A | LUSC | 1343.620035 | 2243.961854 | 3587.58189 | 0.425209843 |
| TCGA.43.7658.11A | LUSC | 605.8303161 | 2269.471781 | 2875.302097 | 0.517347177 |
| TCGA.43.2576.01A | LUSC | 950.3646163 | 1787.68287 | 2738.047486 | 0.534482655 |
| TCGA.22.4613.01A | LUSC | -226.3241754 | 1437.486504 | 1211.162329 | 0.708961472 |
| TCGA.56.A62T.01A | LUSC | -210.6688826 | 434.1667005 | 223.4978179 | 0.803411165 |
| TCGA.22.5482.01A | LUSC | -299.8977701 | -116.1429732 | -416.0407433 | 0.855688108 |
| TCGA.O2.A52Q.01A | LUSC | 265.6777774 | 2106.952722 | 2372.630499 | 0.579026771 |
| TCGA.66.2737.01A | LUSC | 1213.208795 | 1210.084248 | 2423.293042 | 0.572947633 |
| TCGA.77.8144.01A | LUSC | -956.7427306 | 243.932027 | -712.8107035 | 0.877412378 |
| TCGA.39.5024.01A | LUSC | -910.9092029 | 650.4863268 | -260.4228761 | 0.843644883 |
| TCGA.60.2716.01A | LUSC | -1461.521114 | -781.4757465 | -2242.99686 | 0.962223641 |
| TCGA.43.7657.01A | LUSC | -141.5387435 | -398.8070912 | -540.3458347 | 0.864987583 |
| TCGA.NC.A5HH.01A | LUSC | -1143.475838 | -130.6462238 | -1274.122062 | 0.913918521 |
| TCGA.22.0940.01A | LUSC | 424.7874897 | 1993.723392 | 2418.510882 | 0.573522817 |
| TCGA.85.8352.01A | LUSC | -1833.348465 | -729.5995126 | -2562.947978 | 0.973944672 |
| TCGA.85.7696.01A | LUSC | -307.5537008 | 329.2925922 | 21.7388914 | 0.820690296 |
| TCGA.56.6546.01A | LUSC | 346.3954132 | 1874.745239 | 2221.140652 | 0.597012101 |
| TCGA.98.A53I.01A | LUSC | -105.0612706 | 1522.883682 | 1417.822411 | 0.687244687 |
| TCGA.37.4135.01A | LUSC | -441.0832373 | 817.7144636 | 376.6312263 | 0.789825252 |
| TCGA.56.A49D.01A | LUSC | -293.4541421 | 1518.441367 | 1224.987224 | 0.707528831 |
| TCGA.85.7843.01A | LUSC | 263.423722 | 687.8309056 | 951.2546276 | 0.735345244 |
| TCGA.85.A4JC.01A | LUSC | 238.9625227 | 1074.019278 | 1312.981801 | 0.698342115 |
| TCGA.NC.A5HE.01A | LUSC | 328.5198308 | 2666.702747 | 2995.222577 | 0.502203641 |
| TCGA.34.5234.01A | LUSC | 1457.352165 | 2290.550067 | 3747.902232 | 0.403794325 |
| TCGA.52.7812.01A | LUSC | -350.5048425 | 1539.730369 | 1189.225526 | 0.711228737 |
| TCGA.21.1076.01A | LUSC | 1193.685166 | 2482.577996 | 3676.263161 | 0.413392185 |
| TCGA.39.5036.01A | LUSC | -1592.987823 | 216.981013 | -1376.00681 | 0.919886492 |
| TCGA.58.A46K.01A | LUSC | -878.3756577 | 135.637983 | -742.7376747 | 0.879511357 |
| TCGA.22.4609.01A | LUSC | 783.6200041 | 1926.026291 | 2709.646295 | 0.538001523 |
| TCGA.77.A5GB.01B | LUSC | 301.6208888 | 1238.443092 | 1540.063981 | 0.674100021 |
| TCGA.O2.A52S.01A | LUSC | -923.1881735 | 789.9066803 | -133.2814932 | 0.833478417 |
| TCGA.85.8277.01A | LUSC | -695.5375285 | 483.00393 | -212.5335985 | 0.839849872 |
| TCGA.37.3792.01A | LUSC | 671.8184996 | 2673.106132 | 3344.924632 | 0.457172133 |
| TCGA.96.A4JL.01A | LUSC | 409.7844213 | 2369.144043 | 2778.928464 | 0.529401264 |
| TCGA.43.7658.01A | LUSC | -1272.337707 | 982.1969681 | -290.1407389 | 0.845978941 |
| TCGA.56.7731.01A | LUSC | 693.2334909 | 1489.537849 | 2182.77134 | 0.601520916 |
| TCGA.51.4081.01A | LUSC | -151.7702157 | -616.7511862 | -768.521402 | 0.881306133 |
| TCGA.22.5472.11A | LUSC | 1501.52192 | 2318.661441 | 3820.183361 | 0.394065195 |
| TCGA.34.2600.01A | LUSC | -1355.512286 | -141.4996993 | -1497.011985 | 0.926707066 |
| TCGA.58.8390.01A | LUSC | 270.540835 | 507.9160076 | 778.4568425 | 0.752296343 |
| TCGA.92.8064.01A | LUSC | 733.5056156 | 2141.549355 | 2875.054971 | 0.51737822 |
| TCGA.37.A5EN.01A | LUSC | -887.7851499 | 300.3785873 | -587.4065626 | 0.868433289 |
| TCGA.34.5232.01A | LUSC | -468.0981097 | 2007.934155 | 1539.836045 | 0.674124735 |
| TCGA.85.7950.01A | LUSC | 589.0548585 | 1424.267688 | 2013.322547 | 0.62120287 |
| TCGA.63.A5MM.01A | LUSC | 791.0404941 | 1928.910972 | 2719.951466 | 0.536725804 |
| TCGA.56.8623.01A | LUSC | 1181.415219 | 2813.606855 | 3995.022074 | 0.37035041 |
| TCGA.18.4721.01A | LUSC | 675.4800739 | 1737.980019 | 2413.460093 | 0.574130004 |
| TCGA.34.5927.01A | LUSC | -842.526734 | -401.3107296 | -1243.837464 | 0.912105108 |
| TCGA.77.A5G8.01B | LUSC | -335.1811394 | 565.4979448 | 230.3168053 | 0.802814774 |
| TCGA.92.8063.01A | LUSC | -80.33617524 | 443.3058717 | 362.9696964 | 0.791053611 |
| TCGA.18.3419.01A | LUSC | -797.7586634 | 1192.577461 | 394.818798 | 0.788185012 |
| TCGA.39.5011.01A | LUSC | 34.17668607 | 2259.640897 | 2293.817583 | 0.588419973 |
| TCGA.33.A4WN.01A | LUSC | -948.0345649 | 177.3596779 | -770.674887 | 0.881455464 |
| TCGA.51.4080.01A | LUSC | -1066.592589 | -243.8711238 | -1310.463713 | 0.916070781 |
| TCGA.56.8629.01A | LUSC | -555.6862964 | 143.450048 | -412.2362484 | 0.855398979 |
| TCGA.NC.A5HR.01A | LUSC | -1336.603163 | -107.2969971 | -1443.90016 | 0.923749242 |
| TCGA.56.8622.01A | LUSC | 226.9019346 | 535.3096273 | 762.2115619 | 0.753865249 |
| TCGA.21.A5DI.01A | LUSC | -775.2503207 | -77.22569943 | -852.4760202 | 0.887062513 |
| TCGA.60.2708.01A | LUSC | 811.6118108 | 203.1477886 | 1014.759599 | 0.728996075 |
| TCGA.33.4566.01A | LUSC | -88.65753507 | 1564.480049 | 1475.822514 | 0.681035251 |
| TCGA.34.5929.01A | LUSC | -395.1230308 | 536.9579547 | 141.8349239 | 0.810490727 |

Supplementary Table 11 ESTIMATE

| | | | | | |
|---|---|---|---|---|---|
| TCGA.56.6545.01A | LUSC | 1388.224627 | 1734.681191 | 3122.905818 | 0.485908891 |
| TCGA.85.A53L.01A | LUSC | -690.3749708 | 127.3949679 | -562.9800028 | 0.86664998 |
| TCGA.85.8584.01A | LUSC | -136.0411441 | 1677.655856 | 1541.614712 | 0.673931868 |
| TCGA.85.8354.01A | LUSC | -1322.443295 | -175.8869173 | -1498.330212 | 0.926779763 |
| TCGA.18.5595.01A | LUSC | -1109.871182 | -650.0121051 | -1759.883287 | 0.940513854 |
| TCGA.LA.A446.01A | LUSC | -1227.448612 | 63.4806755 | -1163.967936 | 0.907236237 |
| TCGA.66.2782.01A | LUSC | 424.8984045 | 1622.23762 | 2047.136025 | 0.617305646 |
| TCGA.60.2695.01A | LUSC | -290.203116 | 736.2805186 | 446.0774026 | 0.783532083 |
| TCGA.34.2608.01A | LUSC | 1265.888971 | 1814.915687 | 3080.804658 | 0.491300923 |
| TCGA.22.0944.01A | LUSC | -970.559237 | -213.200665 | -1183.759902 | 0.90845441 |
| TCGA.77.A5GF.01A | LUSC | -333.5068794 | 411.1894249 | 77.68254551 | 0.815970629 |
| TCGA.51.4079.01A | LUSC | -64.51713159 | 745.6012765 | 681.0841449 | 0.761635883 |
| TCGA.37.4141.01A | LUSC | -172.7767982 | 887.8319796 | 715.0551814 | 0.758395113 |
| TCGA.33.4538.01A | LUSC | -832.9755603 | 218.6485562 | -614.3270041 | 0.870385735 |
| TCGA.85.7699.01A | LUSC | 615.4779658 | 1912.286324 | 2527.76429 | 0.560312177 |
| TCGA.58.8392.01A | LUSC | -1348.253279 | -385.842818 | -1734.096097 | 0.939221057 |
| TCGA.21.1079.01A | LUSC | 401.9978953 | 369.9838071 | 771.9817024 | 0.7529222 |
| TCGA.85.8048.01A | LUSC | 868.0607746 | 2234.556192 | 3102.616966 | 0.488509679 |
| TCGA.43.6647.11A | LUSC | 1190.880454 | 2445.84468 | 3636.725135 | 0.418669792 |
| TCGA.56.A4ZK.01A | LUSC | -246.9833668 | 1329.038412 | 1082.055046 | 0.72219882 |
| TCGA.56.7222.11A | LUSC | 192.0009358 | 1905.623076 | 2097.624011 | 0.6114583 |
| TCGA.56.5898.01A | LUSC | 68.54726363 | 767.4869648 | 836.0342284 | 0.746701356 |
| TCGA.37.4133.01A | LUSC | -233.9211283 | 1035.467978 | 801.5468497 | 0.750059041 |
| TCGA.94.7943.01A | LUSC | -685.2269624 | 418.5203769 | -266.7065855 | 0.844139749 |
| TCGA.34.A5IX.01A | LUSC | -935.7337077 | 505.0268008 | -430.7069069 | 0.856800193 |
| TCGA.98.A538.01A | LUSC | -259.0901289 | 549.7606018 | 290.6704729 | 0.79750122 |
| TCGA.43.8116.01A | LUSC | -513.9728636 | 873.1038527 | 359.1309891 | 0.791398191 |
| TCGA.85.7710.11A | LUSC | 939.9173902 | 3295.323065 | 4235.240455 | 0.337373065 |
| TCGA.77.6842.01A | LUSC | 345.34016 | 2158.013927 | 2503.354087 | 0.563276418 |
| TCGA.68.8251.01A | LUSC | 598.7078698 | 1104.527601 | 1703.23547 | 0.65621669 |
| TCGA.LA.A7SW.01A | LUSC | -653.3844808 | 228.2510306 | -425.1334502 | 0.856378044 |
| TCGA.63.A5MS.01A | LUSC | 614.5672832 | 1613.97015 | 2228.537433 | 0.596140716 |
| TCGA.66.2786.01A | LUSC | -26.90255223 | 1269.835913 | 1242.933361 | 0.70566477 |
| TCGA.21.1072.01A | LUSC | -431.8332895 | -77.32771009 | -509.1609996 | 0.862681533 |
| TCGA.56.5897.01A | LUSC | 1398.747628 | 1792.24023 | 3190.987858 | 0.477150223 |
| TCGA.52.7622.01A | LUSC | 288.0182248 | 1718.135727 | 2006.153952 | 0.622027134 |
| TCGA.43.2578.01A | LUSC | -437.1182477 | 562.3971275 | 125.2788798 | 0.811911846 |
| TCGA.NC.A5HQ.01A | LUSC | -959.7488396 | 1031.063865 | 71.31502577 | 0.816510604 |
| TCGA.66.2793.01A | LUSC | -1660.237187 | -885.3571156 | -2545.594303 | 0.973363817 |
| TCGA.56.8624.01A | LUSC | 106.8456061 | 529.3525492 | 636.1981554 | 0.765888859 |
| TCGA.58.A46L.01A | LUSC | -63.42284548 | 880.3990794 | 816.9762339 | 0.74855921 |
| TCGA.56.8309.11A | LUSC | 1017.530243 | 2501.223845 | 3518.754088 | 0.43433227 |
| TCGA.63.A5MT.01A | LUSC | 287.7187796 | 1132.054141 | 1419.77292 | 0.687036674 |
| TCGA.39.5035.01A | LUSC | -33.93219463 | 1704.928883 | 1670.996689 | 0.659780177 |
| TCGA.52.7810.01A | LUSC | -672.4432121 | -252.3171095 | -924.7603216 | 0.891910816 |
| TCGA.56.7580.11A | LUSC | 1413.111835 | 2282.921107 | 3696.032942 | 0.410748047 |
| TCGA.18.3415.01A | LUSC | 305.9675772 | 1266.670431 | 1572.638008 | 0.670560529 |
| TCGA.43.6647.01A | LUSC | 1391.941814 | 1610.614761 | 3002.556576 | 0.501272407 |
| TCGA.94.A5I6.01A | LUSC | -507.5200743 | -61.52735001 | -569.0474243 | 0.867093985 |
| TCGA.33.6737.01A | LUSC | 80.36492792 | 930.9632122 | 1011.32814 | 0.729340773 |
| TCGA.NC.A5HN.01A | LUSC | -1369.218068 | -324.9150855 | -1694.133153 | 0.937191001 |
| TCGA.94.8490.01A | LUSC | -1316.007627 | -576.9798263 | -1892.987454 | 0.946972112 |
| TCGA.77.7335.11A | LUSC | 1388.230573 | 2710.117169 | 4098.347742 | 0.356219838 |
| TCGA.92.7340.01A | LUSC | 1249.571299 | 1284.786914 | 2534.358213 | 0.559510212 |
| TCGA.21.1077.01A | LUSC | -36.42246662 | 773.8252638 | 737.4027971 | 0.756252907 |
| TCGA.21.1081.01A | LUSC | -37.43071936 | -167.1860948 | -204.6168142 | 0.8392185 |
| TCGA.21.1075.01A | LUSC | -1213.280684 | -752.3417264 | -1965.62241 | 0.950344081 |
| TCGA.85.A5B5.01A | LUSC | -685.1595413 | -179.5260163 | -864.6855576 | 0.887888464 |
| TCGA.39.5028.01A | LUSC | 458.2693966 | 1186.702842 | 1644.972239 | 0.662645993 |
| TCGA.85.A511.01A | LUSC | 679.5716443 | 1119.513348 | 1799.084993 | 0.64553556 |
| TCGA.34.7107.01A | LUSC | 603.6495563 | 426.5656607 | 1030.215217 | 0.727441231 |
| TCGA.85.A50Z.01A | LUSC | -803.9533042 | 1020.007091 | 216.0537868 | 0.804061305 |
| TCGA.85.A4CN.01A | LUSC | 278.0450206 | 447.9116583 | 725.9566788 | 0.757351132 |
| TCGA.33.AASB.01A | LUSC | -1950.351208 | -714.722859 | -2665.074067 | 0.977234838 |
| TCGA.60.2704.01A | LUSC | 1558.717165 | 2054.205533 | 3612.922698 | 0.421840195 |
| TCGA.21.1071.01A | LUSC | 90.79672488 | 735.6844683 | 826.4811931 | 0.747633357 |
| TCGA.77.8156.01A | LUSC | 547.6002871 | 1943.76867 | 2491.368957 | 0.564729182 |
| TCGA.66.2756.01A | LUSC | -511.0521988 | 150.4862147 | -360.5659841 | 0.851445822 |

Supplementary Table 11 ESTIMATE

| | | | | | |
|---|---|---|---|---|---|
| TCGA.63.A5MN.01A | LUSC | 1088.06522 | 2595.464404 | 3683.529624 | 0.412420725 |
| TCGA.63.A5M9.01A | LUSC | -326.0088722 | 529.8471908 | 203.8383185 | 0.805126086 |
| TCGA.34.8455.01A | LUSC | -14.42081703 | 376.8590602 | 362.4382432 | 0.791101332 |
| TCGA.66.2791.01A | LUSC | 626.4493734 | 1909.025194 | 2535.474567 | 0.559374387 |
| TCGA.43.A56V.01A | LUSC | -667.6019145 | 249.6576655 | -417.944249 | 0.855832669 |
| TCGA.43.3394.01A | LUSC | -388.8722231 | 88.96483089 | -299.9073922 | 0.846742507 |
| TCGA.77.A5FZ.01A | LUSC | 1581.82408 | 3314.16031 | 4895.98439 | 0.244627411 |
| TCGA.58.8393.01A | LUSC | 175.1356658 | 1986.2659 | 2161.401566 | 0.604023835 |
| TCGA.33.A5GW.01A | LUSC | -1581.830793 | -648.1799378 | -2230.01073 | 0.961702906 |
| TCGA.77.7139.01A | LUSC | -520.3128461 | 164.7756774 | -355.5371687 | 0.851058461 |
| TCGA.56.7222.01A | LUSC | -1161.105794 | -239.0918888 | -1400.197683 | 0.921273314 |
| TCGA.63.7023.01A | LUSC | -678.649552 | 232.5607426 | -446.0888094 | 0.857962283 |
| TCGA.60.2697.01A | LUSC | 1022.362914 | 2691.956017 | 3714.318932 | 0.40829928 |
| TCGA.22.5492.01A | LUSC | 187.0468254 | 1828.017118 | 2015.063943 | 0.621002536 |
| TCGA.37.3783.01A | LUSC | 429.4613098 | 1938.004711 | 2367.466021 | 0.579644678 |
| TCGA.33.4533.01A | LUSC | 0.011594767 | -204.1410416 | -204.1294468 | 0.839179594 |
| TCGA.33.6738.01A | LUSC | -1090.625958 | 317.8001097 | -772.8258484 | 0.881604532 |
| TCGA.77.8008.01A | LUSC | 1590.545598 | 1536.190077 | 3126.735675 | 0.485417464 |
| TCGA.33.AASJ.01A | LUSC | -1683.212443 | -296.3613323 | -1979.573775 | 0.950979396 |
| TCGA.22.1011.01A | LUSC | 222.0305509 | 1802.437291 | 2024.467842 | 0.619919988 |
| TCGA.MF.A522.01A | LUSC | -1371.797102 | -275.2977473 | -1647.094849 | 0.934760214 |
| TCGA.66.2790.01A | LUSC | 223.3126983 | 993.1893982 | 1216.502096 | 0.708408471 |
| TCGA.43.6771.01A | LUSC | 255.5554803 | 825.805647 | 1081.361127 | 0.722269271 |
| TCGA.60.2726.01A | LUSC | -767.7676696 | 917.5747958 | 149.8071262 | 0.80980471 |
| TCGA.85.8580.01A | LUSC | -1166.59905 | 442.9146096 | -723.6844409 | 0.878176987 |
| TCGA.39.5037.01A | LUSC | -2045.23359 | -815.1965545 | -2860.430145 | 0.982916151 |
| TCGA.33.4586.01A | LUSC | -558.6702769 | -75.5520343 | -634.2223112 | 0.87181994 |
| TCGA.66.2795.01A | LUSC | 715.6672027 | 1234.008698 | 1949.675901 | 0.628496971 |
| TCGA.90.7766.01A | LUSC | -813.8694383 | 1352.566741 | 538.6973023 | 0.775012333 |
| TCGA.39.5021.01A | LUSC | 47.0704494 | 1164.158336 | 1211.228785 | 0.708954593 |
| TCGA.98.A53J.01A | LUSC | -1101.048181 | -147.4988274 | -1248.547009 | 0.912388295 |
| TCGA.66.2763.01A | LUSC | -398.1523163 | 667.4284147 | 269.2760984 | 0.799391969 |
| TCGA.33.AASL.01A | LUSC | -1327.52773 | 537.9913644 | -789.536366 | 0.882759625 |
| TCGA.98.A539.01A | LUSC | -267.1972627 | 547.487243 | 280.2899803 | 0.79841959 |
| TCGA.77.6844.01A | LUSC | -776.0950473 | 414.1491479 | -361.9458994 | 0.851552033 |
| TCGA.77.8139.01A | LUSC | -12.09642161 | 416.6434969 | 404.5470753 | 0.787305364 |
| TCGA.98.A53C.01A | LUSC | 1425.41044 | 2812.937974 | 4238.348413 | 0.336943564 |
| TCGA.46.3767.01A | LUSC | 1608.711249 | 1904.451382 | 3513.162631 | 0.435071427 |
| TCGA.60.2710.01A | LUSC | 216.1838506 | 1135.911296 | 1352.095147 | 0.69422115 |
| TCGA.96.A4JK.01A | LUSC | -370.992664 | 721.4343691 | 350.4417051 | 0.792177253 |
| TCGA.66.2771.01A | LUSC | 287.5267457 | 2219.430091 | 2506.956836 | 0.562839374 |
| TCGA.94.8491.01A | LUSC | -623.3070801 | 1189.331895 | 566.0248147 | 0.772471139 |
| TCGA.85.A510.01A | LUSC | -390.8287289 | 1078.241729 | 687.4130005 | 0.761033555 |
| TCGA.22.5478.01A | LUSC | 182.1144317 | 615.8650067 | 797.9794384 | 0.750405268 |
| TCGA.66.2769.01A | LUSC | 916.9142195 | 1908.978132 | 2825.892351 | 0.523540266 |
| TCGA.85.8664.01A | LUSC | -1760.195861 | -410.5723807 | -2170.768242 | 0.959283022 |
| TCGA.56.8623.11A | LUSC | 1155.929643 | 2841.254284 | 3997.183927 | 0.370055622 |
| TCGA.22.4599.01A | LUSC | 201.905118 | 2024.467635 | 2226.372753 | 0.596395801 |
| TCGA.94.A5I4.01A | LUSC | -879.8847383 | 29.00629635 | -850.878442 | 0.886954229 |
| TCGA.21.1083.01A | LUSC | -53.88072768 | 976.6207747 | 922.740047 | 0.738175351 |
| TCGA.34.8454.01A | LUSC | 507.4748122 | 1885.039178 | 2392.51399 | 0.576644695 |
| TCGA.66.2754.01A | LUSC | -1120.065636 | -519.6163134 | -1639.681949 | 0.934373072 |
| TCGA.33.6737.11A | LUSC | 1510.872463 | 2588.804463 | 4099.676926 | 0.356037521 |
| TCGA.NC.A5HK.01A | LUSC | -1338.735044 | 98.09570403 | -1240.63934 | 0.911912554 |
| TCGA.85.8353.01A | LUSC | 830.6644056 | 1283.150734 | 2113.81514 | 0.609575971 |
| TCGA.22.1017.01A | LUSC | -524.2092428 | 1080.931235 | 556.721992 | 0.773337609 |
| TCGA.98.8022.01A | LUSC | 24.4061928 | 1215.973987 | 1240.38018 | 0.705930268 |
| TCGA.60.2721.01A | LUSC | 353.2901275 | 1662.385256 | 2015.675384 | 0.620932184 |
| TCGA.22.A5C4.01A | LUSC | -929.793352 | 109.8497767 | -819.9435754 | 0.884847853 |
| TCGA.46.3765.01A | LUSC | -25.35674217 | 928.5907374 | 903.2339952 | 0.740103905 |
| TCGA.39.5029.01A | LUSC | -85.33207252 | 1148.172164 | 1062.840091 | 0.724146866 |
| TCGA.22.4605.01A | LUSC | 650.4730408 | 1180.336608 | 1830.809649 | 0.641972025 |
| TCGA.96.8170.01A | LUSC | -125.6474852 | 912.9308864 | 787.2834012 | 0.751442114 |
| TCGA.34.5236.01A | LUSC | 906.3751098 | 906.0331578 | 1812.408268 | 0.644040697 |
| TCGA.77.7463.01A | LUSC | -889.325476 | 373.6930051 | -515.6324709 | 0.863161572 |
| TCGA.37.4132.01A | LUSC | -54.39444584 | 1356.585374 | 1302.190928 | 0.699474991 |
| TCGA.66.2778.01A | LUSC | -396.5818452 | 1276.358492 | 879.7766467 | 0.742415084 |
| TCGA.37.5819.01A | LUSC | -459.2335506 | 443.1389128 | -16.09463787 | 0.823850759 |

Supplementary Table 11 ESTIMATE

| | | | | | |
|---|---|---|---|---|---|
| TCGA.43.6773.01A | LUSC | 1246.381663 | 2254.697272 | 3501.078934 | 0.436667816 |
| TCGA.39.5040.11A | LUSC | 893.7816647 | 2457.92449 | 3351.706155 | 0.456286576 |
| TCGA.NC.A5HI.01A | LUSC | -1420.968049 | 108.7357565 | -1312.232292 | 0.916174857 |
| TCGA.56.7582.11A | LUSC | 1354.849178 | 2081.022057 | 3435.871236 | 0.445258631 |
| TCGA.77.A5G3.01A | LUSC | -611.6879253 | 140.8843158 | -470.8036096 | 0.859820303 |
| TCGA.21.1070.01A | LUSC | 96.38719215 | 1972.985351 | 2069.372543 | 0.614734447 |
| TCGA.60.2715.01A | LUSC | 1429.802615 | 2128.468214 | 3558.270829 | 0.429100087 |
| TCGA.60.2712.01A | LUSC | 943.9372782 | 2369.759574 | 3313.696852 | 0.461244115 |
| TCGA.60.2707.01A | LUSC | 556.9332353 | 920.5927636 | 1477.525999 | 0.680852129 |
| TCGA.63.5128.01A | LUSC | -528.5663354 | -366.532848 | -895.0991834 | 0.889933481 |
| TCGA.98.8021.01A | LUSC | -38.16176476 | 245.605046 | 207.4432813 | 0.804812123 |
| TCGA.63.A5MH.01A | LUSC | -721.6907653 | -259.2365498 | -980.9273151 | 0.895608791 |
| TCGA.39.5034.01A | LUSC | 492.2675013 | 720.479193 | 1212.746694 | 0.708797436 |
| TCGA.18.3409.01A | LUSC | 1455.752853 | 1091.920818 | 2547.673671 | 0.557889163 |
| TCGA.60.2723.01A | LUSC | 277.9253322 | 1402.762238 | 1680.68757 | 0.658710553 |
| TCGA.85.A4QQ.01A | LUSC | -1219.844235 | 12.31769199 | -1207.526543 | 0.909907085 |
| TCGA.60.2711.01A | LUSC | -963.2746172 | 442.4562586 | -520.8183586 | 0.863545688 |
| TCGA.22.5485.01A | LUSC | -574.932343 | 219.1276447 | -355.8046983 | 0.85107908 |
| TCGA.66.2788.01A | LUSC | 929.0613814 | 1516.862621 | 2445.924003 | 0.570221822 |
| TCGA.77.8153.01A | LUSC | -1208.588928 | -700.4703213 | -1909.05925 | 0.947727509 |
| TCGA.46.3769.01A | LUSC | 382.3687763 | 1526.607094 | 1908.975871 | 0.633132604 |
| TCGA.22.5491.11A | LUSC | 931.0594233 | 2011.099784 | 2942.159207 | 0.508923947 |
| TCGA.56.7579.01A | LUSC | -328.4868269 | 285.8549152 | -42.63191174 | 0.826052422 |
| TCGA.43.A475.01A | LUSC | 75.76769996 | 2470.867628 | 2546.635328 | 0.55801565 |
| TCGA.22.1000.01A | LUSC | 1571.720113 | 2224.637078 | 3796.35719 | 0.397277182 |
| TCGA.39.5030.01A | LUSC | 1090.467433 | 2575.143108 | 3665.610542 | 0.414815494 |
| TCGA.22.4604.01A | LUSC | 1071.230595 | 1623.019865 | 2694.25046 | 0.539905142 |
| TCGA.56.8628.01A | LUSC | 664.4017694 | 1740.364007 | 2404.765777 | 0.575174463 |
| TCGA.66.2783.01A | LUSC | -62.62924746 | 850.0939172 | 787.4646698 | 0.751424558 |
| TCGA.56.8083.01A | LUSC | -882.3316781 | -1178.449769 | -2060.781448 | 0.954598162 |
| TCGA.77.8143.01A | LUSC | -1254.200897 | 94.92660899 | -1159.274288 | 0.906946225 |
| TCGA.94.A4VJ.01A | LUSC | -287.4381929 | 473.1225601 | 185.6843672 | 0.806703722 |
| TCGA.66.2780.01A | LUSC | 379.9252692 | 874.4072181 | 1254.332487 | 0.704478202 |
| TCGA.21.1080.01A | LUSC | -712.0563649 | 593.2396959 | -118.816669 | 0.832303323 |
| TCGA.56.A4BX.01A | LUSC | -1927.686755 | -1124.824921 | -3052.511676 | 0.987714254 |
| TCGA.77.7337.01A | LUSC | 752.0629567 | 698.2926643 | 1450.355621 | 0.683767818 |
| TCGA.77.8145.01A | LUSC | 572.9028511 | 176.359329 | 749.2621801 | 0.755112779 |
| TCGA.NK.A5D1.01A | LUSC | -1219.735124 | -515.6978254 | -1735.432949 | 0.939288408 |
| TCGA.43.6773.11A | LUSC | 1852.819209 | 2740.833364 | 4593.652573 | 0.287402883 |
| TCGA.34.5241.01A | LUSC | 893.360227 | 1719.999568 | 2613.359795 | 0.549861342 |
| TCGA.56.8082.11A | LUSC | 1129.42073 | 2461.733605 | 3591.154334 | 0.424735159 |
| TCGA.46.3768.01A | LUSC | -595.6119604 | 247.1049472 | -348.5070133 | 0.850516164 |
| TCGA.46.6026.01A | LUSC | -1454.870211 | -423.7357051 | -1878.605916 | 0.946291691 |
| TCGA.56.8503.01A | LUSC | 1380.543766 | 1762.923957 | 3143.467722 | 0.483268703 |
| TCGA.60.2725.01A | LUSC | 807.1552068 | 2134.088394 | 2941.2436 | 0.509039636 |
| TCGA.68.7756.01A | LUSC | -77.43016555 | 384.9711424 | 307.5409769 | 0.796004724 |
| TCGA.60.2706.01A | LUSC | -34.69618481 | 1514.12304 | 1479.426855 | 0.680647739 |
| TCGA.85.8666.01A | LUSC | 511.6128344 | 1079.135383 | 1590.748217 | 0.668586039 |
| TCGA.70.6723.01A | LUSC | -1500.00883 | -600.1270815 | -2100.135912 | 0.956303107 |
| TCGA.56.8082.01A | LUSC | -989.0948431 | -332.1724474 | -1321.26729 | 0.916705577 |
| TCGA.77.8007.01A | LUSC | 785.5777487 | 1968.609324 | 2754.187073 | 0.532478838 |
| TCGA.77.7338.01A | LUSC | 512.8016023 | 712.7012527 | 1225.502855 | 0.707475341 |
| TCGA.85.8481.01A | LUSC | 509.3243464 | 980.8415082 | 1490.165855 | 0.679492035 |
| TCGA.77.A5GH.01A | LUSC | 574.7413274 | 2127.396417 | 2702.137745 | 0.538930262 |
| TCGA.39.5027.01A | LUSC | -1820.980145 | -679.4522239 | -2500.432369 | 0.971822579 |
| TCGA.96.7544.01A | LUSC | 973.204172 | 1142.674804 | 2115.878976 | 0.609335789 |
| TCGA.77.7138.01A | LUSC | -862.6071429 | 128.5720157 | -734.0351272 | 0.878902737 |
| TCGA.O2.A52W.01A | LUSC | -1219.859168 | 164.8769762 | -1054.982192 | 0.900391396 |
| TCGA.66.2792.01A | LUSC | -129.0818792 | 238.6762887 | 109.5944094 | 0.813253729 |
| TCGA.56.A4BY.01A | LUSC | -702.8450477 | 190.6178752 | -512.2271724 | 0.862909072 |
| TCGA.43.5670.11A | LUSC | 1153.566587 | 2339.025298 | 3492.591886 | 0.437788227 |
| TCGA.22.4607.01A | LUSC | 166.2691628 | 849.5184948 | 1015.787658 | 0.728892768 |
| TCGA.37.A5EM.01A | LUSC | 808.9061223 | 1326.615055 | 2135.521178 | 0.607047097 |
| TCGA.63.A5MY.01A | LUSC | -1971.214835 | -868.9181994 | -2840.133034 | 0.982363423 |
| TCGA.85.8355.01A | LUSC | 421.878545 | 2674.081031 | 3095.959576 | 0.489362134 |
| TCGA.56.8309.01A | LUSC | 757.7696069 | 2680.869806 | 3438.639413 | 0.44489476 |
| TCGA.43.7657.11A | LUSC | 1455.429659 | 2339.277624 | 3794.707284 | 0.397499425 |
| TCGA.85.8350.01A | LUSC | 146.2398611 | 772.1605515 | 918.4004126 | 0.738604933 |

Supplementary Table 11 ESTIMATE

| | | | | | |
|---|---|---|---|---|---|
| TCGA.46.3766.01A | LUSC | 1835.469847 | 2408.762219 | 4244.232066 | 0.336130289 |
| TCGA.56.7823.01B | LUSC | -1124.191844 | -136.2380566 | -1260.429901 | 0.913100882 |
| TCGA.21.1078.01A | LUSC | -1637.220124 | -508.3825477 | -2145.602672 | 0.958233118 |
| TCGA.60.2724.01A | LUSC | 528.8692823 | 341.2477234 | 870.1170057 | 0.743364261 |
| TCGA.39.5031.01A | LUSC | 194.7218152 | 1080.486293 | 1275.208108 | 0.702300086 |
| TCGA.22.1002.01A | LUSC | 600.7101473 | 1216.520391 | 1817.230539 | 0.643499034 |
| TCGA.43.6771.11A | LUSC | 1257.65806 | 2527.856826 | 3785.514886 | 0.398737218 |
| TCGA.34.5240.01A | LUSC | 186.5508868 | 469.7163985 | 656.2672854 | 0.763991395 |
| TCGA.43.3920.01A | LUSC | 771.3351803 | 1231.192134 | 2002.527314 | 0.622443873 |
| TCGA.77.8150.01A | LUSC | -420.4888338 | 972.05516 | 551.5663262 | 0.773817189 |
| TCGA.33.AAS8.01A | LUSC | -1628.66324 | -267.6429448 | -1896.306185 | 0.947128529 |
| TCGA.77.6843.01A | LUSC | -1319.401162 | -728.9566911 | -2048.357853 | 0.95405332 |
| TCGA.85.8582.01A | LUSC | 1005.57745 | 865.4403105 | 1871.017761 | 0.637435576 |
| TCGA.56.8307.01A | LUSC | -653.9571666 | 1282.195979 | 628.2388129 | 0.766639547 |
| TCGA.56.8083.11A | LUSC | 1017.388015 | 2152.635277 | 3170.023292 | 0.479852406 |
| TCGA.90.7767.11A | LUSC | 1099.976987 | 2081.306825 | 3181.283813 | 0.478401569 |
| TCGA.58.8388.01A | LUSC | 399.5940401 | 1236.235538 | 1635.829579 | 0.663650492 |
| TCGA.77.7141.01A | LUSC | -763.4771656 | -92.08017731 | -855.5573429 | 0.887271227 |
| TCGA.56.7221.01A | LUSC | -1255.370699 | -397.018367 | -1652.389066 | 0.93503603 |
| TCGA.90.A4EE.01A | LUSC | 729.736373 | 2648.884862 | 3378.621234 | 0.452767453 |
| TCGA.34.2596.01A | LUSC | -187.9967884 | 835.3870346 | 647.3902461 | 0.764831506 |
| TCGA.58.8391.01A | LUSC | -1057.596988 | -167.0428881 | -1224.639876 | 0.910946237 |
| TCGA.22.5472.01A | LUSC | -203.974566 | 906.6980098 | 702.7234438 | 0.759573721 |
| TCGA.85.8070.01A | LUSC | -1848.020835 | -878.2869143 | -2726.307749 | 0.979102319 |
| TCGA.85.A4PA.01A | LUSC | 166.9116758 | 3390.166544 | 3557.07822 | 0.429258206 |
| TCGA.33.4589.01A | LUSC | -188.4212317 | 1526.773131 | 1338.351899 | 0.695671748 |
| TCGA.56.7579.11A | LUSC | 959.8131324 | 2749.798678 | 3709.61181 | 0.408929915 |
| TCGA.79.5596.01A | LUSC | -945.1196907 | -330.553595 | -1275.673286 | 0.914010921 |
| TCGA.18.3421.01A | LUSC | -330.5332199 | 1152.814669 | 822.2814493 | 0.748042623 |
| TCGA.56.8201.01A | LUSC | 1140.233873 | 2492.222738 | 3632.456611 | 0.419238722 |
| TCGA.18.3408.01A | LUSC | 106.4350682 | 988.452271 | 1094.887339 | 0.720894659 |
| TCGA.22.5478.11A | LUSC | 1011.971842 | 2688.486695 | 3700.458537 | 0.410155664 |
| TCGA.85.8276.01A | LUSC | 932.0274937 | 1625.525706 | 2557.5532 | 0.556685033 |
| TCGA.66.2773.01A | LUSC | 16.93683179 | 1442.727827 | 1459.664658 | 0.682770075 |
| TCGA.NK.A5CR.01A | LUSC | -1306.970929 | -501.652356 | -1808.623285 | 0.942920531 |
| TCGA.66.2742.01A | LUSC | -35.43634136 | 915.0046076 | 879.5682663 | 0.742435576 |
| TCGA.43.7656.01A | LUSC | 187.1190218 | 1720.190728 | 1907.30975 | 0.633321891 |
| TCGA.68.A59J.01A | LUSC | -323.9575006 | 1067.158038 | 743.2005379 | 0.755695815 |
| TCGA.98.A53D.01A | LUSC | 1371.473657 | 2128.411656 | 3499.885313 | 0.436825432 |
| TCGA.51.6867.01A | LUSC | -280.3464242 | 516.2621882 | 235.9157639 | 0.802324485 |
| TCGA.43.A56U.01A | LUSC | 414.319637 | 2580.465591 | 2994.785228 | 0.502259155 |
| TCGA.43.A474.01A | LUSC | -567.3429934 | -832.1676011 | -1399.510595 | 0.921234085 |
| TCGA.85.A4QR.01A | LUSC | -2227.506328 | -930.3558347 | -3157.862162 | 0.990012665 |
| TCGA.77.7140.01A | LUSC | -327.7539047 | -166.1863402 | -493.9402448 | 0.861549425 |
| TCGA.66.2768.01A | LUSC | -454.3855761 | 1246.25506 | 791.8694844 | 0.750997778 |
| TCGA.33.AASD.01A | LUSC | -811.6743734 | -260.790522 | -1072.464895 | 0.90150496 |
| TCGA.96.8169.01A | LUSC | 56.72276184 | 950.447154 | 1007.169916 | 0.729758229 |
| TCGA.J1.A4AH.01A | LUSC | -1196.945811 | -130.8095949 | -1327.755406 | 0.917085698 |
| TCGA.21.5784.01A | LUSC | 142.3682996 | 1533.445951 | 1675.814251 | 0.659248609 |
| TCGA.22.5481.01A | LUSC | -632.831461 | 1126.03584 | 493.2043785 | 0.779215052 |
| TCGA.63.A5MG.01A | LUSC | -43.72428509 | 1121.263804 | 1077.539519 | 0.722657129 |
| TCGA.43.6143.01A | LUSC | -218.0357725 | -175.0816273 | -393.1173999 | 0.853941969 |
| TCGA.33.4583.01A | LUSC | -1137.128753 | 547.9862604 | -589.1424926 | 0.868559599 |
| TCGA.43.5670.01A | LUSC | -1137.560866 | -637.0993706 | -1774.660236 | 0.941248599 |
| TCGA.21.5787.01A | LUSC | 203.4838701 | 1827.369021 | 2030.852891 | 0.619184288 |
| TCGA.63.7021.01A | LUSC | 215.1119088 | 1289.816061 | 1504.92797 | 0.677900619 |
| TCGA.33.AASI.01A | LUSC | -1008.282156 | 934.0033021 | -74.27885355 | 0.828661618 |
| TCGA.34.5928.01A | LUSC | 1152.539845 | 825.6233209 | 1978.163166 | 0.625238987 |
| TCGA.56.7730.01A | LUSC | -1743.768728 | -904.931442 | -2648.70017 | 0.976722089 |
| TCGA.77.7335.01A | LUSC | 1863.360015 | 2388.248507 | 4251.608522 | 0.335110315 |
| TCGA.56.7223.01A | LUSC | -1320.76347 | -1391.674637 | -2712.438106 | 0.978686252 |
| TCGA.22.5483.11A | LUSC | 1074.426153 | 2268.404527 | 3342.83068 | 0.457445477 |
| TCGA.98.7454.01A | LUSC | 1664.508613 | 2415.390122 | 4079.898735 | 0.358748984 |
| TCGA.56.8201.11A | LUSC | 1021.81582 | 2664.071172 | 3685.886992 | 0.412105465 |
| TCGA.O2.A5IB.01A | LUSC | -1475.018588 | -1065.147353 | -2540.165941 | 0.973180824 |
| TCGA.77.8008.11A | LUSC | 1682.70428 | 2319.525647 | 4002.229927 | 0.369367408 |
| TCGA.34.8456.01A | LUSC | -453.8776757 | 333.5293369 | -120.3483389 | 0.832427931 |
| TCGA.94.7557.01A | LUSC | -206.5342672 | 489.9975244 | 283.4632572 | 0.798139044 |

Supplementary Table 11 ESTIMATE

| | | | | | |
|---|---|---|---|---|---|
| TCGA.63.5131.01A | LUSC | -693.6766386 | 246.5274965 | -447.1491421 | 0.858042229 |
| TCGA.85.A4CL.01A | LUSC | -211.5079412 | 1419.401246 | 1207.893305 | 0.709299807 |
| TCGA.22.5479.01A | LUSC | -262.9116256 | 614.4892926 | 351.577667 | 0.792075479 |
| TCGA.63.7020.01A | LUSC | -301.4876995 | 1149.598788 | 848.111088 | 0.745521026 |
| TCGA.98.A53H.01A | LUSC | 1161.390937 | 2185.066886 | 3346.457823 | 0.456971962 |
| TCGA.85.A50M.01A | LUSC | -37.71732551 | 352.4688217 | 314.7514962 | 0.795363627 |
| TCGA.O2.A52V.01A | LUSC | -195.5847395 | 848.9375856 | 653.3528461 | 0.764267357 |
| TCGA.56.1622.01A | LUSC | -796.646349 | -423.987907 | -1220.634256 | 0.910703524 |
| TCGA.92.7340.11A | LUSC | 1105.257268 | 2336.695971 | 3441.953239 | 0.444459068 |
| TCGA.37.4130.01A | LUSC | -15.83163122 | 2622.40742 | 2606.575789 | 0.550692828 |
| TCGA.85.A512.01A | LUSC | -467.4211145 | 78.05925073 | -389.3618637 | 0.853654976 |
| TCGA.77.8138.01A | LUSC | 356.8332252 | 1079.290161 | 1436.123386 | 0.68529076 |
| TCGA.85.7710.01A | LUSC | -660.9966162 | 651.0742552 | -9.922361029 | 0.823336884 |
| TCGA.66.2765.01A | LUSC | 140.6805565 | 1392.093119 | 1532.773676 | 0.674890078 |
| TCGA.56.8625.01A | LUSC | 1289.313109 | 2560.767365 | 3850.080474 | 0.390027974 |
| TCGA.66.2781.01A | LUSC | -92.3757578 | 681.500759 | 589.1250012 | 0.770313351 |
| TCGA.22.4591.01A | LUSC | -996.542985 | 23.5771441 | -972.9658409 | 0.895088313 |
| TCGA.66.2755.01A | LUSC | 567.1928528 | 2256.429189 | 2823.622042 | 0.52382417 |
| TCGA.63.6202.01A | LUSC | 774.7517842 | 2331.050074 | 3105.801858 | 0.4881017 |
| TCGA.85.8479.01A | LUSC | -1084.651952 | -419.4961366 | -1504.148088 | 0.927100191 |
| TCGA.66.2767.01A | LUSC | 674.6087185 | 2455.638558 | 3130.247277 | 0.48496674 |
| TCGA.77.7142.01A | LUSC | -197.4421375 | 396.0058263 | 198.5636888 | 0.805585058 |
| TCGA.22.5477.01A | LUSC | -839.6162992 | 200.9093703 | -638.7069289 | 0.872142199 |
| TCGA.85.6798.01A | LUSC | -82.713128 | -343.844076 | -426.557204 | 0.856485938 |
| TCGA.56.7731.11A | LUSC | 839.2465709 | 2381.055596 | 3220.302167 | 0.473364247 |
| TCGA.98.A53A.01A | LUSC | -1190.042528 | -30.30111639 | -1220.343644 | 0.910685903 |
| TCGA.34.5239.01A | LUSC | 1107.893408 | 1759.432555 | 2867.325963 | 0.518348768 |
| TCGA.56.8308.01A | LUSC | -51.57949835 | 599.8039417 | 548.2244433 | 0.774127815 |
| TCGA.L3.A524.01A | LUSC | -558.966214 | -246.2786251 | -805.2448391 | 0.88384061 |
| TCGA.37.3789.01A | LUSC | -174.980747 | 1338.399245 | 1163.418498 | 0.713886564 |
| TCGA.77.8007.11A | LUSC | 1413.100441 | 2773.755716 | 4186.856157 | 0.344050355 |
| TCGA.77.8128.01A | LUSC | -73.25708088 | -790.881423 | -864.1385039 | 0.887851518 |
| TCGA.60.2713.01A | LUSC | 1033.878417 | 1720.785395 | 2754.663812 | 0.532419603 |
| TCGA.77.8146.01A | LUSC | -285.4975085 | 504.994548 | 219.4970395 | 0.803760701 |
| TCGA.NC.A5HM.01A | LUSC | -1200.276628 | 409.8133198 | -790.4633087 | 0.882823543 |
| TCGA.92.7341.01A | LUSC | 204.2511689 | 286.1523094 | 490.4034782 | 0.779472671 |
| TCGA.94.8035.01A | LUSC | -1104.243278 | -30.79910745 | -1135.042386 | 0.905442134 |
| TCGA.60.2722.01A | LUSC | -909.05583 | -244.6137756 | -1153.669606 | 0.906599358 |
| TCGA.51.4079.11A | LUSC | 1008.538052 | 1691.35361 | 2699.891662 | 0.539207955 |
| TCGA.77.7465.01A | LUSC | -30.78702958 | 1744.557759 | 1713.77073 | 0.655048994 |
| TCGA.66.2759.01A | LUSC | 51.60180189 | 1227.979519 | 1279.581321 | 0.701842959 |
| TCGA.68.A59I.01A | LUSC | 427.0158884 | 1268.723644 | 1695.739533 | 0.657046562 |
| TCGA.77.A5G7.01B | LUSC | -1418.067073 | -278.0710797 | -1696.138153 | 0.937293621 |
| TCGA.77.8148.01A | LUSC | -63.54627355 | 220.7424577 | 157.1961841 | 0.809167883 |
| TCGA.39.5039.01A | LUSC | 634.3618114 | 860.1950583 | 1494.55687 | 0.679018999 |
| TCGA.43.8118.01A | LUSC | 4.889309356 | 981.8261344 | 986.7154438 | 0.731807747 |
| TCGA.22.4596.01A | LUSC | 1310.181328 | 2638.28995 | 3948.471278 | 0.376688943 |
| TCGA.18.3406.01A | LUSC | 719.8523197 | 1677.110872 | 2396.963191 | 0.576111 |
| TCGA.56.7730.11A | LUSC | 288.3506337 | 1487.005483 | 1775.356117 | 0.648191805 |

**Supplementary Table 12: Correlation analysis between ACOX2 and markers of immune cells exhaustion in TIMER 2.0**

| | LUAD | | LUSC | |
|---|---|---|---|---|
| | Partial Cor. | *p-value* | Partial Cor. | *p-value* |
| PD-1 (PDCD1) | -0.100787638 (Neg) | **0.025229593** | 0.197463131 (Pos) | **$1.40 \times 10^{-05}$** |
| CTLA4 | -0.041209237 (ns) | 0.361211631 | 0.176637067 (Pos) | **0.000105221** |
| LAG3 | -0.20380736 (Neg) | **$5.07 \times 10^{-06}$** | 0.088418971 (ns) | 0.053629899 |
| TIM-3 (HAVCR2) | -0.034660876 (ns) | 0.442562342 | 0.343220747 (Pos) | **$1.24 \times 10^{-14}$** |
| GZMB | -0.271384276 (neg) | **$9.03 \times 10^{-10}$** | 0.097748701 (ns) | 0.032812002 |

Analysis was conducted using TIMER 2.0 [8]. Results are presented as purity-corrected partial Spearman's rho value and statistical significance. Neg – negative correlation (p<0.05, p<0); Pos – Positive correlation (p<0.05, p>0); ns – not significant (p>0.05)

# References


1. Cai L, Lin S, Girard L, Zhou Y, Yang L, Ci B, Zhou Q, Luo D, Yao B, Tang H *et al*: **LCE: an open web portal to explore gene expression and clinical associations in lung cancer**. *Oncogene* 2019, **38**(14):2551-2564.
2. **Comprehensive molecular profiling of lung adenocarcinoma**. *Nature* 2014, **511**(7511):543-550.
3. **Comprehensive genomic characterization of squamous cell lung cancers**. *Nature* 2012, **489**(7417):519-525.
4. Chandrashekar DS, Bashel B, Balasubramanya SAH, Creighton CJ, Ponce-Rodriguez I, Chakravarthi B, Varambally S: **UALCAN: A Portal for Facilitating Tumor Subgroup Gene Expression and Survival Analyses**. *Neoplasia* 2017, **19**(8):649-658.
5. cProSite: **A web based interactive platform for on-line proteomics and phosphoproteomics data analysis of the datasets of the National Cancer Institute's Clinical Proteomic Tumor Analysis Consortium (CPTAC) and the National Cancer Institute's International Cancer Proteogenome Consortium (ICPC).** https://cprositeccrcancergov/#/.
6. Koch A, Jeschke J, Van Criekinge W, van Engeland M, De Meyer T: **MEXPRESS update 2019**. *Nucleic Acids Res* 2019, **47**(W1):W561-W565.
7. Díez-Villanueva A, Mallona I, Peinado MA: **Wanderer, an interactive viewer to explore DNA methylation and gene expression data in human cancer**. *Epigenetics Chromatin* 2015, **8**:22.
8. Li T, Fu J, Zeng Z, Cohen D, Li J, Chen Q, Li B, Liu XS: **TIMER2.0 for analysis of tumor-infiltrating immune cells**. *Nucleic Acids Res* 2020, **48**(W1):W509-W514.
