# Supplementary figures and images for: Altered expression of ACOX2 in non-small cell lung cancer
Source: BMC Pulm Med. 2022 Aug 23;22:321. doi: 10.1186/s12890-022-02115-7 (PMC9396774; doi:10.1186/s12890-022-02115-7)

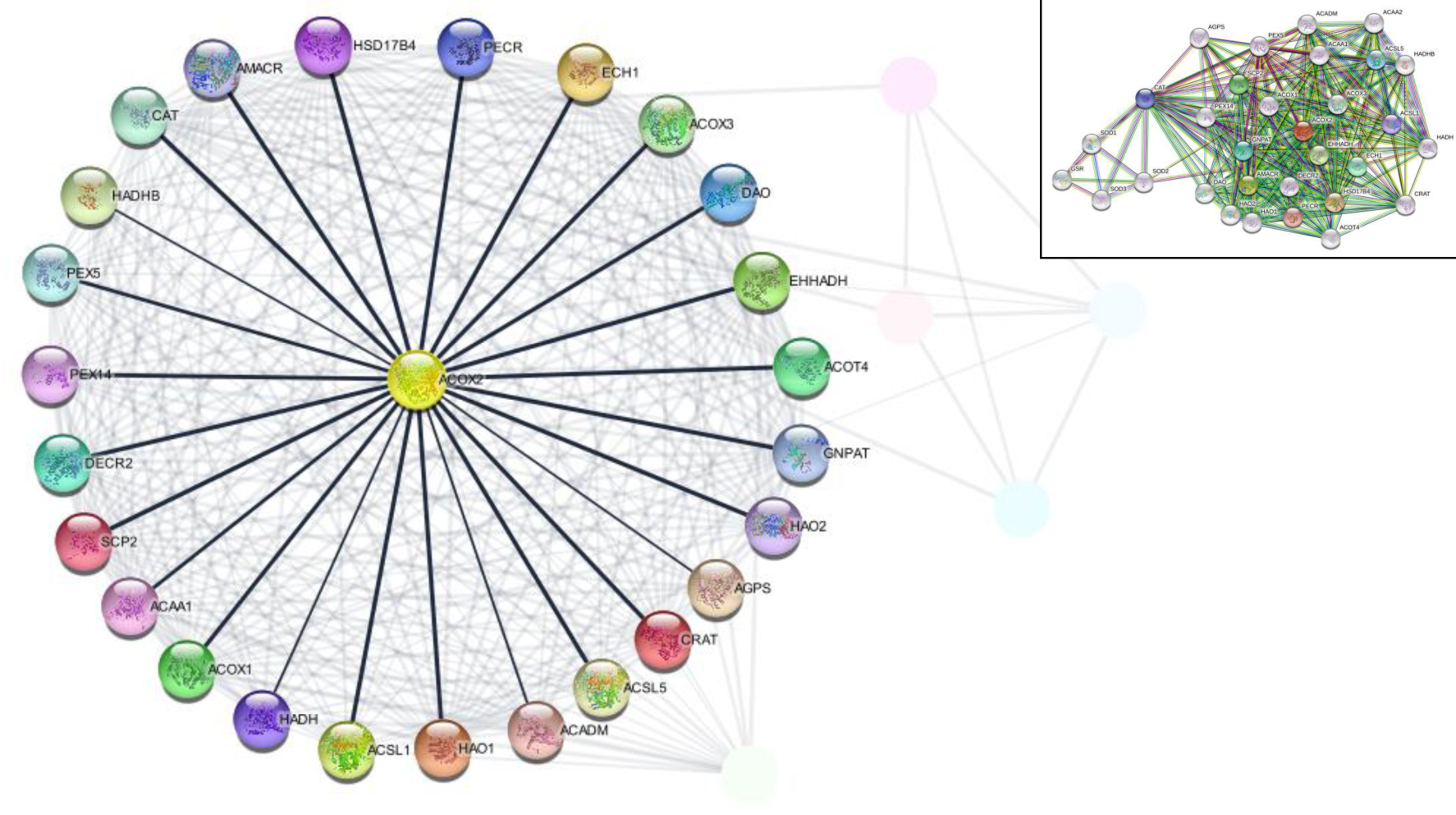

Supplement: Supplementary file 2 — Additional file 2: Figure S1. Protein-Protein Interaction Networks associated with acyl-CoA oxidases. Functional enrichment analysis of acyl-CoA oxidases was carried out on the STRING database [34] to identify Protein-Protein Interaction Networks. The results were imported into Cytoscape [34] and first neighbours highlighted. The resulting first-neighbours are shown. [file 12890_2022_2115_MOESM2_ESM.tif]

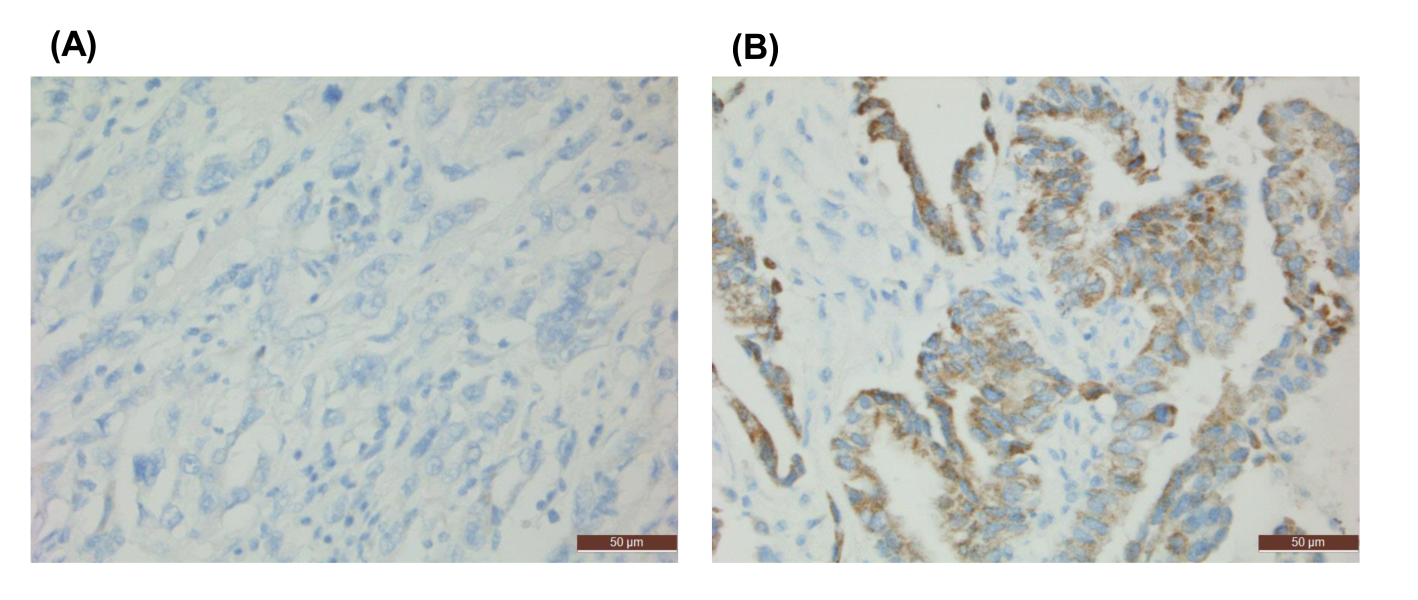

Supplement: Supplementary file 3 — Additional file 3: Figure S2. IHC of ACOX2. Representative examples of ACOX2 protein expression in NSCLC. (A) Negative staining (B) Positive Staining . Images were taken at 40x. [file 12890_2022_2115_MOESM3_ESM.tif]

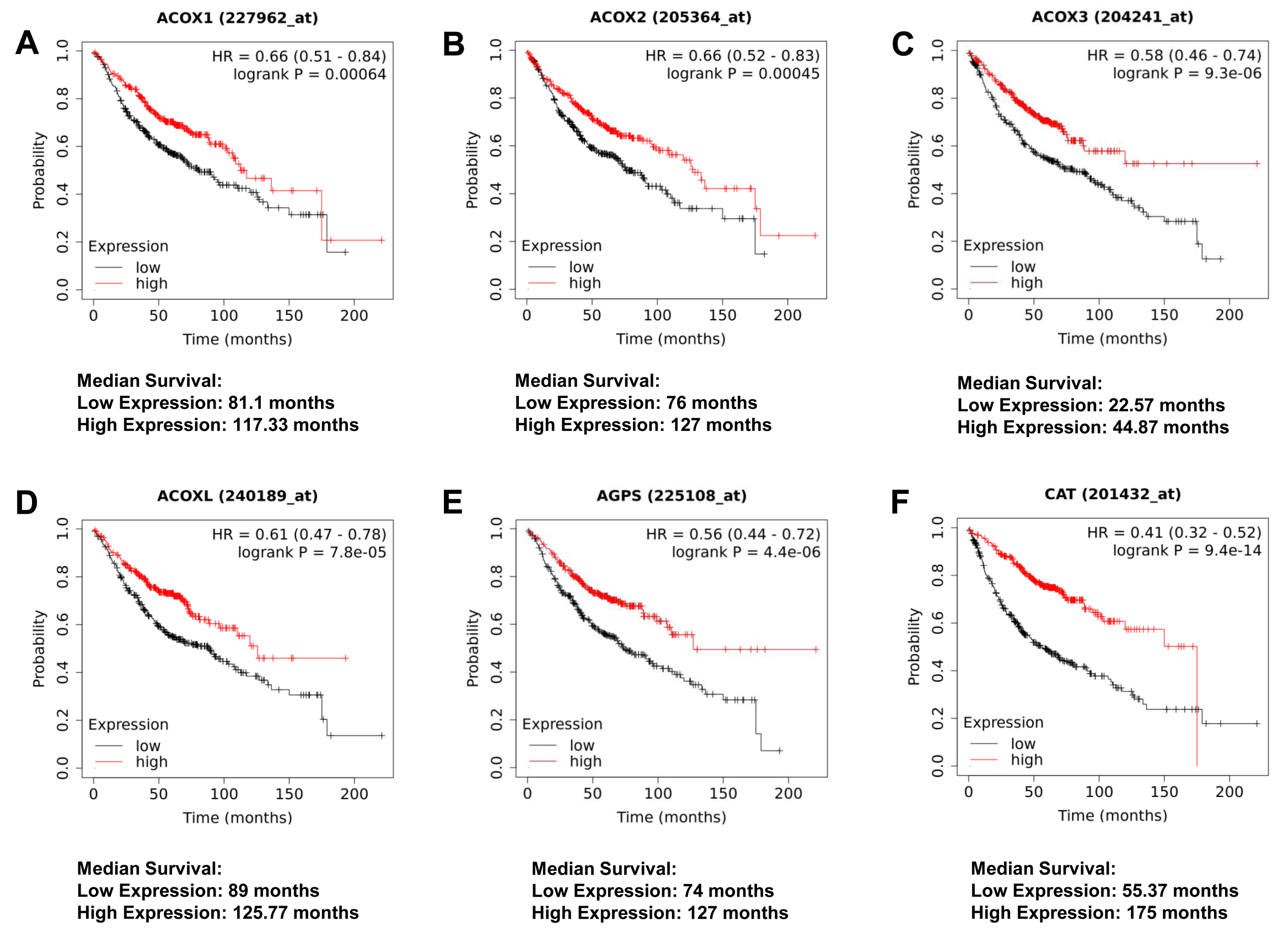

Supplement: Supplementary file 4 — Additional file 4: Figure S3. Prognostic value of acyl-CoA oxidases and other peroxisomal genes in NSCLC. The prognostic effects of ACOX2, other acyl-CoA oxidases and key other peroxisomal associated genes were assessed for overall survival (OS) using KM-Plotter [38]. Analyses were conducted on the gene chip datasets for LUAD. Higher expression of the mRNA for (A) ACOX1, (B) ACOX2, (C) ACOX3, (D) ACOXL, (E) AGPS and (F) CAT was found to be associated with significantly better OS in LUAD, with p<0.05 considered to be significant. [file 12890_2022_2115_MOESM4_ESM.tif]

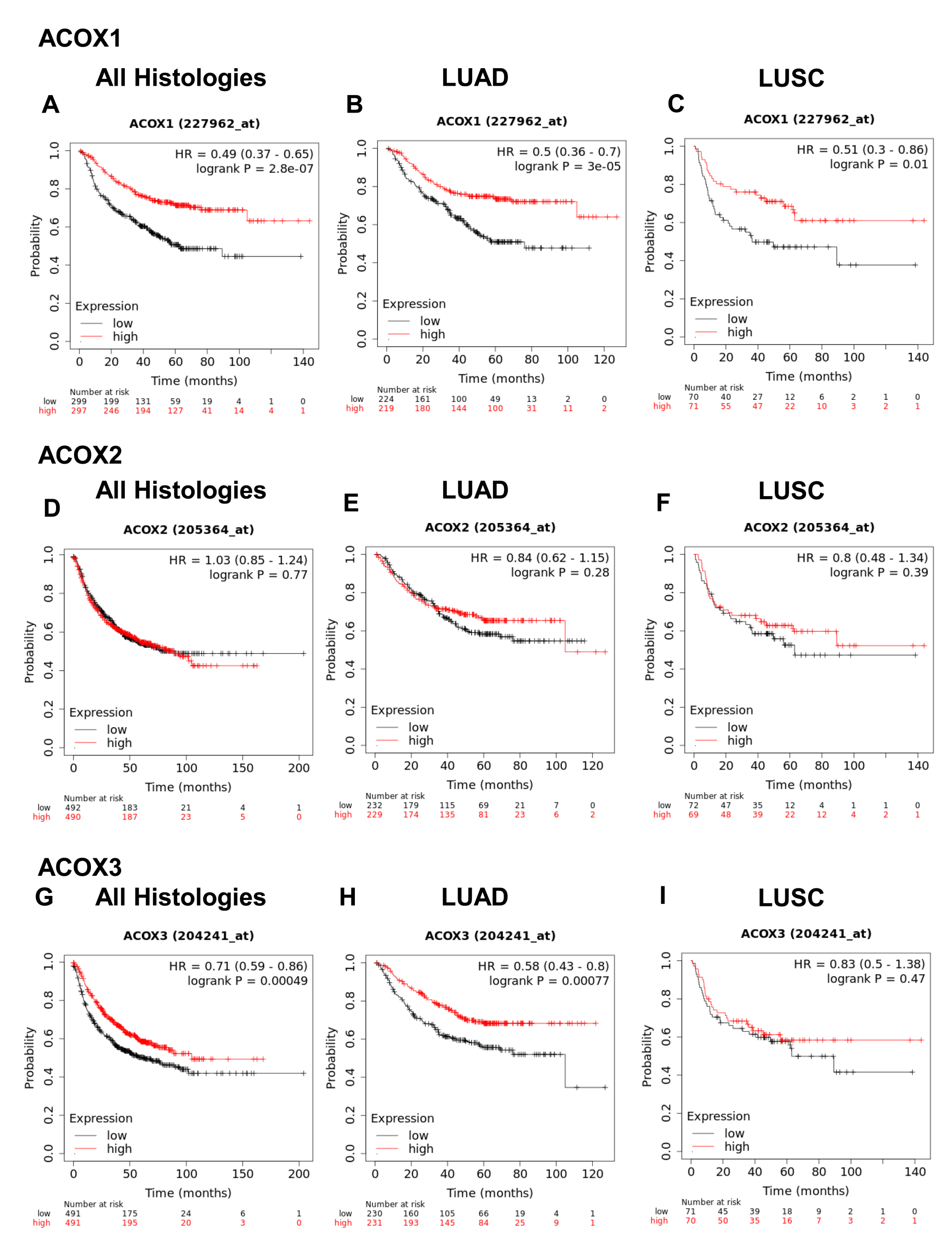

Supplement: Supplementary file 5 — Additional file 5: Figure S4. Prognostic value of acyl-CoA oxidases mRNA expression in NSCLC as assessed by Progression Free Survival. Progression free survival (PFS) is defined First Progression (FP) and KM-Plot was used to analyse ACOX1-3 across the NSCLC. The results are presented as follows: (A) ACOX1 – all histologies; (B) ACOX1 – LUAD; (C) ACOX1 – LUSC; (D) ACOX2 – all histologies; (E) ACOX2 – LUAD; (F) ACOX2 – LUSC; (G) ACOX3 – all histologies; (H) ACOX3 – LUAD; (I) ACOX3 – LUSC. [file 12890_2022_2115_MOESM5_ESM.tif]

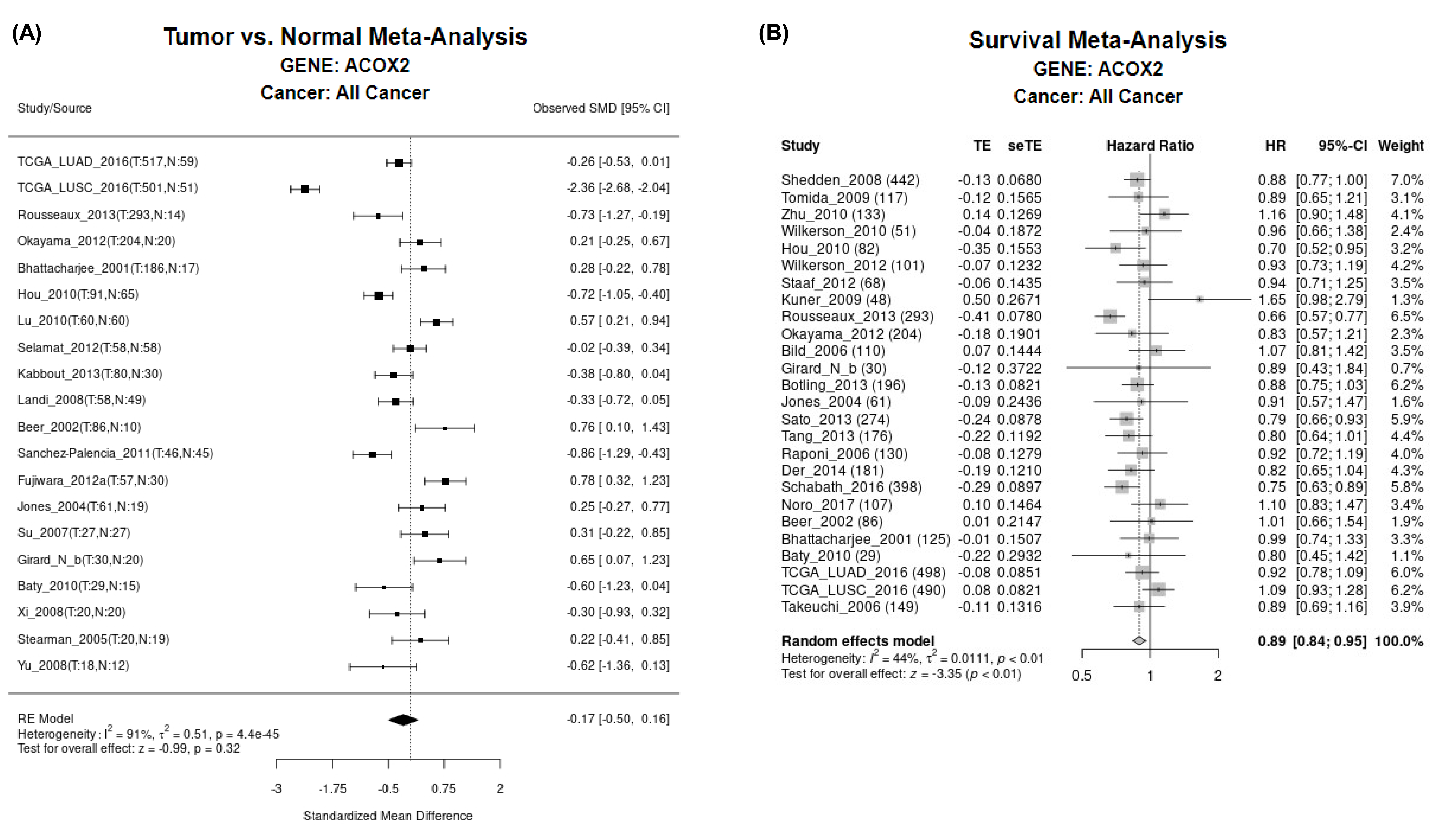

Supplement: Supplementary file 6 — Additional file 6: Figure S5. Meta-analysis of ACOX2 expression in multiple NSCLC datasets. A meta-analysis was conducted on LCE [33] for a large number of gene expression datasets generating forest plots summarizing (A) tumour - normal standardized mean difference for tumour vs normal meta-analysis and (B) hazard ratios for OS meta-analysis for all NSCLC datasets. [file 12890_2022_2115_MOESM6_ESM.tif]

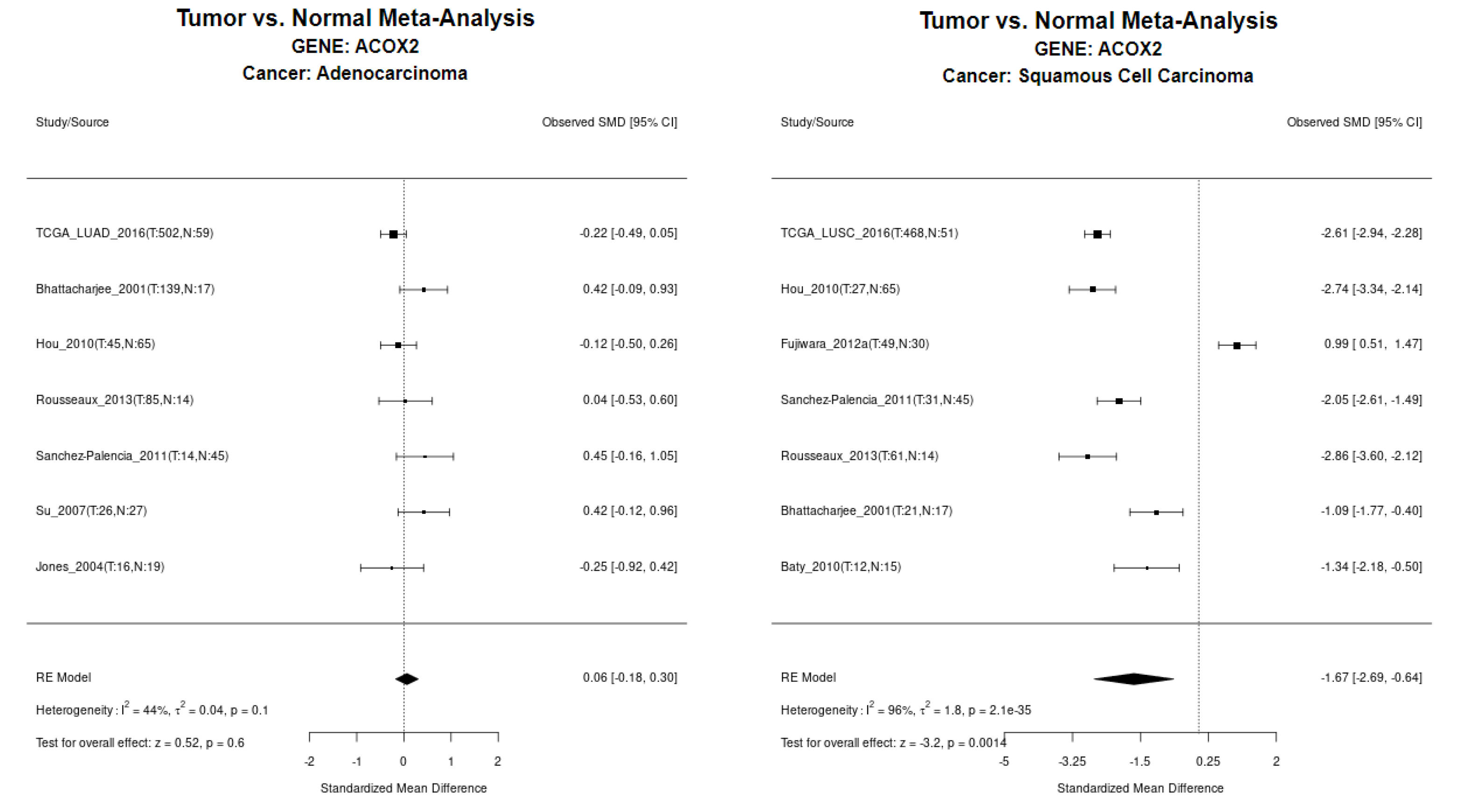

Supplement: Supplementary file 7 — Additional file 7: Figure S6. Meta-analysis of ACOX2 expression in multiple NSCLC datasets. Meta-analysis conducted on LCE [32] generating forest plots summarizing hazard ratios for OS in (A) LUAD and (B) LUSC specific datasets. [file 12890_2022_2115_MOESM7_ESM.tif]
